# Mining the candidate genes of rice panicle traits via a genome-wide association study

**DOI:** 10.3389/fgene.2023.1239550

**Published:** 2023-09-04

**Authors:** Zhengbo Liu, Hao Sun, Yanan Zhang, Mingyu Du, Jun Xiang, Xinru Li, Yinping Chang, Jinghan Sun, Xianping Cheng, Mengyuan Xiong, Zhe Zhao, Erbao Liu

**Affiliations:** College of Agronomy, Anhui Agricultural University, Hefei, China

**Keywords:** rice, panicle traits, genome-wide association study, favorable haplotypes, candidate genes

## Abstract

Panicle traits are important for improving the panicle architecture and grain yield of rice. Therefore, we performed a genome-wide association study (GWAS) to analyze and determine the genetic determinants of five panicle traits. A total of 1.29 million single nucleotide polymorphism (SNP) loci were detected in 162 rice materials. We carried out a GWAS of panicle length (PL), total grain number per panicle (TGP), filled grain number per panicle (FGP), seed setting rate (SSR) and grain weight per panicle (GWP) in 2019, 2020 and 2021. Four quantitative trait loci (QTLs) for PL were detected on chromosomes 1, 6, and 9; one QTL for TGP, FGP, and GWP was detected on chromosome 4; two QTLs for FGP were detected on chromosomes 4 and 7; and one QTL for SSR was detected on chromosome 1. These QTLs were detected via a general linear model (GLM) and mixed linear model (MLM) in both years of the study period. In this study, the genomic best linear unbiased prediction (BLUP) method was used to verify the accuracy of the GWAS results. There are nine QTLs were both detected by the multi-environment GWAS method and the BLUP method. Moreover, further analysis revealed that three candidate genes, *LOC_Os01g43700*, *LOC_Os09g25784*, and *LOC_Os04g47890*, may be significantly related to panicle traits of rice. Haplotype analysis indicated that *LOC_Os01g43700* and *LOC_Os09g25784* are highly associated with PL and that *LOC_Os04g47890* is highly associated with TGP, FGP, and GWP. Our results offer essential genetic information for the molecular improvement of panicle traits. The identified candidate genes and elite haplotypes could be used in marker-assisted selection to improve rice yield through pyramid breeding.

## 1 Introduction

Rice (*Oryza sativa* L.) is one of the most important food crop species and serves as the primary nutrition source for almost half of the world’s population (Loos et al., 2014). With the increase in the world population, food shortages have become a serious global problem. Therefore, increasing production has become a key target in rice breeding. Rice yield is a complex trait multiplicatively determined by four main factors: number of panicles, number of grains per panicle, grain weight (GW), and proportion of filled grain (Li et al., 2013). Panicle length (PL) is one aspect of panicle architecture and is usually considered a yield-related trait. PL, together with spikelet number and density and seed setting rate (SSR), determines the grain number per panicle (Liu et al., 2016). An increase in grain number per panicle reflects an increase in yield. In addition, GW is an important agronomic trait that directly affects rice yield (Xing and Zhang, 2010). Elucidation of the genetic mechanism controlling these panicle traits would thus be an efficient way for breeders to improve rice yields.

The panicle trait is a quantitative trait regulated by multiple genes and is greatly affected by the environment. In recent years, many quantitative trait loci (QTLs) and genes associated with panicle traits have been mapped and cloned, which is inseparable from the rapid development of molecular biology techniques however, the molecular mechanisms underlying panicle traits have been insufficiently studied. To date, at a minimum 300 QTLs for panicle traits distributed across 12 chromosomes have been detected (Xing et al., 2002; Kobayashi et al., 2003; Thomson et al., 2003; Mei et al., 2005; Zhang et al., 2015; Zhang et al., 2015; Wang et al., 2019; Bai et al., 2021; Thapa et al., 2021; Zhong et al., 2021). For instance, Agata et al. constructed near-isogenic lines with ST-1 and Koshihikari as genetic backgrounds. Through QTL analysis and fine mapping, two QTLs, *Prl5* and *Pbl6*, were found to independently regulate panicle length in rice (Agata et al., 2020). Zhang et al. conducted a GWAS on 5 panicle traits of 315 rice accessions introduced from the international rice microcore germplasm bank and detected five QTLs related to PL (Zhang et al., 2015). Zhang et al. mapped a QTL regulating rice panicle length, qPL8, by constructing recombinant inbred lines and fine mapping (Zhang et al., 2021). Using 42 single-segment substitution lines (SSSLs) derived from nine donors in the genetic background of HJX74, Wang et al. performed a QTL analysis for PL, and fourteen QTLs for PL were recognized (Wang et al., 2019). Zhong et al. performed a GWAS on 27 traits of 421 homozygous rice accessions, the results of which revealed 21 and 15 QTLs tightly linked to total grain number per panicle (TGP) and PL, respectively (Zhong et al., 2021). In addition, many panicle trait genes are pleiotropic. For example, *OsPIN5b* (Lu et al., 2015) controls PL and affects the SSR and yield of rice; similarly, *Ghd7* (Xue et al., 2008) is a major QTL that controls grain number per panicle, plant height and heading date. Genes including *OsFAD8*, *OsPIN5b*, *HD1*, *Ghd7,* and *EUI1* were found to regulate PL (Xue et al., 2008; Zhang et al., 2008; Huang et al., 2012; Lu et al., 2015; Zhang et al., 2019); among them, the *EUI1* gene positively regulates PL, and the *OsFAD8*, *OsPIN5b*, *HD1*, and *Ghd7* genes negatively regulate PL. A total of five genes controlling grain number per panicle, GW, and SSR have been cloned (Song et al., 2007; Si et al., 2016; Liu et al., 2018; Xiang et al., 2019; Tu et al., 2022). Specifically, *GLW7* encodes a protein that is a positive regulator of cell proliferation and enhances the grain width, grain size, and yield of rice (Si et al., 2016). The GW-related gene *GW2* was found to encode a previously unknown RING-type protein with E3 ubiquitin ligase activity. Loss of *GW2* function enhances the GW, grain with, and yield of rice (Song et al., 2007). Decreased expression of *OsCKX2* leads to the accumulation of cytokinins in inflorescence meristems and increases the number of grains per panicle (Tu et al., 2022). *OsOAT* (Liu et al., 2018) and *LSSR1* (Xiang et al., 2019) are two genes that reportedly regulate the SSR of rice. In the *OsOAT* mutant, metabolic abnormalities induced by nitrogen deficiency in florets result in malformed glumes and anther indehiscence, which affect the pollination process and lead to a low SSR (Liu et al., 2018). *LSSR1* encodes a putative GH5 cellulase that functions during rice fertilization, and *LSSR1* loss-of-function rice mutants presently substantially decreased SSR (Xiang et al., 2019).

In recent years, genome-wide association studies (GWASs) have become an efficient means for mapping QTLs or genes related to target traits. GWASs based on second-generation sequencing technology enable resequencing of many genomes and provide the possibility of identifying favorable alleles and genetic variations associated with complex traits on a large scale with high accuracy (Huang et al., 2010; Huang et al., 2012). Additionally, GWASs are efficient tools for association mapping of phenotypic and genotypic data, which use the advantage of natural variations to establish associations between traits and single-nucleotide polymorphisms (SNPs) (Zhang et al., 2015; Bai et al., 2016; Han et al., 2016). The two models used to perform GWASs include the general linear model (GLM) and the mixed linear model (MLM), and many genes related to panicle traits have been successfully mapped (Huang et al., 2012; Crowell et al., 2016; Su et al., 2016; Bai et al., 2021). For example, Bai et al. used genome-wide association analysis (GWAS) to analyze and identify three agronomic traits of 340 rice materials from the 3,000 Rice Genome Project. A total of 153 quantitative trait QTLs were detected, and three new candidate genes that may affect panicle length were found (Bai et al., 2021). A GWAS based on 5 panicle traits of 315 rice accessions introduced from the international rice microcore germplasm bank revealed a total of 36 SNPs (Zhang et al., 2015). Therefore, the research on the key genes involved in the regulation of panicle traits is still ongoing. Therefore, the genes related to panicle traits need to be further studied. In this research, we used a panel of 162 diverse rice accessions to perform a GWAS on five panicle traits. The goals of this research were to (1) identify QTLs associated with panicle traits, including PL, TGP, filled grain number per panicle (FGP), SSR, and grain weight per panicle (GWP); (2) dissect the genetic architecture of panicle traits and mine the genes; (3) detect beneficial haplotypes; and (4) identify parents with excellent traits and offer molecular information to improve panicle traits by pyramiding breeding.

## 2 Materials and methods

### 2.1 Rice materials

A total of 162 rice accessions were obtained from the State Key Laboratory of Crop and Genetics and Germplasm Innovation of Nanjing Agricultural University (Du et al., 2022). Of the 162 rice germplasms, 128 were from China, and the rest were from other countries, including Vietnam (20), Japan (11), the Philippines (2) and Indonesia (1). More detailed information for each accession, including the accession name, number, latitude, longitude, country of origin and subgroup ancestry of the accession, is given in Supplementary Table S1.

### 2.2 Phenotype identification

The 162 accessions were planted at the Jiangpu Experimental Station of Nanjing Agricultural University in Nanjing, China, in 2019 and at the Experimental Station of Anhui Agricultural University in Hefei, China, in 2020 and 2021. The varieties were sown in the middle of May and transplanted in the middle of June; each single plant was spaced 26 cm × 16 cm in all 3 years (2019–2021). A randomized block design with two replications was used for the 3-year experiments. Each variety was planted in 5 rows, with 8 plants in each row. These varieties were subjected to normal fertilizer and water management practices. At maturity, we harvested five panicles of the main culm of each accession and measured the PL, TGP, FGP, SSR, and GWP. The mean phenotypes of each accession were used to calculate the SSR, FGP, and TGP.

$$SSR\left(\%\right)=\left(FGP∕TGP\right)\times 100\%$$

Excel 2010 (Microsoft) software was used for data collation, SPSS software (version 25.0) was used to process the sorted data, and the mean and standard deviation of each agronomic trait were calculated.

Generalized heritability refers to that all genetic variation accounts for the total phenotypic variation. The calculation formula of broad-sense heritability is determined by comparing the relationship between genetic differences among individuals and total variation. In a population, the genetic differences of individuals can be divided into two parts: one is caused by genetic differences, namely, genetic variance (VA), and the other is caused by environmental factors, namely, environmental variance (VE), Total variation (VP) is the sum of genetic variance (VA) and environmental variance (VE).

The calculation formula of broad heritability is as follows:

$$H_{B}^{2}=VA\;/\;VP\times 100\%$$

The value range of generalized heritability is between 0 and 1. The closer the value is to 1, the greater the influence of genetic factors on the trait is. On the contrary, if the generalized heritability is close to 0, it indicates that the trait is mainly affected by environmental factors.

In this study, we performed a correlation analysis on the 3-year data of the five traits. Regarding the threshold, when | r | ≥ 0.8, it can be considered that the two variables are highly correlated; 0.5 ≤ | r | < 0.8, it can be considered that the two variables are moderately correlated; 0.3 ≤ | r | < 0.5, it can be considered that the two variables are weakly correlated; and | r | < 0.3, the two variables can be considered basically irrelevant.

### 2.3 SNP filter analysis and annotation

To sequence the 162 accessions, young leaves were collected from a single plant at the tillering stage, and genomic DNA was extracted using a standard cetyltrimethylammonium bromide protocol. A double-end sequencing library was constructed using 5 μg genomic DNA, and the inserted fragment was approximately 350 bp reads. After further processing of the original sequence, the genomic sequence data of 0.532 Tb was obtained after removing joint contamination and low-quality reads. The average genome coverage of each test material was 5.48 times.

Accessions in our population were filtered using PLINK (version 1.9) command and SNPs were further filtered using plink with maf (0.05) and geno (0.2). A total of 1,209,388 SNPs were identified. Library construction, sequencing, and sequence cleaning were performed by staff at Mega Genomics Beijing (http://www.megagenomics.cn/mobile.php/, accessed on 10 April 2019).

We used ANNOVAR software (Wang et al., 2010) to perform SNP annotation on the Nipponbare genome sequence. The new annotation results were divided into exon regions, splicing sites, intron regions, and upstream and downstream regions. The SNPs in the coding exons are divided into two types: synonymous and nonsynonymous; nonsynonymous SNPs lead to amino acid changes.

### 2.4 Population structure and genetic analysis

We used PLINK (version 1.9) software to filter the SNP loci of 162 rice materials, retain the unconnected SNP loci, and convert them into STRUCTURE format (Earl and vonHoldt, 2012). To predict the genetic structure of the entire population, we first used STRUCTURE to analyze the population structure of the germplasm and then analyzed the number of required subgroups (K) through the Structure Harvester website (http://taylor0.biology.ucla.edu/structureHarvester, accessed on 8 October2022). (Bradbury et al., 2007; Falush, Stephens, and Pritchard, 2007; Franois and Durand, 2010). The distance matrix was contructed using VCF2Dis (https://github.com/BGI-shenzhen/VCF2Dis, accessed on 9 October 2022) based on SNPs and an NJ clustering diagram was constructedusing MEGA version 7. Principal component analysis was conducted using GCTA (version 1.93.2) software on Linux. PLINK (version 1.9) was used for LD analysis of the genotypic data, and the LD diagram, population structure diagram, NJ tree, and PCA (Zhao et al., 2007) diagram were constructed by R.

### 2.5 GWAS

In this research, we obtained 1,209,388 SNPs (MAF >0.05) and five groups of phenotypic data. Two models, including GLM and MLM (Yu et al., 2006) in TASSEL (version 5.2.40) software (Bradbury et al., 2007), were used to analyze the associations between the SNPs and phenotypic data. The genome-wide significance thresholds of the GWAS were determined using a modified Bonferroni correction (Li et al., 2012), the threshold of the GLM was set to *p* = 4.1 × 10^−8^, and the threshold of MLM was 1.0 × 10^−5^. A QTL covers all SNPs located in the same LD region, and the SNP with the smallest *p*-value is considered the leading SNP. We used the R package ‘CMplot’ to construct the Manhattan diagram.

In this study, the genomic BEST Linear Unbiased Prediction (BLUP) (Habier et al., 2013) method was used to verify the accuracy of the GWAS results. The BLUP method can integrate multi-environment data, remove environmental effects, and obtain stable genetic phenotypes of individuals. BLUP is a common practice for phenotypic processing, and the lmer function in lme4 in R package is a common method for BLUP analysis. We used the R package “CMplot” to construct the Manhattan diagram, and the threshold of the MLM model is 1.0 × 10^−5^, which is the same as the threshold setting of the multi-environment GWAS method.

### 2.6 Identification of candidate genes and haplotype analysis

Based on the LD decay distance and the results of our GWAS analysis, the candidate areas of genes on chromosomes were estimated, and the genes in the candidate regions were identified at the China Rice Data Center. (https://www.ricedata.cn/, accessed on 19 October 2022). Nonsynonymous SNPs lead to amino acid changes, and we focused on nonsynonymous SNPs by comparison with the Nipponbare reference genome sequence (http://rice.plantbiol-ogy.msu.edu/cgi-bin/gbrowse/rice/, accessed on 22 October 2022). The identification of candidate genes was mainly through nonsynonymous SNPs in exons and gene function. The nonsynonymous SNPs in exons were selected for haplotype analysis to select favorable haplotypes. The haplotypes of the candidate genes were analyzed based on geographical region and subgroup. Analysis of different phenotypes of candidate gene haplotypes was performed using ANOVA combined with Duncan’s multiple range test. Favorable haplotype analysis of candidate genes was performed using the 3KRG gcHap dataset (http://bigd.big.ac.cn/gvm/getProjectDetail?project=GVM000123, accessed on 1 March 2023) and the Rice Functional Genomics and Breeding Database (https://www.rmbreeding.cn/Public/download, accessed on 1 March 2023). The “favorable” gcHap of a gene was defined as the one associated with the highest trait value. Five subpopulations of XI (XI-1A, XI-1B, XI-2, XI-3, and XI-adm) and four subpopulations of GJ (temperate GJ [GJ-tmp], subtropical GJ [GJ-sbtrp], tropical GJ [GJ-trp], and GJ-adm) were classified by Wang et al. (2018).

### 2.7 Forecast of excellent parents

The average positive (negative) haplotype effect (AHE) at a gene locus was calculated as follows:

$$AHE=\sum h_{c}∕n_{c}$$

Among them, nc represents the number of haplotypes with positive (negative) effects on gene loci. hc represents the phenotypic value of haplotypes with positive (negative) effects.

Rice materials with the highest positive haplotype effect were predicted to be the most promising parents for panicle trait improvement in rice breeding at all panicle trait-related gene loci.

## 3 Results

### 3.1 Phenotypic variation in panicle traits in the natural population

**FIGURE 1:**
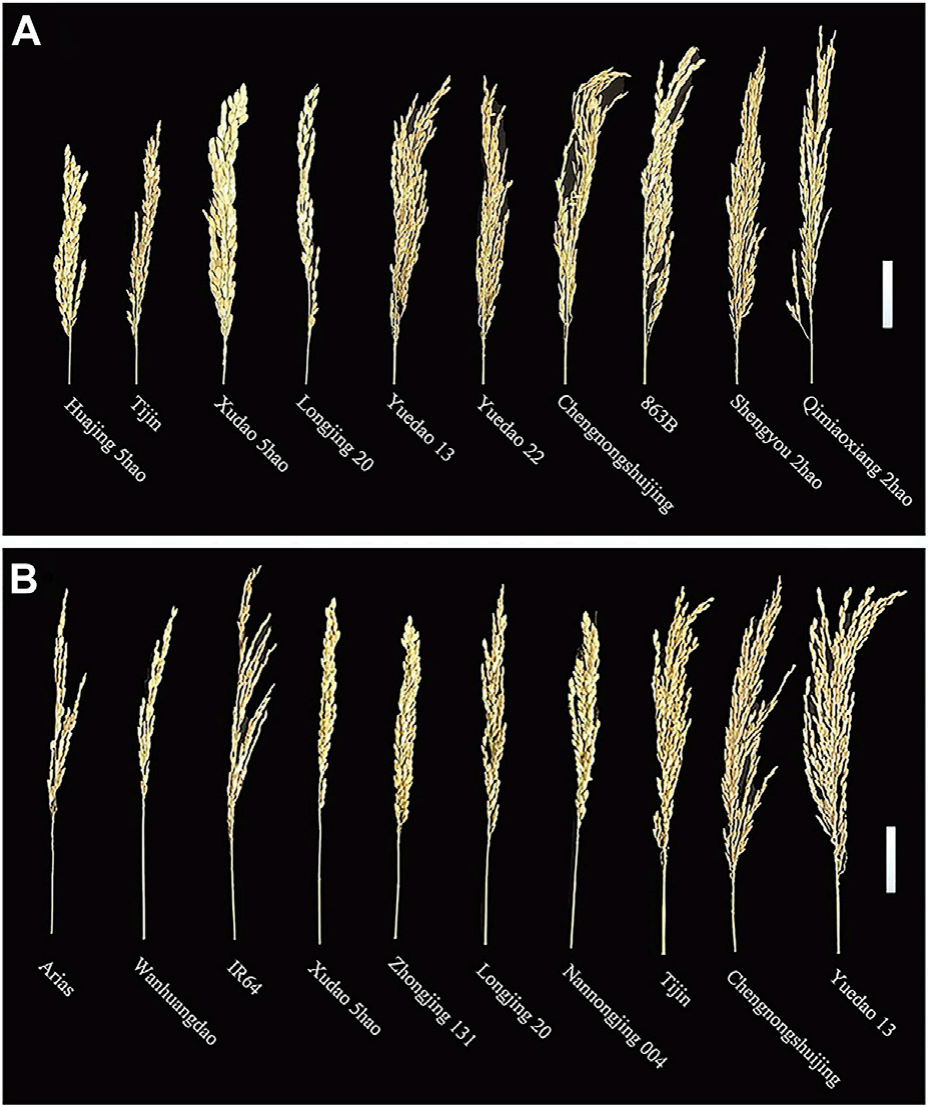
Phenotypes of panicles of 10 rice accessions: **(A)** PL and **(B)** GWP. Scale bar = 5.0 cm.

The panicle traits of 162 rice accessions showed significant differences. Generalized heritability refers to all genetic variation accounting for the total phenotypic variation. All the traits had high generalized heritability, ranging from 73.5% to 98.4% in 2019, 2020 and 2021 (Table 1) Furthermore, it is worth noting that the 3-year broad heritability of PL is greater than 95.0%, which indicates that the variation in this trait is mainly controlled by genes. There were significant differences in PL, TGP, FGP, SSR, and GWP values among varieties in the 3 years, and their coefficients of variation (CV) ranged from 6.7% to 31.2%. Among 162 rice materials, we selected 10 materials to represent the diversity of PL and FWP, including the accession with the greatest PL (Shengyou 2 Hao), the accession with the lowest PL (Huajing 5 Hao), the accession with the greatest GWP (Yuedao 13), and the accession with the lowest GWP (Arias) (Figure 1). The results demonstrated that across the 162 rice accessions, the five panicle traits presented great genetic variation.

**TABLE 1:** Description statistics of panicle traits of 162 rice materials.

Phenotype	Year	Mean ± SD	Range	CV (%)	H_B_^2^ (%)
	2019	22.6 ± 4.7	15.0–35.0	20.8	97.5
PL	2020	23.3 ± 5.1	12.9–38.1	21.7	97.8
	2021	23.2 ± 4.9	12.8–37.7	21.2	98.4
	2019	194.4 ± 54.8	57.0–426.0	28.2	93.6
TGP	2020	196.2 ± 56.9	46.0–406.0	29.0	91.9
	2021	192.0 ± 57.7	53.0–460.0	30.1	87.4
	2019	170.0 ± 51.3	32.0–383.0	30.2	91.6
FGP	2020	181.0 ± 52.8	39.0–375.0	29.2	90.5
	2021	174.7 ± 48.0	43.0–345.0	27.5	97.6
	2019	0.9 ± 0.1	0.4–1.0	12.7	74.0
SSR	2020	0.9 ± 0.1	0.6–1.0	6.7	73.5
	2021	0.9 ± 0.1	0.6–1.0	7.6	94.2
	2019	4.1 ± 1.1	1.3–7.4	27.4	91.9
GWP	2020	4.0 ± 1.1	1.1–7.3	28.4	89.2
	2021	4.1 ± 1.3	0.7–8.2	31.2	79.9

**FIGURE 2:**
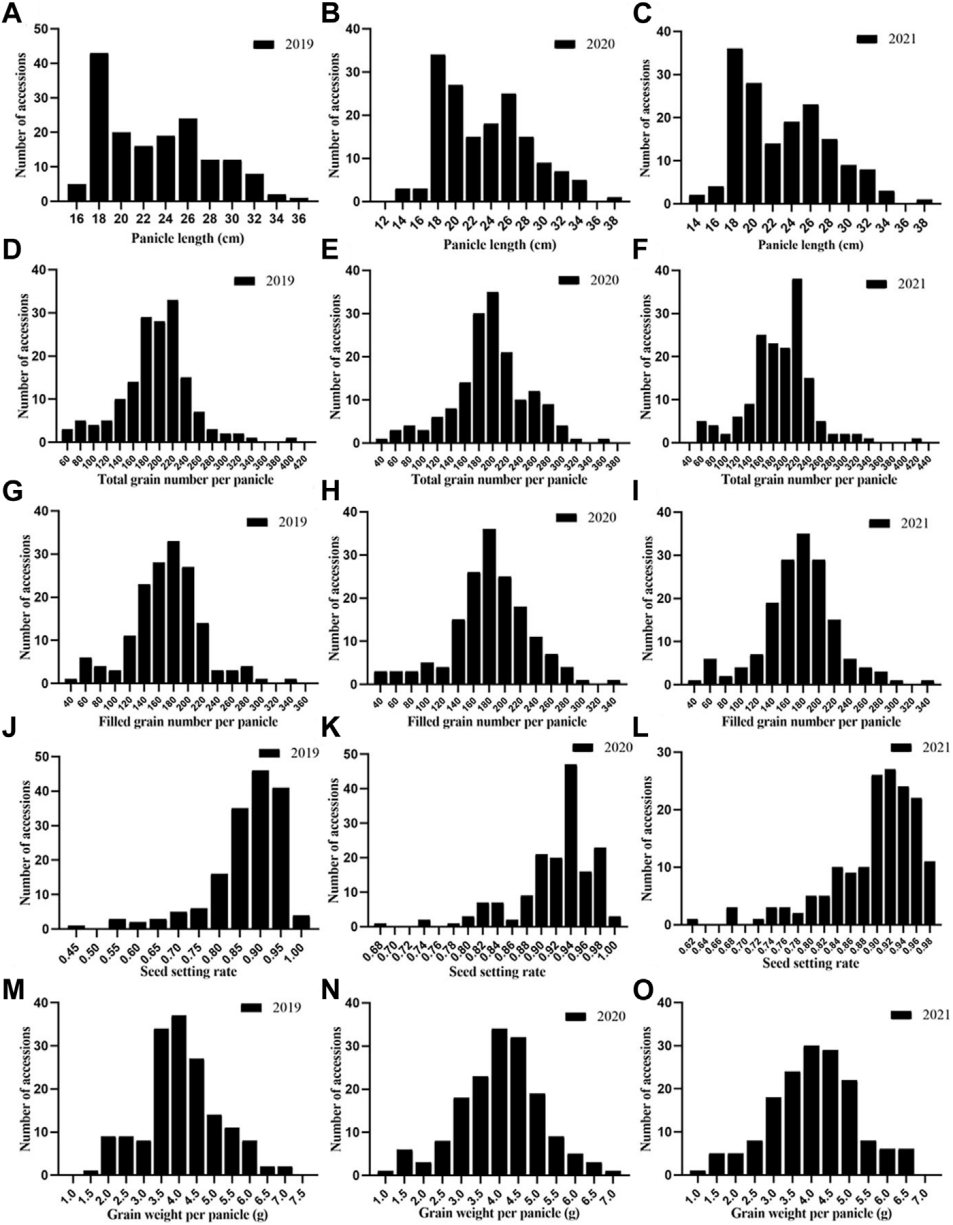
Phenotypic frequency distribution of panicle traits of 162 rice germplasms:**(A)** PL in 2019; **(B)** PL in 2020; **(C)** PL in 2021; **(D)** TGP in 2019; **(E)** TGP in 2020; **(F)** TGP in 2021; **(G)** FGP in 2019; **(H)** FGP in 2020; **(I)** FGP in 2021; **(J)** SSR in 2019; **(K)** SSR in 2020; **(L)** SSR in 2021; **(M)** GWP in 2019; **(N)** GWP in 2020 and **(O)** GWP in 2021.

In general, the phenotypic changes during all 3 years were similar (Figure 2). Most of the traits appeared to be normally distributed in all 3 years, but SSR and PL showed skewed distributions. The TGP, FGP, and GWP were definitively normally distributed, indicating that these three traits were not controlled by a single gene but by quantitative traits, which laid a foundation for better understanding the genetic structure of panicle traits. The results above confirmed that there was abundant phenotypic variation across the 162 rice accessions in this study.

**FIGURE 3:**
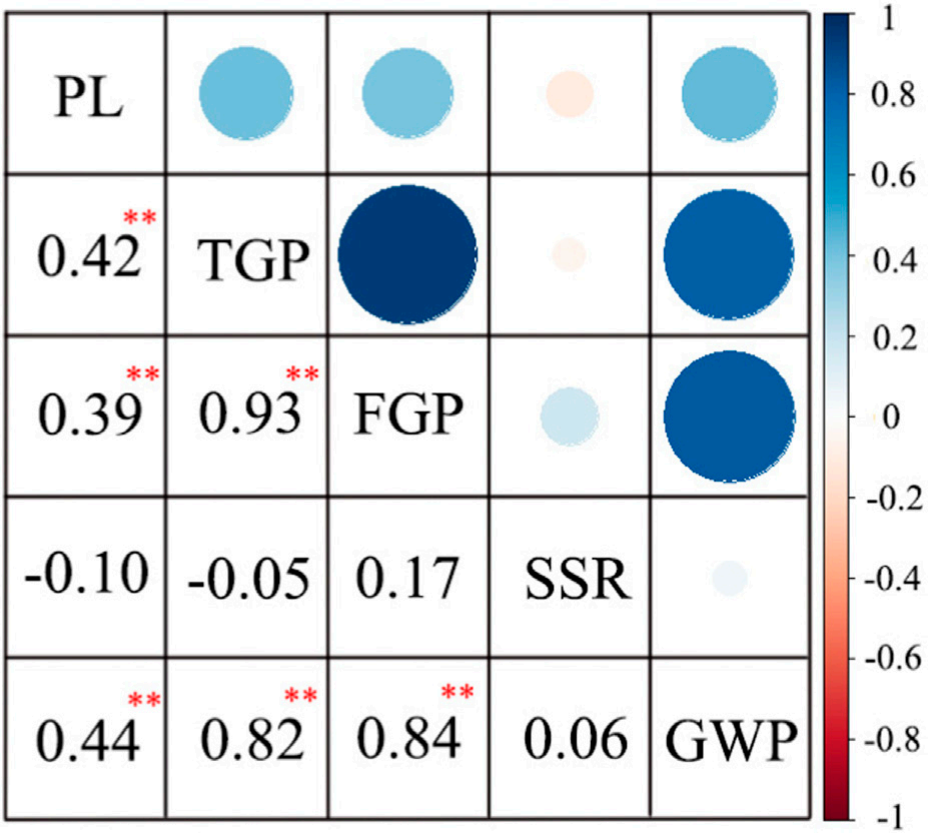
Correlation analysis of panicle traits of 162 rice materials. **, at *p* < 0.01 showed extremely significant difference.

The correlation analysis of the 3-year data showed that SSR was negatively correlated with PL and TGP (Figure 3). However, TGP was positively correlated with FGP and GWP, and FGP was more strongly correlated with TGP than with GWP. In general, TGP, FGP, and GWP mirrored one another and were strongly correlated.

### 3.2 Population structure and linkage disequilibrium (LD) analysis

**FIGURE 4:**
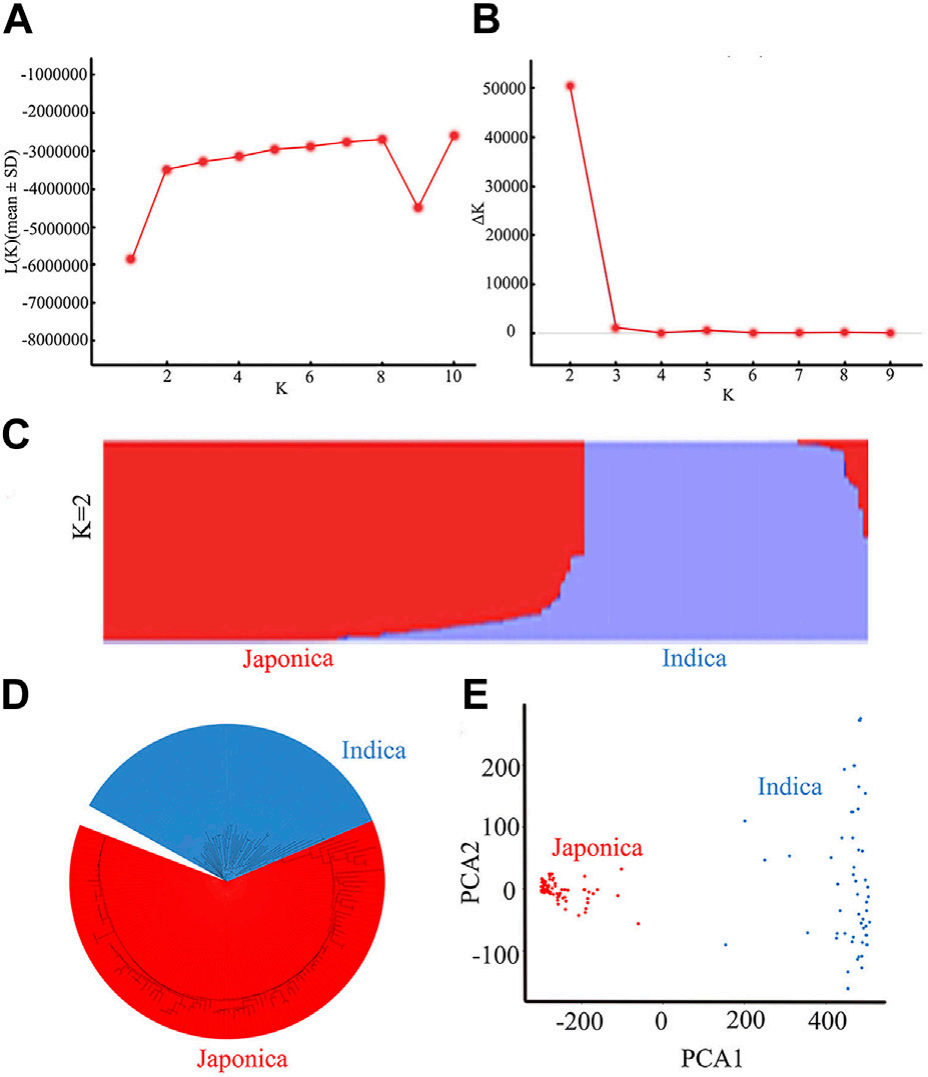
Results of a population genetic structure analysis of a wild population constructed from 162 rice accessions: **(A)** The change in logarithmic likelihood value with the number of subgroups; **(B)** The relationship between ΔK value and the number of subgroups; **(C)** Wild population structure (K = 2); **(D)** NJ tree of the natural rice population; **(E)** Principal component analysis of the natural rice population.

The 162 rice accessions and SNP markers were used for a genetic structure analysis of the natural population (Figure 4; Supplementary Table S2). Using the Bayesian clustering software STRUCTURE (version 2.3.4) (Evanno, Regnaut, and Goudet, 2005), we calculated varying levels of K means (Figure 4A). When ΔK was the highest, K = 2 (Figure 4B). The optimal number of subpopulations was predicted to be K = 2 based on a model component analysis by STRUCTURE software (version 2.3.4) and assessing ΔK. Therefore, the rice accessions of 162 were divided into 2 subgroups, namely, an *indica* group and a *japonica* group (Figure 4C). The 162 rice materials included 59 *indica* subpopulations and 103 *japonica* subpopulations. We used the first two principal components (PCs) as covariates within the GWAS model to control for subpopulation structure (Figure 4E). The results of the principal component analysis (PCA) and neighbor-joining (NJ) tree (Figure 4D) were consistent with the structure results. Therefore, the population of 162 rice materials was divided into two subgroups.

The degree of LD was the chromosome distance when the r2 value was reduced to half of the maximum value. The LD decay distance of the whole population was found to be 112 kb (Du et al., 2022).

### 3.3 GWAS of PL, TGP, FGP, SSR, and GWP traits

**FIGURE 5:**
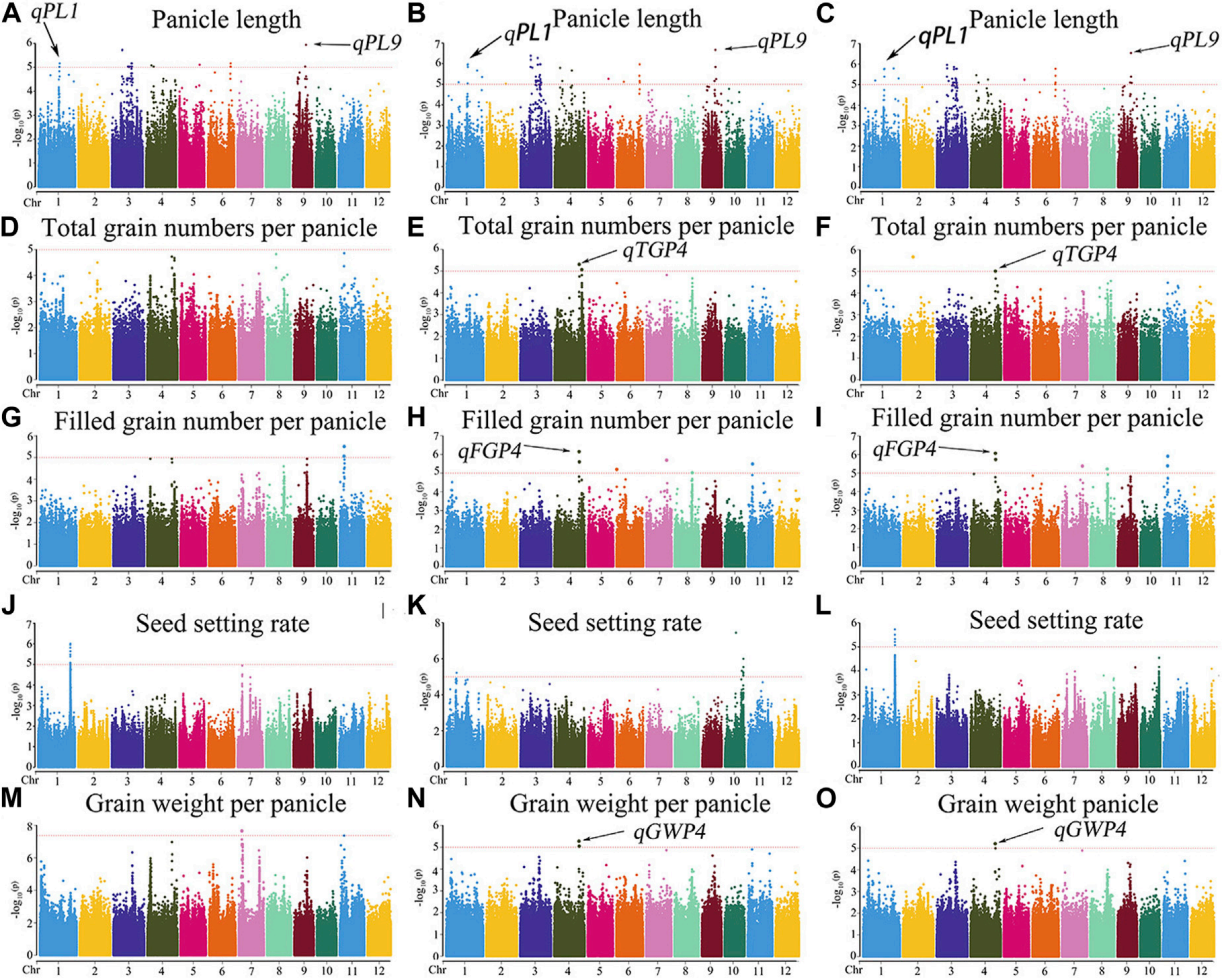
Manhattan plots of the GWAS results for PL, TGP, FGP, SSR, and GWP with MLM: **(A)** PL traits in 2019; **(B)** PL traits in 2020; **(C)** PL traits in 2021; **(D)** TGP traits in 2019; **(E)** TGP traits in 2020; **(F)** TGP traits in 2021; **(G)** FGP traits in 2019; **(H)** FGP traits in 2020; **(I)** FGP traits in 2021; **(J)** SSR traits in 2019; **(K)** SSR traits in 2020; **(L)** SSR traits in 2021; **(M)** GWP traits in 2019; **(N)** GWP traits in 2020; **(O)** GWP traits in 2021.

The MLM with correction of kinship bias was mainly used in this study, supplemented by the GLM. We conducted a GWAS between panicle traits and SNPs across the 162 rice accessions. The GWAS results showed that a total of 14 SNPs (QTLs) were significantly associated with PL, TGP, FGP, SSR, and GWP (Figure 5; Supplementary Figure S1; Supplementary Table S3). These QTLs were located on chromosomes 1, 4, 5, 6, 7, 9, 10, and 11 and were repeatedly detected via at least two models. Among the 14 QTLs, 9 stable QTLs were detected in at least 2 years and via two models, and those QTLs were further analyzed (Table 2). For PL, *qPL1*, *qPL6*, *qPL9.1* and *qPL9.2* were identified on chromosomes 1, 6, and 9, and these QTLs were detected in 3 years and via two models. For SSR, *qSRR1* was identified on chromosome 1 and was detected in 2 years and via two models. For TGP, FGP, and GWP, the Chr4_28,917,371 (*qTGP4*, *qFGP4*, and *qGWP4*) locus on chromosome 4 was found to be associated with TGP, FGP, and GWP simultaneously in at least 2 years and via two models. *qTGP4*, *qFGP4*, and *qGWP4* were the same QTL; hereafter, this QTL is referred to as *qTGP4*. In addition, *qFGP7* was identified on chromosome 7 and was detected in both models for two consecutive years (Table 2).

**TABLE 2:** Genome-wide significant association of rice panicle traits.

Trait	QTLs	Chr	SNP	Allele	2019	2020	2021	Model	Reported QTLs and genes
					*p*-value	*p*-value	*p*-value		
PL	*qPL1*	1	25,009,989	A/G	4.1 × 10^−6^	5.3 × 10^−7^	8.5 × 10^−7^	GLM	
		1	25,009,989	A/G	6.9 × 10^−6^	1.1 × 10^−6^	1.7 × 10^−6^	MLM	
	*qPL6*	6	26,935,985	A/G	4.5 × 10^−6^	8.4 × 10^−6^	7.3 × 10^−6^	GLM	
		6	26,935,985	A/G	9.3 × 10^−6^	7.0 × 10^−6^	8.0 × 10^−6^	MLM	
	*qPL9.1*	9	15,393,518	A/C	1.5 × 10^−6^	3.7 × 10^−7^	3.9 × 10^−7^	GLM	*LP1* Liu et al. (2016)
		9	15,393,518	A/C	1.2 × 10^−6^	2.2 × 10^−7^	2.9 × 10^−7^	MLM	
	*qPL9.2*	9	14,388,593	T/C	4.5 × 10^−6^	8.4 × 10^−6^	8.4 × 10^−6^	GLM	
		9	14,388,593	T/C	9.3 × 10^−6^	7.0 × 10^−6^	7.0 × 10^−6^	MLM	
TGP	*qTGP4*	4	28,917,371	C/T	5.6 × 10^−10^	3.5 × 10^−11^	4.9 × 10^−10^	GLM	
		4	28,917,371	C/T	—	4.9 × 10^−6^	9.7 × 10^−6^	MLM	
	*qFGP4*	4	28,917,371	C/T	3.5 × 10^−8^	4.1 × 10^−11^	2.4 × 10^−10^	GLM	
		4	28,917,371	C/T	—	7.1 × 10^−7^	8.6 × 10^−7^	MLM	
FGP	*qFGP7*	7	23,875,734	C/T	—	9.3 × 10^−11^	2.1 × 10^−9^	GLM	*OsBZR1* Qiao et al. (2017)
		7	23,875,734	C/T	—	2.0 × 10^−6^	4.2 × 10^−6^	MLM	
SSR	*qSSR1.1*	1	37,937,308	G/A	3.5 × 10^−7^	—	2.2 × 10^−6^	GLM	*sd1* Ye et al. (2015)
		1	37,937,308	G/A	2.3 × 10^−6^	—	8.5 × 10^−6^	MLM	
GWP	*qGWP4*	4	28,917,371	C/T	—	7.5 × 10^−10^	1.6 × 10^−9^	GLM	
		4	28,917,371	C/T	—	5.3 × 10^−6^	6.1 × 10^−6^	MLM	

### 3.4 GWAS results of single environment method of PL, TGP, FGP, SSR, and GWP traits

**FIGURE 6:**
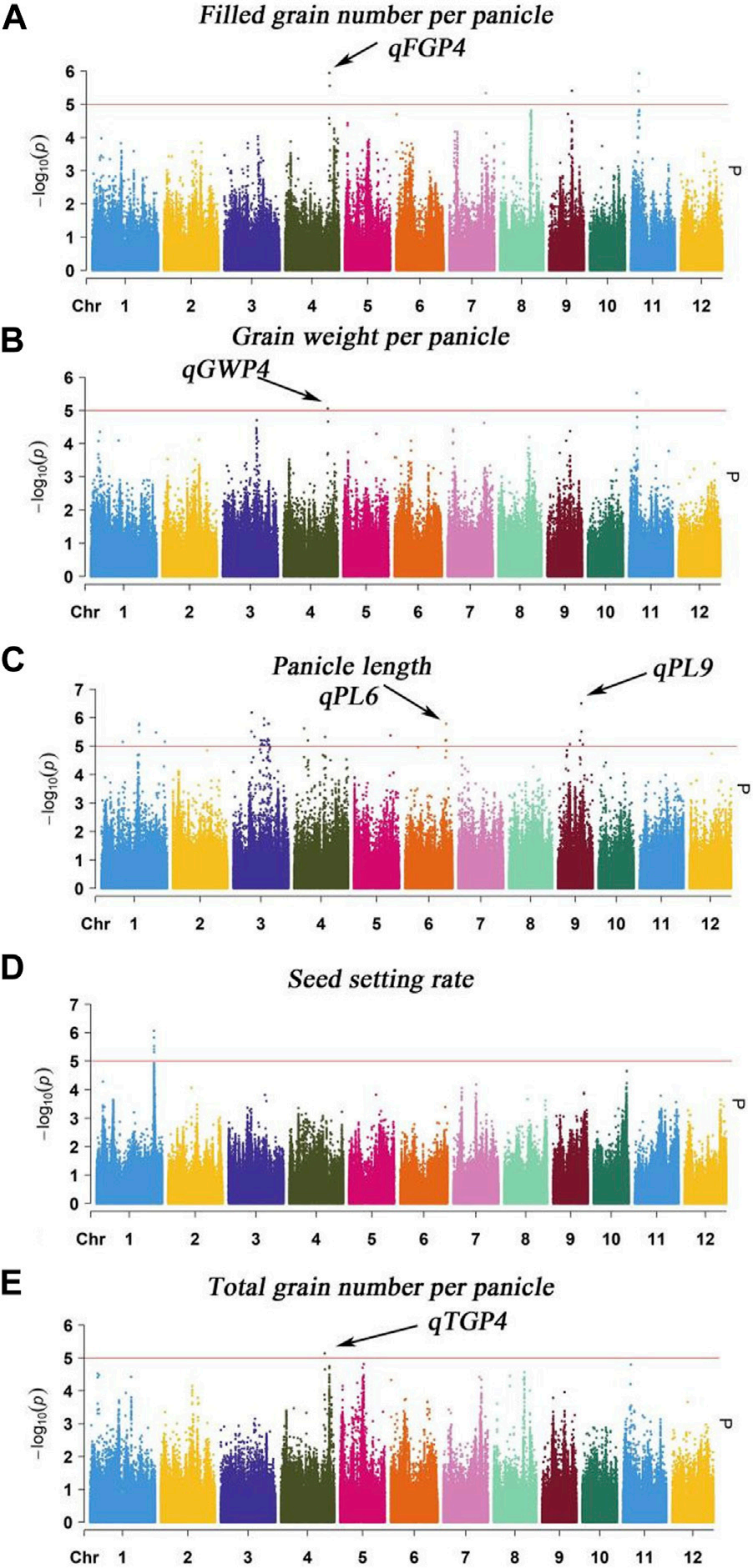
Manhattan plot analysis (MLM) of GWAS for PL, TGP, FGP, SSR and GWP was performed using the genomic BLUP method: **(A)** Manhattan plot for FGP; **(B)** Manhattan plot for GWP; **(C)** Manhattan plot for PL; **(D)** Manhattan plot for SSR; **(E)** Manhattan plot for TGP.

The MLM with correction of kinship bias was mainly used in this study, We conducted a GWAS between panicle traits and SNPs across the 162 rice accessions. The GWAS results showed that a total of 9 SNPs (QTLs) were significantly associated with PL, TGP, FGP, SSR, and GWP (Table 3; Figure 6). For PL, *qPL1*, *qPL6*, *qPL9.1* and *qPL9.2* were identified on chromosomes 1, 6, and 9. For SSR, *qSRR1* was identified on chromosome 1. For TGP, FGP, and GWP, the Chr4_28,917,371 (*qTGP4*, *qFGP4*, and *qGWP4*) locus on chromosome 4 was found to be associated with TGP, FGP, and GWP simultaneously, *qTGP4*, *qFGP4*, and *qGWP4* were the same QTL; hereafter, this QTL is referred to as *qTGP4.* In addition, *qFGP7* was identified on chromosome 7. In summary, the results of single-environment GWAS are consistent with the results of multi-environment GWAS.

**TABLE 3:** Genome-wide significant association of rice panicle traits (BLUP).

Trait	QTLs	Chr	SNP	Allele	BLUP	Model	Reported QTLs and genes
					*p*-value		
PL	*qPL1*	1	25,009,989	A/G	1.6 × 10^−6^	MLM	*LP1* Liu et al. (2016)
	*qPL6*	6	26,935,985	A/G	6.3 × 10^−6^	MLM	
	*qPL9.1*	9	15,393,518	A/C	3.2 × 10^−7^	MLM	
	*qPL9.2*	9	14,388,593	T/C	6.3 × 10^−6^	MLM	
TGP	*qTGP4*	4	28,917,371	C/T	7.2 × 10^−6^	MLM	
FGP	*qFGP4*	4	28,917,371	C/T	1.1 × 10^−6^	MLM	*OsBZR1* Qiao et al. (2017)
	*qFGP7*	7	23,875,734	C/T	4.6 × 10^−6^	MLM	
SSR	*qSSR1.1*	1	37,937,308	G/A	4.8 × 10^−6^	MLM	*sd1* Ye et al. (2015)
GWP	*qGWP4*	4	28,917,371	C/T	8.7 × 10^−6^	MLM	

### 3.5 Identification of candidate genes for *qPL1*

**FIGURE 7:**
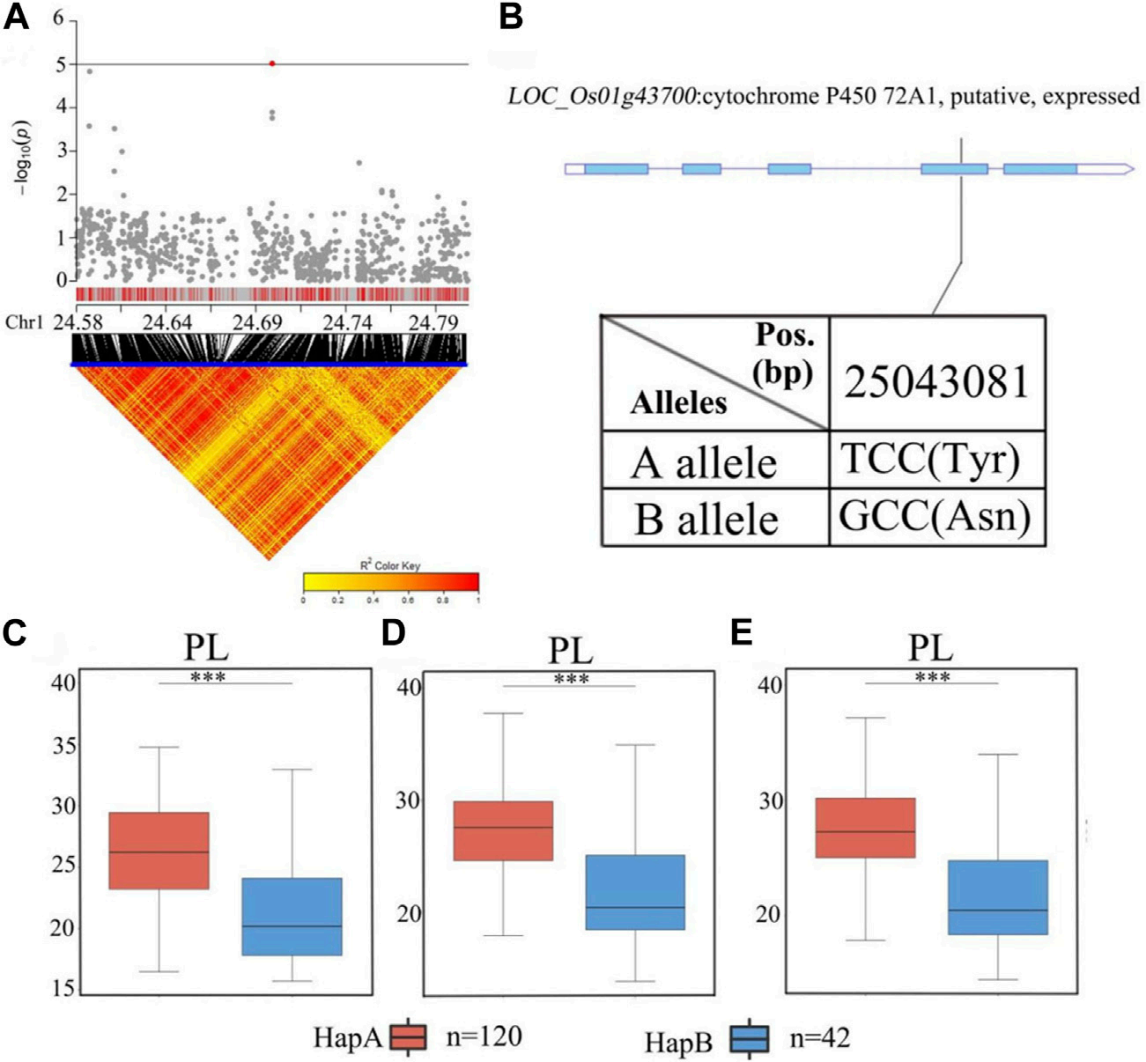
The results of haplotype analysis of candidate genes are as follows: **(A)** Local Manhattan result (top part) and LD heatmap (bottom part); **(B)** The structure diagram of LOC_Os01g43700 and the SNPs between Hap A and Hap B in LOC_Os01g43700 cDNA. The blue fragment represents the exons; **(C)** Box line diagram for the PL trait for the two haplotypes (n = 120 *versus* 42) in 2019; **(D)** Box plots for PL trait for the two haplotypes (n = 120 *versus* 42) in 2020; **(E)** Box plots for PL trait for the two haplotypes (n = 120 *versus* 42) in 2021. The central lines indicate the median values, and the box edges represent the upper and lower quartiles (***, *p* < 0.001, ANOVA).

There were 38 candidate genes associated with significant SNPs in *qPL1* (chr1_24,700,283) in the range of 24.9–25.1 Mb (Figure 7; Supplementary Table S4). In this region, 10 of 38 genes contained nonsynonymous SNPs (Supplementary Table S4). Only one nonsynonymous SNP in *LOC_Os01g43700* was found to be significantly associated with PL. This gene encodes a cytochrome P450 protein. Cytochrome P450 plays vital roles in promoting plant growth and development (Distefano et al., 2021). According to the SNP site in the cDNA sequence of the *LOC_Os01g43700* gene, it was divided into two haplotypes (Figure 7B). The average PL values of 120 accessions carrying the *LOC_Os01g43700* allele were 27.0 ± 4.0 cm. The average PL 42 accessions carrying the allele *LOC_Os01g43700* were 21.7 ± 4.3 cm. Analysis of variance showed that the differences in PL values of the two alleles were highly significant, and the B allele was significantly associated with lower PL than the A allele. (Figures 7C–E). Therefore, *LOC_Os01g43700* may be a candidate gene for PL.

### 3.6 Identification of candidate genes for *qPL9*

**FIGURE 8:**
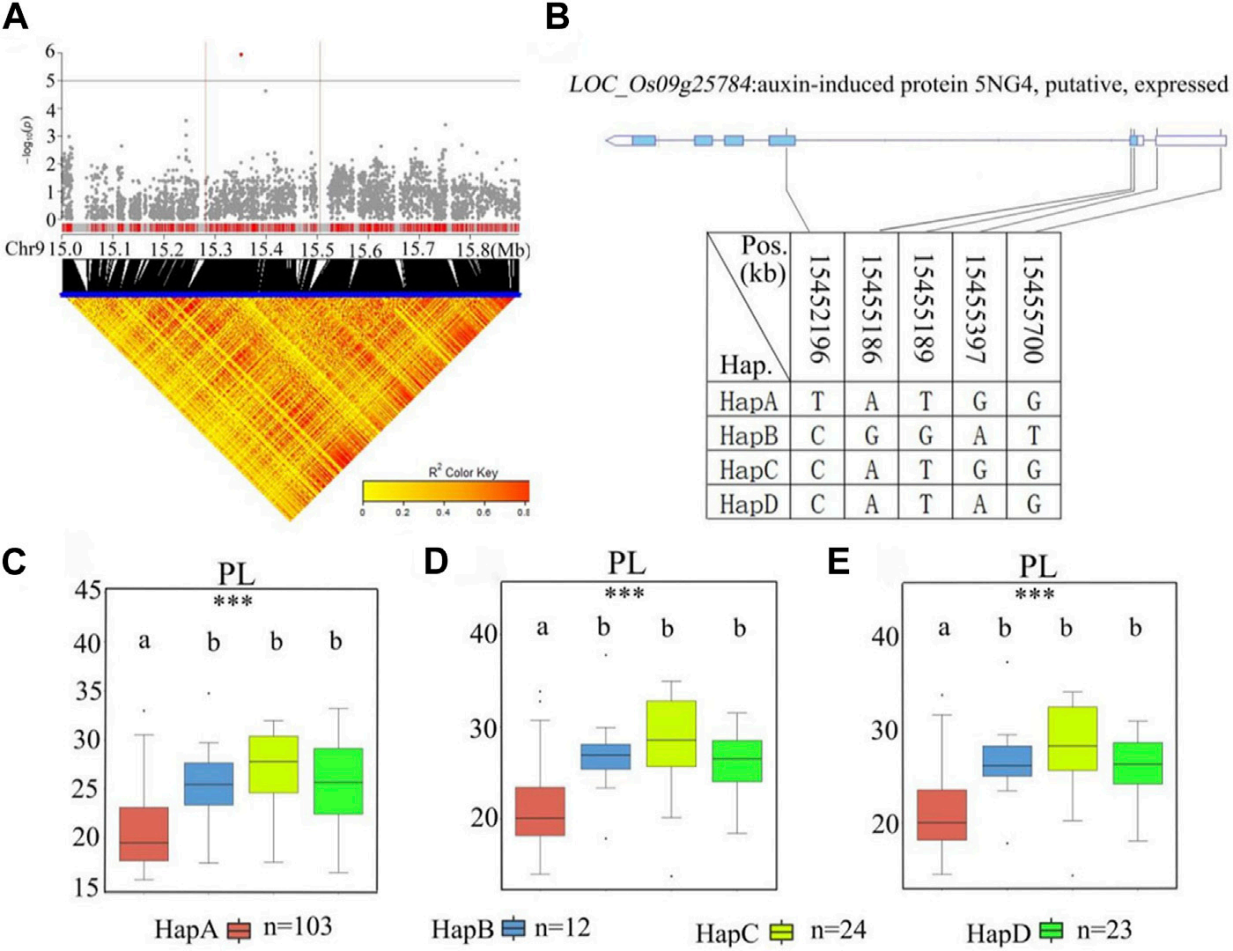
The results of haplotype analysis of candidate genes are as follows: **(A)** Local Manhattan result (top part) and LD heatmap (bottom part); **(B)** The structure diagram of *LOC_Os09g25784* and the SNPs among HapA, HapB, HapC, and HapD in *LOC_Os09g25784* cDNA. The blue fragment represents the exons: **(C)** Box line diagram for the PL trait for the four haplotypes in 2019; **(D)** Box plots for the PL trait for the four haplotypes in 2020; **(E)** Box plots for the PL trait for the four haplotypes in 2021. The central lines indicate the median values, and box edges represent the upper and lower quartiles (***, *p* < 0.001, ANOVA).

A stabilized QTL, *qPL9*, was found to include the previously reported *LP1* QTL that affects PL through a new regulatory pathway (Liu et al., 2016). For *qPL9* (chr9_15,393,518) in the 15.3–15.5 Mb region, there were 20 candidate genes related to remarkable SNPs (Figure 8; Supplementary Table S5). Among the 20 candidate genes, 12 genes had nonsynonymous mutations (Supplementary Table S5). Five nonsynonymous SNPs in *LOC_Os09g25784* were found to be significantly associated with PL. *LOC_Os09g25784* encodes an auxin-induced protein, 5NG4. A previous study demonstrated that auxins are essential molecules that control almost every aspect of plant development (Paque and Weijers, 2016). For *LOC_Os09g25784*, all materials were divided into four haplotypes according to the SNP on the cDNA of this locus (Figure 8B). The average PL values of the four haplotypes were 20.9 ± 3.7 cm, 26.3 ± 4.4 cm, 27.9 ± 4.6 cm, and 26.0 ± 3.6 cm, respectively. Haplotype analysis of the whole population showed that there was no significant difference in PL values among Hap B, Hap C and Hap D, but the PL of Hap B, Hap C and Hap D were significantly longer than Hap A (Figures 8C–E). Among the four haplotypes, HapA had the shortest panicle length. Therefore, we further studied the gene *LOC_Os09g25784*.

### 3.7 Identification of candidate genes for *qTGP4*

**FIGURE 9:**
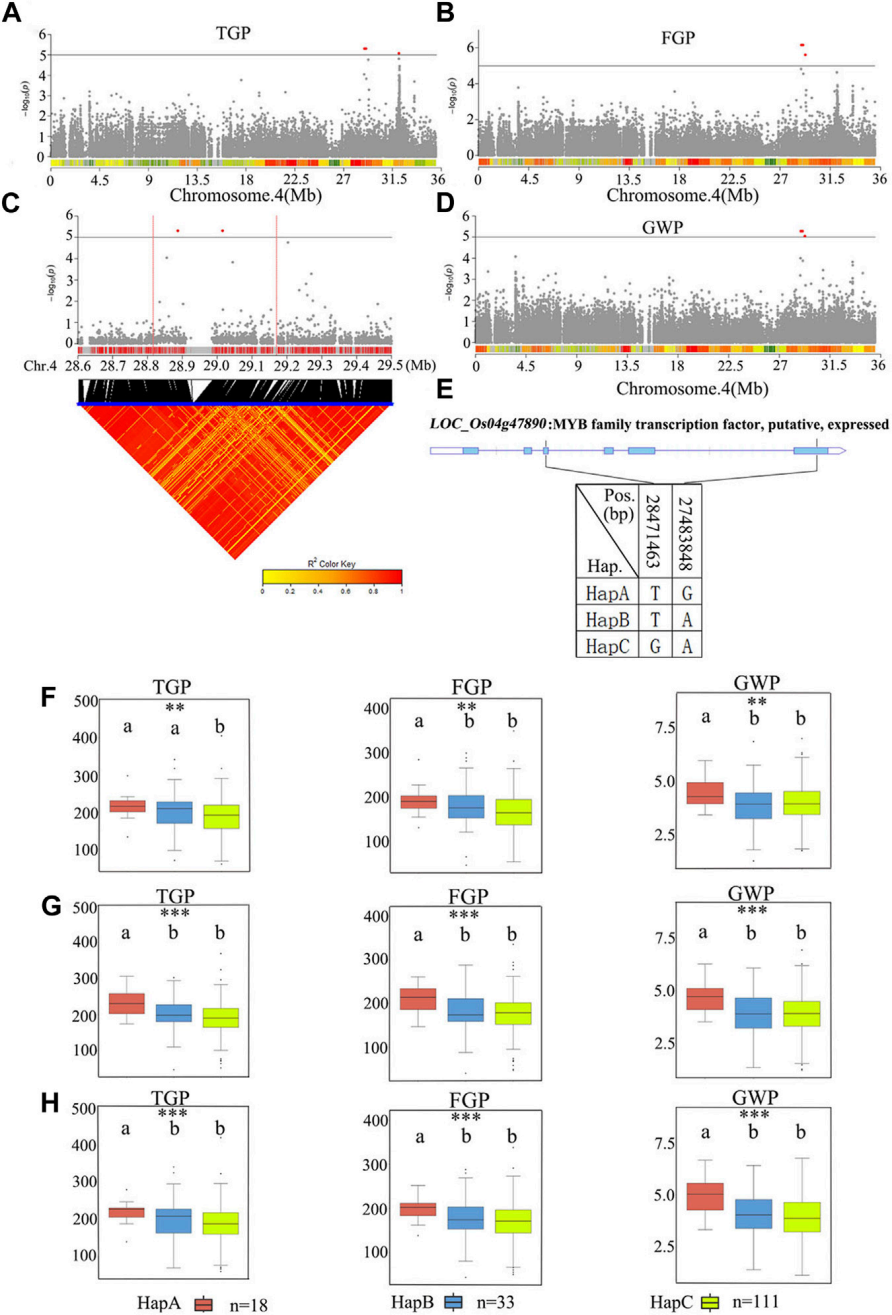
The results of haplotype analysis of candidate genes are as follows: **(A)** Manhattan result chart of TGP trait; **(B)** Manhattan result chart of FGP trait; **(C)** Local Manhattan result (top part) and LD heatmap (bottom part); **(D)** Manhattan plot for GWP. The dashed lines represent significance thresholds; **(E)** The structure diagram of *LOC_Os04g47890* and the SNPs among HapA, HapB and HapC in *LOC_Os04g47890* cDNA. The blue fragment represents the exons. **(F)** Box line diagram for TGP, FGP, and GWP traits for the three haplotypes in 2019; **(G)** Box line diagram for TGP, FGP, and GWP traits for the three haplotypes in 2020; **(H)** Box plots for the TGP, FGP, and GWP traits for the three haplotypes in 2021. The central lines indicate the median values, and the box edges represent the upper and lower quartiles (**, *p* < 0.01; ***, *p* < 0.001, ANOVA).

*qTGP4* was found to be a candidate region for TGP, FGP, and GWP. The 28.8–29.2 Mb regions on chromosome 4 contained 81 genes (Figure 9; Supplementary Table S6). Among the 81 candidate genes, 29 genes had nonsynonymous mutations (Supplementary Table S6). A previous study has shown that the functions of MYB proteins in plants include regulation of secondary metabolism, control of cellular morphogenesis and regulation of meristem formation and the cell cycle (Jin and Martin, 1999). Therefore, *LOC_Os04g47890* (which harbors a gene encoding a MYB family transcription factor) was predicted as a candidate gene QTL. According to the SNP site in the cDNA sequence of the *LOC_Os04g47890* gene, it was divided into three haplotypes (Figure 9E). The average TGP and FGP of the three haplotypes ranged from 187.0 ± 53.2 to 221.0 ± 28.2 and from 170.0 ± 48.4 to 200.0 ± 27.9, respectively. The average GWP ranged from 3.9 ± 1.0 g to 4.8 ± 0.8 g. For TGP, FGP, and GWP, haplotype analysis for populations showed that HapA was associated with significantly greater TGP, FGP, and GWP than HapB and HapC, while there were no significant differences in these traits between HapB and HapC (Figures 9F–H). Therefore, *LOC_Os04g47890* was chosen for further research.

### 3.8 Haplotype distribution of candidate genes

**FIGURE 10:**
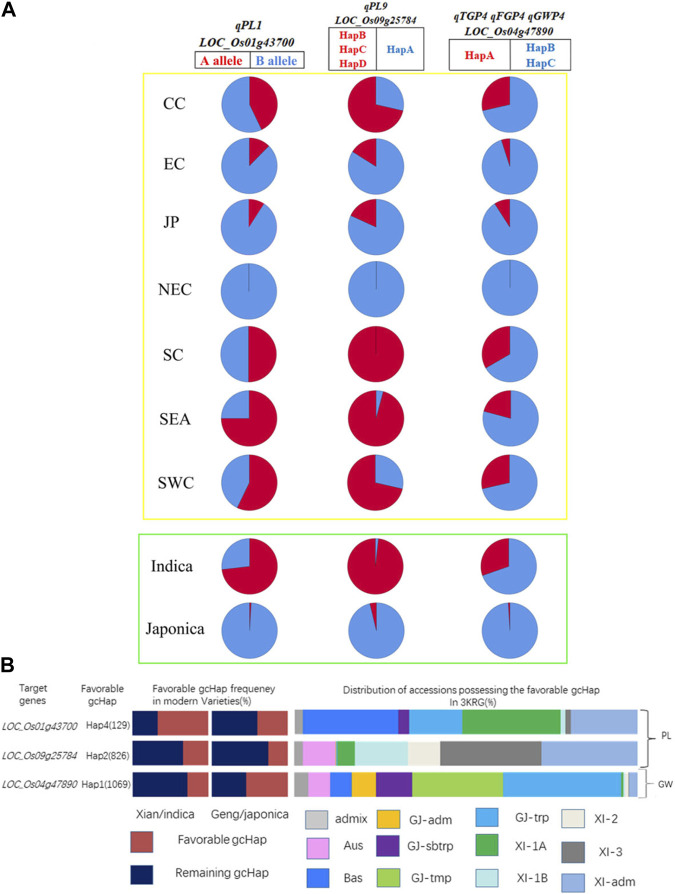
Haplotype distribution of candidate genes. **(A)** We mapped the haplotypes of candidate genes in 2 subgroups and 7 geographical populations. Red and blue were used to represent favorable and unfavorable haplotypes, respectively. The yellow box represents the areas, and the green box represents the subgroups. SC, South China; CC, Central China; EC, East China; NEC, Northeast China; SWC, Southwest China; JP, Japan; SEA, Southeast Asia; **(B)** Frequencies of the favorable gcHap of 3 important candidate genes affecting four panicle traits (PL, TGP, FGP and GWP) in XI and GJ modern varieties and different rice subpopulations.

We summarized the haplotypes of candidate genes based on geographical regions and subgroups (Figure 10A). The favorable haplotypes HapB, HapC and HapD of favorable alleles *LOC_Os01g43700* and *LOC_Os09g25784* were mainly distributed in *indica* rice subgroups such as Southern China (SC), Southwest China (SWC) and Southeast Asia (SEA) and low latitudes. In contrast, the nonfavorable HapB allele of *LOC_Os01g43700* and the nonfavorable HapA allele of *LOC_Os09g25784* were mainly distributed in *japonica* rice subgroups and high latitudes, such as Northeast China (NEC). Similarly, the favorable haplotypes of the three candidate genes were mainly distributed in *indica* rice, and Hap A of *LOC_Os04g47890* was mainly distributed in SC, SWC and SEA. Therefore, germplasms with better PLs, more grains and heavier panicles were mainly distributed in subpopulations of *indica* rice and low-latitude regions. We further analyzed the frequencies of the favorable gcHap of 3 important candidate genes affecting two panicle traits (PL and GW) in Xian/*indica* (XI) and Geng/*japonica* (GJ) modern varieties and different rice subpopulations and the results showed that the GW candidate genes were mainly distributed in *japonica* rice, and the favorable haplotypes of the two PL candidate genes in modern varieties were mainly distributed in *indica* rice. In addition, the favorable haplotypes of 3KRG candidate genes were distributed in different subgroups (Supplementary Table S8). Compared with the three haplotypes carrying the *LOC_Os01g43700* gene from 3,000 rice genome databases, the favorable haplotype carrying the A allele in this study was basically the same with Hap1 (Supplementary Table S8). And compared with the three haplotypes of *LOC_Os09g25784* gene from 3,000 rice genome databases, the favorable haplotypes carrying the B, C, and D alleles had basically the same panicle length with the favorable haplotypes in 3,000 rice genome database (Supplementary Table S8).

### 3.9 Excellent parental combinations predicted for panicle traits

Through further research, we found that the haplotype analysis results of the three genes showed that six haplotypes showed positive effects and the other three haplotypes showed negative effects (Supplementary Table S7). We predicted 10 excellent parents for panicle trait improvement (Table 4). Among the ten predicted excellent parents, nine varieties belonged to *indica* rice, and only one belonged to *japonica* rice. The favorable haplotypes of *LOC_Os01g43700*, *LOC_Os09g25784*, and *LOC_Os04g47890* improved panicle traits. To further verify this hypothesis, we used the *indica* rice variety Yuedao 61 as an example and found that the GWP of the dominant haplotype Hap A containing these three genes could theoretically increase by 0.72 g. Similarly, we also predicted other varieties.

**TABLE 4:** Predicting excellent parents for improving panicle traits.

Best predicted parents	Predicted PL improvement (cm)		Predicted TGP improvement	Predicted FGP improvement	Predicted GWP improvement (g)
	*LOC_Os01g43700*	*LOC_Os09g25784*	*LOC_Os04g47890*		
Baikenuo *(indica)*	HapA (4.03)	HapB (3.30)	HapA (27.00)	HapA (25.00)	HapA (0.72)
Yuedao 61 *(indica)*	HapA (4.03)	HapC (4.83)	HapA (27.00)	HapA (25.00)	HapA (0.72)
Yuedao 109 *(indica)*	HapA (4.03)	HapD (2.92)	HapB (9.00)	HapB (5.00)	HapB (0.02)
Yuedao 62 *(indica)*	HapA (4.03)	HapC (4.83)	HapA (27.00)	HapA (25.00)	HapA (0.72)
Nongxiang 26 *(indica)*	HapA (4.03)	HapC (4.83)	HapA (27.00)	HapA (25.00)	HapA (0.72)
Yuedao 48 *(indica)*	HapA (4.03)	HapC (4.83)	HapB (9.00)	HapB (5.00)	HapB (0.02)
Zhengdao 10 Hao *(indica)*	HapA (4.03)	HapB (3.30)	HapB (9.00)	HapB (5.00)	HapB (0.02)
Chuan 5 Xian *(indica)*	HapA (4.03)	HapB (3.30)	HapA (27.00)	HapA (25.00)	HapA (0.72)
Qimiaoxiang 2 Hao *(indica)*	HapA (4.03)	HapD (2.92)	HapB (9.00)	HapB (5.00)	HapB (0.02)
Shengtangqing *(Japonica)*	HapA (4.03)	HapD (2.92)	HapA (27.00)	HapA (25.00)	HapA (0.72)

## 4 Discussion

Panicle traits are complex quantitative traits regulated by multiple genes and controlled by the environment (Bai et al., 2021). Here, we observed and collated the phenotypic data of five panicle traits of 162 rice materials, and identified abundant phenotypic variations. Although only 162 rice germplasm resources were used in this study, these germplasm resources have a wide range of sources, including germplasm resources from China and four other Asian countries. Furthermore, the 162 materials in this study have abundant genetic and phenotypic variations, which are suitable for association analysis. The phenotypic variation of PL was high, and the coefficient of variation (CV) was between 20.9% and 21.2%, which was similar to other studies. For instance, the PL phenotype of 340 rice materials from 3,000 rice genome projects was identified, and the coefficient of variation was between 15% and 16% (Bai et al., 2021). Liu et al. used three mapping populations for PL phenotypic identification and found that the CVs ranged from 15.3% to 19.6% (Liu et al., 2016). In addition, we calculated the Pearson correlation coefficient, and the results showed that among 162 rice materials, TGP, FGP and GWP had similar positive correlation trends (Figure 3), which is consistent with the GWAS results; that is, some QTLs can be detected by two models within 2 years or related to multiple traits (Supplementary Table S2).

**FIGURE 11:**
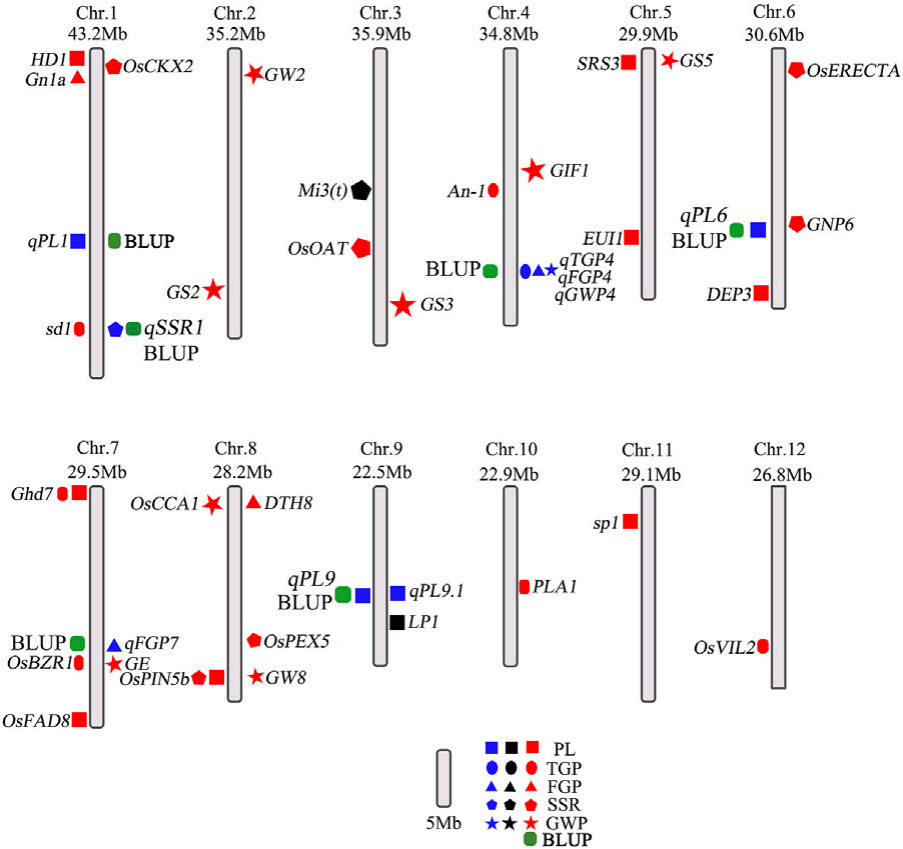
Distribution of QTLs and genes related to panicle traits on rice chromosomes. The blue colors represent QTLs in this study; the black and red colors represent QTLs and genes in published studies, respectively. The green represents the QTLs co-located by BLUP method.

Genome-wide association analysis (GWAS) is a powerful method to understand the genetic structure of multiple traits in rice. Generally, regression models are constructed to test whether there is a correlation between markers and phenotypes. Previous studies have shown that MLMs control false positives better than GLMs do, which was strongly reflected by experimental results. Therefore, we used the MLM and GLM for further analysis. In this study, a total of 14 QTLs related to panicle traits were detected, and 9 QTLs were detected in at least 2 years and via two models. The local LD regions of the four QTLs associated with panicle traits overlapped with the flanking regions of the two genes (*OsBZR1* and *sd1*) among the 9 stable QTLs and one QTL (*LP1*) reported previously (Ye et al., 2015; Liu et al., 2016; Qiao et al., 2017). The remaining 6 QTLs were all newly detected (Table 2; Supplementary Figure S1), and the results of the genomic BLUP method were completely consistent (Figure 6), indicating that our results were highly reliable. *LP1* colocalized with *qPL9* and *PL9.1*, which was delimited to a 90 kb region for the first time (Liu et al., 2016). *OsBZR1* colocalized with *qFGP7*, which is involved in plant growth and development. Overexpression of *OsBZR1* results in higher sugar accumulation in developing anthers and seeds and increased seed yield (Qiao et al., 2017). *Sd1*, near *qSSR1*, encodes a gibberellin 20 oxidase enzyme participating in gibberellic acid biosynthesis and is involved in the control of the grain number and SSR of rice (Su et al., 2021). The QTL *qFGP7* was near the cloned gene *GE*, which encodes a CYP78A subfamily P450 monooxygenase (Yang et al., 2013). Therefore, a total of 6 QTLs are novel QTLs reported in our study (Figure 11).

Haplotype analysis of *LOC_Os01g43700* in the significant locus qPL1 revealed two haplotypes (Figure 7B). In the whole population, haplotypes affected the average PL, and HapA guided greater PL (Figure 7C), indicating that *LOC_Os01g43700* may affect PL variation. The difference in haplotype frequency between the two subspecies indicated that the gene *LOC_Os01g43700* was selected in the rice breeding process, and HapA was contained in 70% of *indica* rice materials (Figure 10). In addition, the gene *LOC_Os01g43700* encodes a cytochrome P450 protein. Previous studies have shown that cytochrome P450 plays an important role in plant growth and development (Pandian et al., 2020; Distefano et al., 2021) and is involved in biosynthetic pathways and detoxification pathways (Xu et al., 2015). The results demonstrated that the gene *LOC_Os01g43700* may be a candidate gene for PL variation. *qPL9* (*LOC_Os09g25784*), which was found to be associated with PL, was detected in all 3 years in this study and this gene was selected as the focus for further analysis. *LOC_Os09g25784* encodes the auxin-induced protein 5NG4, and auxin is an essential molecule that controls almost every aspect of plant development (Paque and Weijers, 2016). Analysis of *LOC_Os09g25784* revealed four haplotypes (Figure 8B). Significant differences in mean PL were found between accessions with different haplotypes throughout the whole panel, with HapB, HapC, and HapD governing the longer panicles, indicating that the gene *LOC_Os09g25784* may be responsible for PL variation. *qTGP4*, *qFGP4*, and *qGWP4* were in the same QTL, and were found to be associated with TGP, FGP, and GWP in all 3 years. Based on the results of functional annotation and haplotype analysis of candidate genes, we proposed that *LOC_Os04g47890* (which encodes a MYB family transcription factor) is a candidate gene in *qTGP4*, *qFGP4*, and *qGWP4*. The MYB transcription factor family is present in all eukaryotes. Compared with other organisms, plants encode many MYB genes (Kranz et al., 2000; Qu and Zhu, 2006; Yanhui et al., 2006). MYB proteins compose a superfamily of transcription factors that play regulatory roles in developmental processes in plants (Yanhui et al., 2006; Wang et al., 2020). Consequently, *LOC_Os04g47890* may be a candidate gene for regulating rice panicle traits.

The results showed that favorable haplotypes could improve the panicle traits of rice. Among the 10 predicted parents, 9 *indica* rice improved panicle traits better than *indica* rice, and these results need to be further verified in production. Overall, further functional analysis of these candidate genes will help us better understand the genetic basis for natural variation in rice panicle traits and show that these genes may be used to improve rice yield. With the development of molecular biology, our research on the mining of excellent alleles of rice panicle traits and the prediction of excellent parents will provide a theoretical basis for molecular design breeding. Therefore, these candidate genes are to be used in breeding programme not only the breeding program discussed here but also other breeding programs.

## 5 Conclusion

In our study, we dissected the genetic basis underlying rice panicle traits. According to the results of gene function annotation and haplotype analysis, we identified three candidate genes related to panicle traits. *LOC_Os01g43700* and *LOC_Os09g25784* were identified as candidate genes for PL, while *LOC_Os04g47890* was identified as a candidate gene for TGP, FGP, and GWP. These genes are important for further research. At the same time, we infer that these 10 rice materials can be used as excellent parents with favorable alleles of panicle trait genes. Using the favorable alleles detected in this study can provide a theoretical basis for the improvement and breeding of panicle traits.

## Data Availability

The data presented in the study are deposited in the NCBI Sequence Read Archive repository, accession number PRJNA554986.
