# Supplementary material for: Mining the candidate genes of rice panicle traits via a genome-wide association study
Source: Front Genet. 2023 Sep 4;14:1239550. doi: 10.3389/fgene.2023.1239550 (PMC10507276; doi:10.3389/fgene.2023.1239550)
Supplement: Supplementary file 1 [file Table1.DOCX]

Supplementary Material

Mining the Candidate Genes of Rice Panicle Traits Via a Genome-Wide Association Study

Zhengbo Liu^1†^, Hao Sun^†^, Yanan Zhang^†^, Mingyu Du^†^, Jun Xiang, Xinru Li, Yinping Chang, Jinghan Sun, Xianping Cheng, Mengyuan Xiong, Zhe Zhao and Erbao Liu^1*^

^1^College of Agronomy, Anhui Agricultural University, Hefei 230000, China

***Correspondence**: liuerbao@ahau.edu.cn (E.L.)

† These authors contributed equally to this work.

# Supplementary Figures and Tables

## Supplementary Tables

**Supplementary Table 1**

Table S1. Names and origins of 162 rice accessions used for association mapping.

| Code | Germplasm name | Origin | Latitude | Longitude | Germplasm ID^a^ | Subpopulation | region |
| --- | --- | --- | --- | --- | --- | --- | --- |
|  |  |  |  |  |  |  |  |
| 1 | Longjing 22 | Haerbin, Heilongjiang, China | 44.0 | 125.4 | HS2008010 | Japonica | NEC |
| 2 | Longjing 28 | Haerbin, Heilongjiang, China | 44.0 | 125.4 | HS2009011 | Japonica | NEC |
| 3 | Longjing 27 | Haerbin, Heilongjiang, China | 44.0 | 125.4 | HS2009010 | Japonica | NEC |
| 4 | 24248 | Nanjing, Jiangsu, China | 32.0 | 118.8 |  | Japonica | EC |
| 5 | Tijin | Japan | 35.7 | 139.7 | H1706 | Japonica | JP |
| 6 | Zhongguo 91 | Japan | 35.7 | 139.7 | NL274 | Japonica | JP |
| 7 | Kangbingyueguang | Japan | 35.7 | 139.7 | H1524 | Japonica | JP |
| 8 | Sihao 4385 | Sihong, Jiangsu, China | 33.5 | 118.2 | H1315 | Japonica | EC |
| 9 | Nannongjing 1R | Nanjing, Jiangsu, China | 32.0 | 118.8 | H1372 | Japonica | EC |
| 10 | Hongmangshajing | Kunshan, Jiangsu, China | 31.4 | 121.0 | T630 | Indica | EC |
| 11 | Wanhuangdao | Wuxian,Jiangsu,China | 31.3 | 120.6 | T815 | Japonica | EC |
| 12 | Xudao 3hao | Haerbin, Heilongjiang, China | 34.3 | 117.2 | SS200306 | Japonica | NEC |
| 13 | Youzhiyueguang | Japan | 35.7 | 139.7 | Y1A01876 | Japonica | JP |
| 14 | Yuedao 68 | Vietnam | 10.2 | 106.0 | Y1A02418 | Indica | SEA |
| 15 | Longdao 8hao | Haerbin, Heilongjiang, China | 44.0 | 125.4 | HS2008019 | Japonica | NEC |
| 16 | Longdao 6hao | Haerbin, Heilongjiang, China | 44.0 | 125.4 | HS2006004 | Japonica | NEC |
| 17 | Qiutianxiaoding | Japan | 35.7 | 139.7 | H1654 | Japonica | JP |
| 18 | Zhenghan 2hao | Zhengzhou, Henan, China | 34.8 | 113.7 | GS2003031 | Japonica | CC |
| 19 | Xiangjing 9407 | Nanjing, Jiangsu, China | 32.0 | 118.8 | LS891061 | Japonica | EC |
| 20 | Nongxiang 21 | Changsha, Hunan, China | 28.2 | 113.0 | CNA200802496 | Indica | CC |
| 21 | Fengyouwan 8hao | Changsha, Hunan, China | 28.2 | 113.0 | YS2009001 | Indica | CC |
| 22 | Xiangwanxian 17 | Changsha, Hunan, China | 28.2 | 113.0 | XS2008035 | Indica | CC |
| 23 | Yuedao 37 | Vietnam | 10.2 | 106.0 | Y1A02397 | Indica | SEA |
| 24 | Sujing 353 | Suzhou, Jiangsu, China | 31.3 | 120.6 | C1511 | Japonica | EC |
| 25 | Zhen9424 | Zhenjiang, Jiangsu, China | 32.1 | 119.3 | ZD-05658 | Japonica | EC |
| 26 | Baikenuo | Wujiang, Jiangsu, China | 31.2 | 120.6 | T354 | Indica | EC |
| 27 | Diantun502xuanzao | Kunming, Yunnan, China | 25.0 | 102.7 | ZD-05551 | Indica | SWC |
| 28 | Yuedao 41 | Vietnam | 10.2 | 106.0 | Y1A02328 | Indica | SEA |
| 29 | Longnuo 3hao | Haerbin, Heilongjiang, China | 44.0 | 125.4 | HS2009015 | Japonica | NEC |
| 30 | Mudanjiang 28 | Mudanjiang, Heilongjiang, China | 44.6 | 129.6 | HS2006006 | Japonica | NEC |
| 31 | Yujing 6hao | Zhengzhou, Henan, China | 34.8 | 113.7 | GS980002 | Japonica | CC |
| 32 | Shengdao808 | Haerbin, Heilongjiang, China | 44.0 | 125.4 |  | Japonica | NEC |
| 33 | Yuedao 32 | Vietnam | 10.2 | 106.0 | Y1A02326 | Indica | SEA |
| 34 | Yuedao 107 | Vietnam | 10.2 | 106.0 | Y1A02368 | Indica | SEA |
| 35 | Yuedao 61 | Vietnam | 10.2 | 106.0 | Y1A02413 | Indica | SEA |
| 36 | Yuedao 50 | Vietnam | 10.2 | 106.0 | Y1A02331 | Indica | SEA |
| 37 | Yuedao 109 | Vietnam | 10.2 | 106.0 | Y1A02356 | Indica | SEA |
| 38 | Yuedao 62 | Vietnam | 10.2 | 106.0 | Y1A02414 | Indica | SEA |
| 39 | Yuedao 66 | Vietnam | 10.2 | 106.0 | Y1A02417 | Indica | SEA |
| 40 | Hongnong 5hao | Wujiang, Jiangsu, China | 31.2 | 120.6 | T757 | Japonica | EC |
| 41 | Suyunuo | xuzhou, Jiangsu, China | 34.3 | 117.2 | T832 | Japonica | EC |
| 42 | Shenlenuo | Kunshan, Jiangsu, China | 31.4 | 121.0 | T691 | Japonica | EC |
| 43 | Hongjiaozhan | Wuxian, Jiangsu, China | 31.3 | 120.6 | T888 | Indica | EC |
| 44 | Wanjingnuo | Hefei, Anhui, China | 31.9 | 117.3 | H1653 | Japonica | EC |
| 45 | Nongxiang26 | Changsha, Hunan, China | 28.2 | 113.0 |  | Indica | CC |
| 46 | Yuedao 9 | Vietnam | 10.2 | 106.0 | Y1A02373 | Indica | SEA |
| 47 | M1004 | Japan | 35.7 | 139.7 | Y1A01861 | Japonica | JP |
| 48 | Nongxiang 25 | Changsha, Hunan, China | 28.2 | 113.0 | GS2001021 | Indica | CC |
| 49 | Longjing 20 | Haerbin, Heilongjiang, China | 44.0 | 125.4 | HS2007004 | Japonica | NEC |
| 50 | Xiangchuanwuxinbaimi | Japan | 35.7 | 139.7 | H1655 | Japonica | JP |
| 51 | Jindao 1007 | Dongli, Tianjin, China | 39.1 | 117.1 | GS2004043 | Japonica | EC |
| 52 | Zaijinjing | Songhuajiang, Heilongjiang, China | 41.4 | 119.5 | H1614 | Japonica | NEC |
| 53 | Malaihong | Nanjing, Jiangsu, China | 32.0 | 118.8 | T050 | Japonica | EC |
| 54 | Nannongjing3786 | Haerbin, Heilongjiang, China | 44.0 | 125.4 |  | Japonica | NEC |
| 55 | Wuyunjing 8hao | Wujin, Jiangsu, China | 31.8 | 120.0 | SZS313 | Japonica | EC |
| 56 | Yuzhenxiang | Changsha, Hunan, China | 28.2 | 113.0 | XS2009038 | Indica | CC |
| 57 | Yuedao24(LCV18) | Vietnam | 10.2 | 106.0 |  | Indica | SEA |
| 58 | Yuedao 3 | Vietnam | 10.2 | 106.0 | Y1A02370 | Indica | SEA |
| 59 | Yuedao 43 | Vietnam | 10.2 | 106.0 | Y1A02404 | Indica | SEA |
| 60 | Yuedao 48 | Vietnam | 10.2 | 106.0 | Y1A02407 | Indica | SEA |
| 61 | Yuedao 49 | Vietnam | 10.2 | 106.0 | Y1A02408 | Indica | SEA |
| 62 | Yuedao 13 | Vietnam | 10.2 | 106.0 | Y1A02320 | Indica | SEA |
| 63 | Wumangyedao | Jinshan, Shanghai, China | 30.8 | 121.3 | T335 | Japonica | EC |
| 64 | Haobuqia | Wuxian, Jiangsu, China | 31.3 | 120.6 | 21-00357 | Indica | EC |
| 65 | Yuedao 22 | Vietnam | 10.2 | 106.0 | Y1A02382 | Indica | SEA |
| 66 | Dongzhengwuyunjing 21 | Hongze, Jiangsu, China | 33.3 | 118.9 | SS200705-1 | Japonica | EC |
| 67 | Yandao 8hao | Yancheng, Jiangsu, China | 33.4 | 120.1 | SS200307 | Japonica | EC |
| 68 | Huaidao 11hao | Huaian, Jiangsu, China | 33.5 | 119.2 | SS200805 | Japonica | EC |
| 69 | Nannongjing 004 | Nanjing, Jiangsu, China | 32.0 | 118.8 | T248 | Japonica | EC |
| 70 | Zhongzuo 93 | Mudanjiang, Heilongjiang, China | 44.6 | 129.6 | JS1995001 | Japonica | NEC |
| 71 | Xudao 5hao | Xuzhou, Jiangsu, China | 34.2 | 117.1 | GS2006059 | Japonica | EC |
| 72 | Huaidao 8hao | Huaian, Jiangsu, China | 33.5 | 119.2 | SS200410 | Japonica | EC |
| 73 | Dongzheng 1640 | Hongze, Jiangsu, China | 33.3 | 118.9 | C1515 | Japonica | EC |
| 74 | Yanjing 8hao | Yancheng, Jiangsu, China | 33.4 | 120.1 | ZD-05649 | Japonica | EC |
| 75 | Huifeng 2 | Yancheng, Jiangsu, China | 33.4 | 120.1 | C1509 | Japonica | EC |
| 76 | Yandao 9hao | Yancheng, Jiangsu, China | 33.4 | 120.1 | SS200506 | Japonica | EC |
| 77 | Lianjing 4hao | Lianyungang, Jiangsu, China | 34.6 | 119.2 | SS200704 | Japonica | EC |
| 78 | Huifeng 1 | Yancheng, Jiangsu, China | 33.4 | 120.1 | C1508 | Japonica | EC |
| 79 | Sihao 4280 | Sihong, Jiangsu, China | 33.5 | 118.2 | H1705 | Japonica | EC |
| 80 | Sihao 4330 | Sihong, Jiangsu, China | 33.5 | 118.2 | H1704 | Japonica | EC |
| 81 | Sihao 4259 | Sihong, Jiangsu, China | 33.5 | 118.2 | H1334 | Japonica | EC |
| 82 | Zhengdao 18 | Zhengzhou, Henan, China | 34.8 | 113.7 | GS2007033 | Japonica | CC |
| 83 | Jingnuo 330 | Hefei, Anhui, China | 31.9 | 117.3 | H1346 | Japonica | EC |
| 84 | Zhongjing 212 | Nanjing, Jiangsu, China | 32.0 | 118.8 | WS891061 | Japonica | EC |
| 85 | Wuyunjing 21hao | Wujin, Jiangsu, China | 31.8 | 120.0 | SS200705-2 | Japonica | EC |
| 86 | Huajing 6hao | Huaian, Jiangsu, China | 33.5 | 119.2 | SS200706 | Japonica | EC |
| 87 | Yangfujing 8hao | Yancheng, Jiangsu, China | 33.4 | 120.1 | SS200608 | Japonica | EC |
| 88 | Zhendao 99 | Zhenjiang, Jiangsu, China | 32.1 | 119.3 | SS200106 | Japonica | EC |
| 89 | Ningjing 2hao | Nanjing, Jiangsu, China | 32.0 | 118.8 | WPS05010476 | Japonica | EC |
| 90 | Huajing 5hao | Huaian, Jiangsu, China | 33.5 | 119.2 | SS200505 | Japonica | EC |
| 91 | Zhongjing 9677 | Nanjing, Jiangsu, China | 32.0 | 118.8 | C1512 | Japonica | EC |
| 92 | Yangfujing 7hao | Yangzhou, Jiangsu, China | 32.2 | 119.3 | SS200413 | Japonica | EC |
| 93 | Yuedao 12 | Vietnam | 10.2 | 106.0 | Y1A02375 | Indica | SEA |
| 94 | Jianongnuo 2hao | Haerbin, Heilongjiang, China | 44.0 | 125.4 | H1600 | Japonica | NEC |
| 95 | Xudao 4hao | Xuzhou, Jiangsu, China | 34.2 | 117.1 | CNA20040007.X | Japonica | EC |
| 96 | Sihao 4040 | Sihong, Jiangsu, China | 33.5 | 118.2 | H1703 | Japonica | EC |
| 97 | Yanjing 9hao | Yancheng, Jiangsu, China | 33.4 | 120.1 | SS200707 | Japonica | EC |
| 98 | Sihao 4081 | Sihong, Jiangsu, China | 33.5 | 118.2 | H1337 | Japonica | EC |
| 99 | Sihao 4041 | Sihong, Jiangsu, China | 33.5 | 118.2 | H1338 | Japonica | EC |
| 100 | Sihao 4031 | Sihong, Jiangsu, China | 33.5 | 118.2 | H1340 | Japonica | EC |
| 101 | Wandao 68 | Hefei, Anhui, China | 31.9 | 117.3 | WPS03010384 | Japonica | EC |
| 102 | Wuxiang99-8 | Suihua, Heilongjiang, China | 46.6 | 127.0 |  | Japonica | NEC |
| 103 | Zhongjing 131 | Haerbin, Heilongjiang, China | 44.0 | 125.4 | H1620 | Japonica | NEC |
| 104 | Ningjinghui 260 | Nanjing, Jiangsu, China | 32.0 | 118.8 | H1371 | Japonica | EC |
| 105 | Ningjinghui 237 | Nanjing, Jiangsu, China | 32.0 | 118.8 | H1374 | Japonica | EC |
| 106 | Wunuoyihao | Haerbin, Heilongjiang, China | 44.0 | 125.4 | H1611 | Japonica | NEC |
| 107 | Sihao 4029 | Sihong, Jiangsu, China | 33.5 | 118.2 | H1333 | Japonica | EC |
| 108 | Wanqu 429bp | Haerbin, Heilongjiang, China | 44.0 | 125.4 | HS2013003 | Japonica | NEC |
| 109 | Yangfujing 4901 | Yangzhou, Jiangsu, China | 32.2 | 119.3 | SS200811 | Japonica | EC |
| 110 | Yandao 6hao | Yancheng, Jiangsu, China | 33.4 | 120.1 | SS200205 | Japonica | EC |
| 111 | Cbao | Hefei, Anhui, China | 31.9 | 117.3 | H1661 | Japonica | EC |
| 112 | Zhengdao 10hao | Zhenjiang, Jiangsu, China | 32.2 | 119.4 | SS200710 | Indica | EC |
| 113 | Baoxintaihuqing | Wujiang, Jiangsu, China | 31.2 | 120.6 | T834 | Japonica | EC |
| 114 | Huaidao 9hao | Huaian, Jiangsu, China | 33.5 | 119.2 | SS200607 | Japonica | EC |
| 115 | Xiaobaidao | Wuxian, Jiangsu, China | 31.3 | 120.6 | T208 | Japonica | EC |
| 116 | Yaxuenuo | Wuxian, Jiangsu, China | 31.3 | 121.6 | T480 | Japonica | EC |
| 117 | Yangguang 200 | Xuzhou, Jiangsu, China | 34.2 | 117.1 | GS2008043 | Japonica | EC |
| 118 | Zaoshirihuangdao | Wuxian, Jiangsu, China | 31.3 | 120.6 | T728 | Japonica | EC |
| 119 | Luohanhuang | Jiangyin, Jiangsu, China | 31.9 | 120.3 | T560 | Japonica | EC |
| 120 | Xudao2hao | Xuzhou, Jiangsu, China | 34.2 | 117.1 |  | Japonica | EC |
| 121 | Xudao9201B | Xuzhou, Jiangsu, China | 34.2 | 117.1 |  | Japonica | EC |
| 122 | Ebusinuodao | Suihua, Heilongjiang, China | 46.6 | 127.0 | T386 | Japonica | NEC |
| 123 | Yueguang | Japan | 35.7 | 139.7 | H1660 | Japonica | JP |
| 124 | Yimuhu | Japan | 35.7 | 139.7 | Y1A01857 | Indica | JP |
| 125 | Qingkong | Nanjing, Jiangsu, China | 32.0 | 118.8 | Y1A01858 | Japonica | EC |
| 126 | RT61 | Japan | 35.7 | 139.7 | Y1A01863 | Japonica | JP |
| 127 | IL38 | Japan | 35.7 | 139.7 | ZD-05554 | Japonica | JP |
| 128 | Liuyezhan | Hubei, China | 30.0 | 114.0 | 17-00524 | Indica | CC |
| 129 | Zaoxian 14 | Anhui, China | 37.5 | 117.2 | 11-00670 | Indica | EC |
| 130 | Xu91075 | Xuzhou, Jiangsu, China | 34.3 | 117.2 | H1418 | Indica | EC |
| 131 | Xudao 25-7 | Xuzhou, Jiangsu, China | 34.3 | 117.2 | H1419 | Japonica | EC |
| 132 | Qing 7 | Yancheng, Jiangsu, China | 33.4 | 120.1 | H1471 | Indica | EC |
| 133 | Sihao 4141 | Sihong, Jiangsu, China | 33.5 | 118.2 | H1332 | Japonica | EC |
| 134 | Suwujing | Wujin, Jiangsu, China | 31.8 | 120.0 | SS201009 | Indica | EC |
| 135 | 9522B | Changzhou, Jiangsu, China | 31.8 | 120.0 | GS20000008 | Japonica | EC |
| 136 | 863B | Nanjing, Jiangsu, China | 32.0 | 118.8 | H1425 | Japonica | EC |
| 137 | A7444 | Nanjing, Jiangsu, China | 32.0 | 118.8 | H1476 | Japonica | EC |
| 138 | Xiepihuang | Taicang, Jiangsu, China | 31.5 | 121.1 | T203 | Japonica | EC |
| 139 | Shengtangqing | Changshu, Jiangsu, China | 31.6 | 120.7 | T759 | Japonica | EC |
| 140 | Chuan 6xian | Chengdu, Sichuan, China | 30.7 | 104.1 | H1506 | Indica | SWC |
| 141 | Chuan 5xian | Chengdu, Sichuan, China | 30.7 | 104.1 | H1507 | Indica | SWC |
| 142 | Shufeng 101 | Sichuan, China | 30.4 | 104.1 | ZD-00760 | Indica | SWC |
| 143 | Chengnongshuijing | Sichuan, China | 30.4 | 104.1 | ZD-03386 | Indica | SWC |
| 144 | Xiangxiandao 10hao | Changsha, Hunan, China | 28.2 | 113.0 | H1486 | Indica | CC |
| 145 | Ⅱ-32B | Changsha, Hunan, China | 28.2 | 113.0 | A0050 | Indica | CC |
| 146 | Chenwan 3hao | Hunan, China | 28.1 | 113.0 | ZD-00358 | Indica | CC |
| 147 | Xiangaizao 10hao | Hunan, China | 28.1 | 113.0 | ZD-01402 | Indica | CC |
| 148 | Yuetai B | Fogang, Guangdong, China | 23.9 | 113.5 | H1493 | Indica | SC |
| 149 | Qimiaoxiang 2hao | Qingyuan, Guangdong, China | 23.7 | 113.0 | H1496 | Indica | SC |
| 150 | Shengyou 2hao | Gaoyao, Guangdong, China | 23.1 | 112.4 | YS1994004 | Indica | SC |
| 151 | Guichao 2hao | Guangdong, China | 23.1 | 113.2 | H1645 | Indica | SC |
| 152 | LongtepuB | Fuzhou, Fujian, China | 26.1 | 119.3 | H1490 | Indica | EC |
| 153 | Hainanxian R | Hainan, China | 19.5 | 109.6 | H1504 | Indica | SC |
| 154 | Zajiaohaigu | Changjiang, Hainan, China | 19.3 | 109.0 | H1510 | Indica | SC |
| 155 | Nuohangu | Kunming, Yunnan, China | 25.0 | 102.7 | H1434 | Japonica | SWC |
| 156 | Lincangwazuhangu | Kunming, Yunnan, China | 25.0 | 102.7 | H1435 | Japonica | SWC |
| 157 | Yuedao 55 | Vietnam | 10.2 | 106.0 | Y1A02409 | Indica | SEA |
| 158 | Yuedao 108 | Vietnam | 10.2 | 106.0 | Y1A02355 | Indica | SEA |
| 159 | IR112 | Philippines | 14.6 | 121.0 | H1501 | Indica | SEA |
| 160 | IR64 | Philippines | 14.6 | 121.0 | H1502 | Indica | SEA |
| 161 | Gendjah Gempol | Indonesia | 6.1 | 94.5 | 12483 | Japonica | SEA |
| 162 | Shengdao 14 | Jinan, Shandong, China | 36.4 | 117.0 | H1701 | Japonica | EC |

The word in blue type indicated that these materials were the approved variety; CNA indicated the No. of variety right. GS, the abbreviation of guoshendao. HS, the abbreviation of heishendao. These indicated that the accessions were approved by Heilongjiang province. JS, the abbreviation of jishendao. These indicated that the accessions were approved by Jilin province. LS, the abbreviation of liaoshendao. These indicated that the accessions were approved by Liaoning province. SS, the abbreviation of sushendao. These indicated that the accessions were approved by Jiangsu province. SZS, the abbreviation of suzhongshen. These indicated the accessions were approved by Jiangsu province. WPS, the abbreviation of wanpinshen. These indicated that the accessions were approved by Anhui province. WS, the abbreviation of wanshendao. These indicated that the accessions were approved by Anhui province. XS, the abbreviation of xiangshendao. These indicated that the accessions were approved by Hunan province. YS, the abbreviation of yueshendao. These indicated that the accessions were approved by Guangdong province. SC, southern China; CC, central China; EC, eastern China; NEC, northeastern China; SWC, southwest China; JP, Japan; SEA, southeast Asia

**Supplementary Table 2**

Table S2. Names and origins of 162 rice accessions used for association mapping and the corresponding Q values calculated by STRUCTURE software.

| Code | Germplasm name | Q1 | Q2 |
| --- | --- | --- | --- |
| 1 | Longjing 22 | 0.005 | 0.995 |
| 2 | Longjing 28 | 0.051 | 0.949 |
| 3 | Longjing 27 | 0.019 | 0.981 |
| 4 | 24248 | 0.204 | 0.796 |
| 5 | Tijin | 0.124 | 0.876 |
| 6 | Zhongguo 91 | 0.079 | 0.92 |
| 7 | Kangbingyueguang | 0 | 1 |
| 8 | Sihao 4385 | 0 | 1 |
| 9 | Nannongjing 1R | 0.299 | 0.701 |
| 10 | Hongmangshajing | 0.968 | 0.032 |
| 11 | Wanhuangdao | 0.114 | 0.886 |
| 12 | Xudao 3hao | 0 | 1 |
| 13 | Youzhiyueguang | 0 | 1 |
| 14 | Yuedao 68 | 1 | 0 |
| 15 | Longdao 8hao | 0.058 | 0.942 |
| 16 | Longdao 6hao | 0.057 | 0.943 |
| 17 | Qiutianxiaoding | 0.002 | 0.998 |
| 18 | Zhenghan 2hao | 0 | 1 |
| 19 | Xiangjing 9407 | 0.035 | 0.965 |
| 20 | Nongxiang 21 | 1 | 0 |
| 21 | Fengyouwan 8hao | 1 | 0 |
| 22 | Xiangwanxian 17 | 0.121 | 0.879 |
| 23 | Yuedao 37 | 1 | 0 |
| 24 | Sujing 353 | 0.014 | 0.986 |
| 25 | Zhen9424 | 0 | 1 |
| 26 | Baikenuo | 0.981 | 0.019 |
| 27 | Diantun502xuanzao | 1 | 0 |
| 28 | Yuedao 41 | 1 | 0 |
| 29 | Longnuo 3hao | 0.047 | 0.953 |
| 30 | Mudanjiang 28 | 0.077 | 0.923 |
| 31 | Yujing 6hao | 0.002 | 0.998 |
| 32 | Shengdao808 | 0.024 | 0.976 |
| 33 | Yuedao 32 | 1 | 0 |
| 34 | Yuedao 107 | 0.953 | 0.047 |
| 35 | Yuedao 61 | 1 | 0 |
| 36 | Yuedao 50 | 1 | 0 |
| 37 | Yuedao 109 | 1 | 0 |
| 38 | Yuedao 62 | 1 | 0 |
| 39 | Yuedao 66 | 1 | 0 |
| 40 | Hongnong 5hao | 0.003 | 0.997 |
| 41 | Suyunuo | 0 | 1 |
| 42 | Shenlenuo | 0.078 | 0.922 |
| 43 | Hongjiaozhan | 0.973 | 0.027 |
| 44 | Wanjingnuo | 0.001 | 0.999 |
| 45 | Nongxiang26 | 1 | 0 |
| 46 | Yuedao 9 | 1 | 0 |
| 47 | M1004 | 0.106 | 0.894 |
| 48 | Nongxiang 25 | 1 | 0 |
| 49 | Longjing 20 | 0.039 | 0.961 |
| 50 | Xiangchuanwuxinbaimi | 0.949 | 0.051 |
| 51 | Jindao 1007 | 0.038 | 0.962 |
| 52 | Zaijinjing | 0.049 | 0.951 |
| 53 | Malaihong | 0.782 | 0.218 |
| 54 | Nannongjing3786 | 0.028 | 0.972 |
| 55 | Wuyunjing 8hao | 0 | 1 |
| 56 | Yuzhenxiang | 0.651 | 0.349 |
| 57 | Yuedao24(LCV18) | 1 | 0 |
| 58 | Yuedao 3 | 1 | 0 |
| 59 | Yuedao 43 | 0.98 | 0.02 |
| 60 | Yuedao 48 | 1 | 0 |
| 61 | Yuedao 49 | 1 | 0 |
| 62 | Yuedao 13 | 1 | 0 |
| 63 | Wumangyedao | 0.056 | 0.944 |
| 64 | Haobuqia | 1 | 0 |
| 65 | Yuedao 22 | 1 | 0 |
| 66 | Dongzhengwuyunjing 21 | 0.088 | 0.912 |
| 67 | Yandao 8hao | 0 | 1 |
| 68 | Huaidao 11hao | 0 | 1 |
| 69 | Nannongjing 004 | 0.002 | 0.998 |
| 70 | Zhongzuo 93 | 0 | 1 |
| 71 | Xudao 5hao | 0 | 1 |
| 72 | Huaidao 8hao | 0 | 1 |
| 73 | Dongzheng 1640 | 0.017 | 0.983 |
| 74 | Yanjing 8hao | 0 | 1 |
| 75 | Huifeng 2 | 0 | 1 |
| 76 | Yandao 9hao | 0 | 1 |
| 77 | Lianjing 4hao | 0.038 | 0.962 |
| 78 | Huifeng 1 | 0 | 1 |
| 79 | Sihao 4280 | 0 | 1 |
| 80 | Sihao 4330 | 0 | 1 |
| 81 | Sihao 4259 | 0.06 | 0.94 |
| 82 | Zhengdao 18 | 0 | 1 |
| 83 | Jingnuo 330 | 0.026 | 0.974 |
| 84 | Zhongjing 212 | 0.063 | 0.937 |
| 85 | Wuyunjing 21hao | 0 | 1 |
| 86 | Huajing 6hao | 0.025 | 0.975 |
| 87 | Yangfujing 8hao | 0 | 1 |
| 88 | Zhendao 99 | 0 | 1 |
| 89 | Ningjing 2hao | 0 | 1 |
| 90 | Huajing 5hao | 0.199 | 0.801 |
| 91 | Zhongjing 9677 | 0.013 | 0.987 |
| 92 | Yangfujing 7hao | 0 | 1 |
| 93 | Yuedao 12 | 1 | 0 |
| 94 | Jianongnuo 2hao | 0.005 | 0.995 |
| 95 | Xudao 4hao | 0.001 | 0.999 |
| 96 | Sihao 4040 | 0.015 | 0.985 |
| 97 | Yanjing 9hao | 0 | 1 |
| 98 | Sihao 4081 | 0.072 | 0.928 |
| 99 | Sihao 4041 | 0.043 | 0.957 |
| 100 | Sihao 4031 | 0.34 | 0.66 |
| 101 | Wandao 68 | 0.103 | 0.897 |
| 102 | Wuxiang99-8 | 0.001 | 0.999 |
| 103 | Zhongjing 131 | 0 | 1 |
| 104 | Ningjinghui 260 | 0.026 | 0.974 |
| 105 | Ningjinghui 237 | 0.129 | 0.871 |
| 106 | Wunuoyihao | 0.002 | 0.998 |
| 107 | Sihao 4029 | 0.024 | 0.976 |
| 108 | Wanqu 429bp | 0.006 | 0.994 |
| 109 | Yangfujing 4901 | 0 | 1 |
| 110 | Yandao 6hao | 0.072 | 0.928 |
| 111 | Cbao | 0.128 | 0.872 |
| 112 | Zhengdao 10hao | 1 | 0 |
| 113 | Baoxintaihuqing | 0.765 | 0.235 |
| 114 | Huaidao 9hao | 0.071 | 0.929 |
| 115 | Xiaobaidao | 0.081 | 0.919 |
| 116 | Yaxuenuo | 0.096 | 0.904 |
| 117 | Yangguang 200 | 0 | 1 |
| 118 | Zaoshirihuangdao | 0.151 | 0.849 |
| 119 | Luohanhuang | 0.07 | 0.93 |
| 120 | Xudao2hao | 0.163 | 0.837 |
| 121 | Xudao9201B | 0.009 | 0.991 |
| 122 | Ebusinuodao | 0.082 | 0.918 |
| 123 | Yueguang | 0 | 1 |
| 124 | Yimuhu | 1 | 0 |
| 125 | Qingkong | 0.001 | 0.999 |
| 126 | RT61 | 0 | 1 |
| 127 | IL38 | 0 | 1 |
| 128 | Liuyezhan | 1 | 0 |
| 129 | Zaoxian 14 | 0.991 | 0.009 |
| 130 | Xu91075 | 1 | 0 |
| 131 | Xudao 25-7 | 0 | 1 |
| 132 | Qing 7 | 1 | 0 |
| 133 | Sihao 4141 | 0 | 1 |
| 134 | Suwujing | 0.999 | 0.001 |
| 135 | 9522B | 0 | 1 |
| 136 | 863B | 0.056 | 0.944 |
| 137 | A7444 | 0.42 | 0.58 |
| 138 | Xiepihuang | 0.065 | 0.935 |
| 139 | Shengtangqing | 0.821 | 0.179 |
| 140 | Chuan 6xian | 0.992 | 0.008 |
| 141 | Chuan 5xian | 0.993 | 0.007 |
| 142 | Shufeng 101 | 1 | 0 |
| 143 | Chengnongshuijing | 1 | 0 |
| 144 | Xiangxiandao 10hao | 0.976 | 0.024 |
| 145 | Ⅱ-32B | 0.98 | 0.02 |
| 146 | Chenwan 3hao | 1 | 0 |
| 147 | Xiangaizao 10hao | 1 | 0 |
| 148 | Yuetai B | 0.992 | 0.008 |
| 149 | Qimiaoxiang 2hao | 1 | 0 |
| 150 | Shengyou 2hao | 0.98 | 0.02 |
| 151 | Guichao 2hao | 1 | 0 |
| 152 | LongtepuB | 1 | 0 |
| 153 | Hainanxian R | 0.996 | 0.004 |
| 154 | Zajiaohaigu | 1 | 0 |
| 155 | Nuohangu | 0.405 | 0.594 |
| 156 | Lincangwazuhangu | 0.409 | 0.591 |
| 157 | Yuedao 55 | 1 | 0 |
| 158 | Yuedao 108 | 0.953 | 0.047 |
| 159 | IR112 | 1 | 0 |
| 160 | IR64 | 1 | 0 |
| 161 | Gendjah Gempol | 0.512 | 0.488 |
| 162 | Shengdao 14 | 0 | 1 |

**Supplementary Table 3**

Table S3. SNP position for panicle traits identified by GWAS in 2019, 2020, and 2021.

| Trait^a^ | QTLs | Chr | SNP | Allele | 2019 | 2020 | 2021 | Model | Reported QTL and Genes |
| --- | --- | --- | --- | --- | --- | --- | --- | --- | --- |
|  |  |  |  |  | *P* Value | *P* Value | *P* Value |  |  |
|  | *qPL1* | 1 | 25009989 | A/G | 4.1×10^-6^ | 5.3×10^-7^ | 8.5×10^-7^ | GLM |  |
|  |  | 1 | 25009989 | A/G | 6.9×10^-6^ | 1.1×10^-6^ | 1.7×10^-6^ | MLM |  |
|  | *qPL5* | 5 | 24034378 | T/G | 7.8×10^-6^ | 5.5×10^-6^ | 5.8×10^-6^ | MLM | *EUI1* |
|  | *qPL6* | 6 | 26935985 | A/G | 4.5×10^-6^ | 8.4×10^-6^ | 7.3×10^-6^ | GLM |  |
| PL |  | 6 | 26935985 | A/G | 9.3×10^-6^ | 7.0×10^-6^ | 8.0×10^-6^ | MLM |  |
|  | *qPL9.1* | 9 | 15393518 | A/C | 1.5×10^-6^ | 3.7×10^-7^ | 3.9×10^-7^ | GLM | *LP1* |
|  |  | 9 | 15393518 | A/C | 1.2×10^-6^ | 2.2×10^-7^ | 2.9×10^-7^ | MLM |  |
|  | *qPL9.2* | 9 | 14388593 | T/C | 4.5×10^-6^ | 8.4×10^-6^ | 8.4×10^-6^ | GLM |  |
|  |  | 9 | 14388593 | T/C | 9.3×10^-6^ | 7.0×10^-6^ | 7.0×10^-6^ | MLM |  |
| TGP | *qTGP4* | 4 | 28917371 | C/T | 5.6×10^-10^ | 3.5×10^-11^ | 4.9×10^-10^ | GLM |  |
|  |  | 4 | 28917371 | C/T |  | 4.9×10^-6^ | 9.7×10^-6^ | MLM |  |
|  | *qFGP4* | 4 | 28917371 | C/T | 3.5×10^-8^ | 4.1×10^-11^ | 2.4×10^-10^ | GLM |  |
|  |  | 4 | 28917371 | C/T |  | 7.1×10^-7^ | 8.6×10^-7^ | MLM |  |
| FGP | *qFGP7* | 7 | 23875734 | C/T |  | 9.3×10^-11^ | 2.1×10^-9^ | GLM | *OsBZR1* |
|  |  | 7 | 23875734 | C/T |  | 2.0×10^-6^ | 4.2×10^-6^ | MLM |  |
|  | *qFGP4* | 11 | 5468906 | C/G | 3.1×10^-6^ | 3.2×10^-6^ | 1.2×10^-6^ | MLM |  |
|  | *qSSR1.1* | 1 | 37937308 | G/A | 3.5×10^-7^ |  | 2.2×10^-6^ | GLM | *sd1* |
|  |  | 1 | 37937308 | G/A | 2.3×10^-6^ |  | 8.5×10^-6^ | MLM |  |
|  | *qSSR1.2* | 1 | 11726684 | A/G |  | 1.5×10^-6^ |  | GLM |  |
| SSR |  | 1 | 11726684 | A/G |  | 6.0×10^-6^ |  | MLM |  |
|  | *qSSR10* | 10 | 12944513 | C/G |  | 1.5×10^-8^ |  | GLM |  |
|  |  | 10 | 12944513 | C/G |  | 3.6×10^-8^ |  | MLM |  |
|  | *qGWP4* | 4 | 28917371 | C/T |  | 7.5×10^-10^ | 1.6×10^-9^ | GLM |  |
| GWP |  | 4 | 28917371 | C/T |  | 5.3×10^-6^ | 6.1×10^-6^ | MLM |  |
|  | *qGWP7* | 7 | 3641619 | T/C | 2.2×10^-8^ |  |  | GLM |  |
|  |  | 7 | 3641619 | T/C | 8.2×10^-6^ |  |  | MLM |  |

a PL: panicle trait, TGP: total grain number per panicle, FGP: filled grain number per panicle, SSR: seed setting rate, GWP: grain weight per panicle, Chr: chromosome.

**Supplementary Table 4**

Table S4. Annotations of candidate genes and information on SNPs in the LD region 24.58-24.81 Mb associated with PL.

| MSU ID | Gene ID | SNP Location | Reference | Alterative | Region | Variationtype | Annotation |
| --- | --- | --- | --- | --- | --- | --- | --- |
| *LOC_Os01g43480* | *Os01g0623500* | 1_24893883 | G | T | upstream |  | polygalacturonase, putative, expressed |
| *LOC_Os01g43480* | *Os01g0623500* | 1_24894002 | A | C | upstream |  |  |
| *LOC_Os01g43480* | *Os01g0623500* | 1_24894133 | A | C | upstream |  |  |
| *LOC_Os01g43480* | *Os01g0623500* | 1_24894460 | C | G | upstream |  |  |
| *LOC_Os01g43480* | *Os01g0623500* | 1_24895763 | T | G | intronic |  |  |
| *LOC_Os01g43480* | *Os01g0623500* | 1_24895881 | A | C | intronic |  |  |
| *LOC_Os01g43480* | *Os01g0623500* | 1_24896132 | C | A | intronic |  |  |
| *LOC_Os01g43480* | *Os01g0623500* | 1_24896190 | G | T | exonic | Nonsynonymous |  |
| *LOC_Os01g43480* | *Os01g0623500* | 1_24896475 | A | G | intronic |  |  |
| *LOC_Os01g43480* | *Os01g0623500* | 1_24896492 | A | G | intronic |  |  |
| *LOC_Os01g43480* | *Os01g0623500* | 1_24897157 | A | C | exonic | Nonsynonymous |  |
| *LOC_Os01g43480* | *Os01g0623500* | 1_24897244 | T | C | exonic | Nonsynonymous |  |
| *LOC_Os01g43480* | *Os01g0623500* | 1_24897429 | T | G | exonic | Nonsynonymous |  |
| *LOC_Os01g43480* | *Os01g0623500* | 1_24898241 | C | T | exonic | synonymous |  |
| *LOC_Os01g43480* | *Os01g0623500* | 1_24898348 | C | G | intronic |  |  |
| *LOC_Os01g43480* | *Os01g0623500* | 1_24898570 | G | C | intronic |  |  |
| *LOC_Os01g43480* | *Os01g0623500* | 1_24898592 | C | T | intronic |  |  |
| *LOC_Os01g43480* | *Os01g0623500* | 1_24898706 | G | A | intronic |  |  |
| *LOC_Os01g43480* | *Os01g0623500* | 1_24898743 | C | T | intronic |  |  |
| *LOC_Os01g43480* | *Os01g0623500* | 1_24898859 | A | T | exonic | Nonsynonymous |  |
| *LOC_Os01g43480* | *Os01g0623500* | 1_24899286 | G | C | UTR3 |  |  |
| *LOC_Os01g43480* | *Os01g0623500* | 1_24899318 | T | C | UTR3 |  |  |
| *LOC_Os01g43480* | *Os01g0623500* | 1_24898348 | C | G | intronic |  |  |
| *LOC_Os01g43480* | *Os01g0623500* | 1_24898570 | G | C | intronic |  |  |
| *LOC_Os01g43480* | *Os01g0623500* | 1_24898592 | C | T | intronic |  |  |
| *LOC_Os01g43480* | *Os01g0623500* | 1_24898706 | G | A | intronic |  |  |
| *LOC_Os01g43480* | *Os01g0623500* | 1_24898743 | C | T | intronic |  |  |
| *LOC_Os01g43520* | *Os01g0624000* | 1_24912634 | C | T | downstream |  | neutral ceramidase precursor, putative, expressed |
| *LOC_Os01g43520* | *Os01g0624000* | 1_24912665 | C | T | downstream |  |  |
| *LOC_Os01g43520* | *Os01g0624000* | 1_24912773 | T | C | downstream |  |  |
| *LOC_Os01g43520* | *Os01g0624000* | 1_24913635 | G | A | UTR3 |  |  |
| *LOC_Os01g43520* | *Os01g0624000* | 1_24913701 | C | T | UTR3 |  |  |
| *LOC_Os01g43520* | *Os01g0624000* | 1_24913780 | T | G | UTR3 |  |  |
| *LOC_Os01g43520* | *Os01g0624000* | 1_24914195 | T | C | exonic |  |  |
| *LOC_Os01g43520* | *Os01g0624000* | 1_24918863 | C | T | exonic |  |  |
| *LOC_Os01g43520* | *Os01g0624000* | 1_24919628 | G | A | exonic |  |  |
| *LOC_Os01g43520* | *Os01g0624000* | 1_24919667 | G | A | exonic |  |  |
| *LOC_Os01g43520* | *Os01g0624000* | 1_24919962 | T | C | exonic | synonymous |  |
| *LOC_Os01g43520* | *Os01g0624000* | 1_24920124 | C | A | exonic | synonymous |  |
| *LOC_Os01g43520* | *Os01g0624000* | 1_24922459 | T | G | exonic | Nonsynonymous |  |
| *LOC_Os01g43520* | *Os01g0624000* | 1_24922716 | T | C | exonic | Nonsynonymous |  |
| *LOC_Os01g43520* | *Os01g0624000* | 1_24922799 | T | A | UTR5 |  |  |
| *LOC_Os01g43520* | *Os01g0624000* | 1_24922858 | A | G | intronic |  |  |
| *LOC_Os01g43520* | *Os01g0624000* | 1_24923020 | G | A | intronic |  |  |
| *LOC_Os01g43520* | *Os01g0624000* | 1_24923204 | T | C | intronic |  |  |
| *LOC_Os01g43520* | *Os01g0624000* | 1_24923596 | G | A | UTR5 |  |  |
| *LOC_Os01g43520* | *Os01g0624000* | 1_24924494 | G | A | upstream |  |  |
| *LOC_Os01g43520* | *Os01g0624000* | 1_24924640 | C | T | upstream |  |  |
| *LOC_Os01g43520* | *Os01g0624000* | 1_24924799 | C | T | upstream |  |  |
| *LOC_Os01g43520* | *Os01g0624000* | 1_24924822 | G | A | upstream |  |  |
| *LOC_Os01g43520* | *Os01g0624000* | 1_24924830 | A | G | upstream |  |  |
| *LOC_Os01g43520* | *Os01g0624000* | 1_24924899 | C | A | upstream |  |  |
| *LOC_Os01g43520* | *Os01g0624000* | 1_24924964 | A | G | upstream |  |  |
| *LOC_Os01g43520* | *Os01g0624000* | 1_24924977 | A | C | upstream |  |  |
| *LOC_Os01g43530* | *Os01g0624400* | 1_24932180 | T | C | upstream |  | late embryogenesis abundant protein, putative, expressed |
| *LOC_Os01g43530* | *Os01g0624400* | 1_24932310 | C | T | upstream |  |  |
| *LOC_Os01g43530* | *Os01g0624400* | 1_24932357 | T | C | upstream |  |  |
| *LOC_Os01g43530* | *Os01g0624400* | 1_24933207 | A | G | exonic | Nonsynonymous |  |
| *LOC_Os01g43530* | *Os01g0624400* | 1_24933529 | T | G | exonic | synonymous |  |
| *LOC_Os01g43530* | *Os01g0624400* | 1_24933664 | C | A | UTR3 |  |  |
| *LOC_Os01g43530* | *Os01g0624400* | 1_24933825 | T | G | downstream |  |  |
| *LOC_Os01g43530* | *Os01g0624400* | 1_24934008 | C | T | downstream |  |  |
| *LOC_Os01g43530* | *Os01g0624400* | 1_24934032 | A | T | downstream |  |  |
| *LOC_Os01g43530* | *Os01g0624400* | 1_24934082 | C | A | downstream |  |  |
| *LOC_Os01g43530* | *Os01g0624400* | 1_24934211 | G | A | downstream |  |  |
| *LOC_Os01g43530* | *Os01g0624400* | 1_24934223 | T | G | downstream |  |  |
| *LOC_Os01g43530* | *Os01g0624400* | 1_24934317 | C | A | downstream |  |  |
| *LOC_Os01g43530* | *Os01g0624400* | 1_24934416 | A | G | downstream |  |  |
| *LOC_Os01g43530* | *Os01g0624400* | 1_24934429 | A | G | downstream |  |  |
| *LOC_Os01g43530* | *Os01g0624400* | 1_24934581 | A | G | downstream |  |  |
| *LOC_Os01g43530* | *Os01g0624400* | 1_24934630 | T | C | downstream |  |  |
| *LOC_Os01g43540* | *Os01g0624500* | 1_24940477 | C | G | upstream |  | suppressor of G2 allele of SKP1, putative, expressed |
| *LOC_Os01g43540* | *Os01g0624500* | 1_24940581 | G | A | upstream |  |  |
| *LOC_Os01g43540* | *Os01g0624500* | 1_24941122 | G | A | upstream |  |  |
| *LOC_Os01g43540* | *Os01g0624500* | 1_24941630 | A | T | intronic |  |  |
| *LOC_Os01g43540* | *Os01g0624500* | 1_24941855 | T | C | intronic |  |  |
| *LOC_Os01g43540* | *Os01g0624500* | 1_24942155 | T | C | intronic |  |  |
| *LOC_Os01g43540* | *Os01g0624500* | 1_24942861 | A | G | downstream |  |  |
| *LOC_Os01g43540* | *Os01g0624500* | 1_24942961 | G | C | downstream |  |  |
| *LOC_Os01g43540* | *Os01g0624500* | 1_24943058 | C | T | downstream |  |  |
| *LOC_Os01g43540* | *Os01g0624500* | 1_24943138 | T | C | downstream |  |  |
| *LOC_Os01g43540* | *Os01g0624500* | 1_24943185 | G | A | downstream |  |  |
| *LOC_Os01g43540* | *Os01g0624500* | 1_24943637 | G | A | downstream |  |  |
| *LOC_Os01g43540* | *Os01g0624500* | 1_24943673 | A | G | downstream |  |  |
| *LOC_Os01g43550* | *Os01g0624700* | 1_24944909 | G | A | downstream |  | WRKY12, expressed |
| *LOC_Os01g43550* | *Os01g0624700* | 1_24944925 | G | A | downstream |  |  |
| *LOC_Os01g43550* | *Os01g0624700* | 1_24945155 | T | C | downstream |  |  |
| *LOC_Os01g43550* | *Os01g0624700* | 1_24945217 | C | T | downstream |  |  |
| *LOC_Os01g43550* | *Os01g0624700* | 1_24945228 | G | A | downstream |  |  |
| *LOC_Os01g43550* | *Os01g0624700* | 1_24945433 | C | T | UTR3 |  |  |
| *LOC_Os01g43550* | *Os01g0624700* | 1_24945499 | A | C | UTR3 |  |  |
| *LOC_Os01g43550* | *Os01g0624700* | 1_24946174 | T | C | intronic |  |  |
| *LOC_Os01g43550* | *Os01g0624700* | 1_24947310 | T | G | upstream |  |  |
| *LOC_Os01g43550* | *Os01g0624700* | 1_24947382 | T | A | upstream |  |  |
| *LOC_Os01g43550* | *Os01g0624700* | 1_24947533 | A | G | upstream |  |  |
| *LOC_Os01g43550* | *Os01g0624700* | 1_24947624 | A | G | upstream |  |  |
| *LOC_Os01g43550* | *Os01g0624700* | 1_24947634 | C | A | upstream |  |  |
| *LOC_Os01g43580* | *Os01g0625200* | 1_24960468 | T | C | upstream |  | kinesin motor domain containing protein, putative, expressed |
| *LOC_Os01g43580* | *Os01g0625200* | 1_24961409 | A | G | intronic |  |  |
| *LOC_Os01g43580* | *Os01g0625200* | 1_24962404 | A | T | UTR3 |  |  |
| *LOC_Os01g43580* | *Os01g0625200* | 1_24962410 | G | A | UTR3 |  |  |
| *LOC_Os01g43580* | *Os01g0625200* | 1_24962496 | A | T | UTR3 |  |  |
| *LOC_Os01g43580* | *Os01g0625200* | 1_24962954 | G | A | downstream |  |  |
| *LOC_Os01g43580* | *Os01g0625200* | 1_24962983 | A | G | downstream |  |  |
| *LOC_Os01g43580* | *Os01g0625200* | 1_24963019 | T | C | downstream |  |  |
| *LOC_Os01g43580* | *Os01g0625200* | 1_24963067 | A | T | downstream |  |  |
| *LOC_Os01g43580* | *Os01g0625200* | 1_24963294 | T | C | downstream |  |  |
| *LOC_Os01g43580* | *Os01g0625200* | 1_24963334 | G | T | downstream |  |  |
| *LOC_Os01g43580* | *Os01g0625200* | 1_24963388 | G | A | downstream |  |  |
| *LOC_Os01g43580* | *Os01g0625200* | 1_24963424 | A | C | downstream |  |  |
| *LOC_Os01g43580* | *Os01g0625200* | 1_24963539 | T | A | downstream |  |  |
| *LOC_Os01g43580* | *Os01g0625200* | 1_24963560 | G | A | downstream |  |  |
| *LOC_Os01g43590* | *Os01g0625300* | 1_24966410 | G | T | upstream |  | HSF-type DNA-binding domain containing protein, expressed |
| *LOC_Os01g43590* | *Os01g0625300* | 1_24967015 | G | A | upstream |  |  |
| *LOC_Os01g43590* | *Os01g0625300* | 1_24967038 | G | A | upstream |  |  |
| *LOC_Os01g43590* | *Os01g0625300* | 1_24967366 | T | A | upstream |  |  |
| *LOC_Os01g43590* | *Os01g0625300* | 1_24967382 | T | G | upstream |  |  |
| *LOC_Os01g43590* | *Os01g0625300* | 1_24969753 | A | G | downstream |  |  |
| *LOC_Os01g43610* | *Os01g0625900* | 1_24980457 | C | G | downstream |  | DUF623 domain containing protein, expressed |
| *LOC_Os01g43610* | *Os01g0625900* | 1_24980642 | C | A | downstream |  |  |
| *LOC_Os01g43610* | *Os01g0625900* | 1_24980741 | G | C | downstream |  |  |
| *LOC_Os01g43610* | *Os01g0625900* | 1_24980849 | A | G | downstream |  |  |
| *LOC_Os01g43610* | *Os01g0625900* | 1_24981103 | G | A | downstream |  |  |
| *LOC_Os01g43610* | *Os01g0625900* | 1_24981133 | T | C | downstream |  |  |
| *LOC_Os01g43610* | *Os01g0625900* | 1_24981495 | A | G | UTR3 |  |  |
| *LOC_Os01g43610* | *Os01g0625900* | 1_24982499 | T | G | exonic | synonymous |  |
| *LOC_Os01g43610* | *Os01g0625900* | 1_24983029 | A | C | upstream |  |  |
| *LOC_Os01g43610* | *Os01g0625900* | 1_24983042 | A | G | upstream |  |  |
| *LOC_Os01g43610* | *Os01g0625900* | 1_24983574 | C | T | upstream |  |  |
| *LOC_Os01g43630* | *Os01g0626100* | 1_24993976 | T | C | upstream |  | adaptin, putative, expressed |
| *LOC_Os01g43630* | *Os01g0626100* | 1_24994047 | G | C | upstream |  |  |
| *LOC_Os01g43630* | *Os01g0626100* | 1_24994150 | G | C | upstream |  |  |
| *LOC_Os01g43630* | *Os01g0626100* | 1_24995720 | C | G | UTR3 |  |  |
| *LOC_Os01g43630* | *Os01g0626100* | 1_24995967 | G | T | UTR3 |  |  |
| *LOC_Os01g43630* | *Os01g0626100* | 1_24996020 | T | C | UTR3 |  |  |
| *LOC_Os01g43630* | *Os01g0626100* | 1_24996077 | T | C | UTR3 |  |  |
| *LOC_Os01g43630* | *Os01g0626100* | 1_24996250 | A | C | UTR3 |  |  |
| *LOC_Os01g43630* | *Os01g0626100* | 1_24996320 | G | T | UTR3 |  |  |
| *LOC_Os01g43630* | *Os01g0626100* | 1_24996756 | A | T | UTR3 |  |  |
| *LOC_Os01g43630* | *Os01g0626100* | 1_24997163 | T | C | UTR3 |  |  |
| *LOC_Os01g43630* | *Os01g0626100* | 1_24997506 | C | T | UTR3 |  |  |
| *LOC_Os01g43630* | *Os01g0626100* | 1_24997700 | C | T | UTR3 |  |  |
| *LOC_Os01g43630* | *Os01g0626100* | 1_24998053 | T | A | downstream |  |  |
| *LOC_Os01g43630* | *Os01g0626100* | 1_24998087 | C | T | downstream |  |  |
| *LOC_Os01g43630* | *Os01g0626100* | 1_24998174 | C | T | downstream |  |  |
| *LOC_Os01g43630* | *Os01g0626100* | 1_24998237 | G | A | downstream |  |  |
| *LOC_Os01g43630* | *Os01g0626100* | 1_24998476 | A | G | downstream |  |  |
| *LOC_Os01g43630* | *Os01g0626100* | 1_24998694 | T | A | downstream |  |  |
| *LOC_Os01g43650* | *Os01g0626400* | 1_25009124 | G | A | upstream |  | WRKY11, expressed |
| *LOC_Os01g43650* | *Os01g0626400* | 1_25009252 | A | G | upstream |  |  |
| *LOC_Os01g43650* | *Os01g0626400* | 1_25009416 | C | G | upstream |  |  |
| *LOC_Os01g43650* | *Os01g0626400* | 1_25009457 | T | C | upstream |  |  |
| *LOC_Os01g43650* | *Os01g0626400* | 1_25009989 | A | G | exonic | synonymous |  |
| *LOC_Os01g43650* | *Os01g0626400* | 1_25010621 | G | A | intronic |  |  |
| *LOC_Os01g43650* | *Os01g0626400* | 1_25010941 | T | C | intronic |  |  |
| *LOC_Os01g43650* | *Os01g0626400* | 1_25010963 | A | G | intronic |  |  |
| *LOC_Os01g43650* | *Os01g0626400* | 1_25012100 | T | G | UTR3 |  |  |
| *LOC_Os01g43650* | *Os01g0626400* | 1_25012824 | G | A | downstream |  |  |
| *LOC_Os01g43650* | *Os01g0626400* | 1_25012915 | A | T | downstream |  |  |
| *LOC_Os01g43650* | *Os01g0626400* | 1_25012924 | C | T | downstream |  |  |
| *LOC_Os01g43650* | *Os01g0626400* | 1_25013051 | C | T | downstream |  |  |
| *LOC_Os01g43650* | *Os01g0626400* | 1_25013108 | G | A | downstream |  |  |
| *LOC_Os01g43650* | *Os01g0626400* | 1_25013136 | C | T | downstream |  |  |
| *LOC_Os01g43680* | *Os01g0626900* | 1_25028967 | C | T | downstream |  | CPuORF29 - conserved peptide uORF-containing transcript, expressed |
| *LOC_Os01g43680* | *Os01g0626900* | 1_25029074 | T | C | downstream |  |  |
| *LOC_Os01g43680* | *Os01g0626900* | 1_25029148 | T | A | downstream |  |  |
| *LOC_Os01g43680* | *Os01g0626900* | 1_25029357 | A | C | downstream |  |  |
| *LOC_Os01g43680* | *Os01g0626900* | 1_25029558 | C | T | downstream |  |  |
| *LOC_Os01g43680* | *Os01g0626900* | 1_25030013 | G | T | UTR3 |  |  |
| *LOC_Os01g43680* | *Os01g0626900* | 1_25030469 | C | T | exonic | synonymous |  |
| *LOC_Os01g43680* | *Os01g0626900* | 1_25030639 | A | T | exonic | Nonsynonymous |  |
| *LOC_Os01g43680* | *Os01g0626900* | 1_25030700 | C | T | exonic |  |  |
| *LOC_Os01g43680* | *Os01g0626900* | 1_25030789 | G | C | exonic |  |  |
| *LOC_Os01g43680* | *Os01g0626900* | 1_25030827 | T | G | exonic |  |  |
| *LOC_Os01g43680* | *Os01g0626900* | 1_25031199 | C | T | UTR5 |  |  |
| *LOC_Os01g43680* | *Os01g0626900* | 1_25031247 | A | C | UTR5 |  |  |
| *LOC_Os01g43680* | *Os01g0626900* | 1_25031264 | C | T | UTR5 |  |  |
| *LOC_Os01g43680* | *Os01g0626900* | 1_25031946 | C | A | intronic |  |  |
| *LOC_Os01g43680* | *Os01g0626900* | 1_25032445 | G | A | UTR5 |  |  |
| *LOC_Os01g43680* | *Os01g0626900* | 1_25032756 | A | G | intronic |  |  |
| *LOC_Os01g43680* | *Os01g0626900* | 1_25032796 | A | G | intronic |  |  |
| *LOC_Os01g43680* | *Os01g0626900* | 1_25032814 | C | A | intronic |  |  |
| *LOC_Os01g43680* | *Os01g0626900* | 1_25032871 | A | T | intronic |  |  |
| *LOC_Os01g43680* | *Os01g0626900* | 1_25032916 | C | T | intronic |  |  |
| *LOC_Os01g43680* | *Os01g0626900* | 1_25033090 | A | C | UTR5 |  |  |
| *LOC_Os01g43700* | *Os01g0627400* | 1_25039886 | T | G | upstream |  | cytochrome P450 72A1, putative, expressed |
| *LOC_Os01g43700* | *Os01g0627400* | 1_25041384 | A | G | intronic |  |  |
| *LOC_Os01g43700* | *Os01g0627400* | 1_25041719 | T | C | intronic |  |  |
| *LOC_Os01g43700* | *Os01g0627400* | 1_25041967 | G | A | exonic |  |  |
| *LOC_Os01g43700* | *Os01g0627400* | 1_25042266 | C | T | intronic |  |  |
| *LOC_Os01g43700* | *Os01g0627400* | 1_25042395 | C | A | intronic |  |  |
| *LOC_Os01g43700* | *Os01g0627400* | 1_25042501 | A | G | intronic |  |  |
| *LOC_Os01g43700* | *Os01g0627400* | 1_25042629 | C | T | intronic |  |  |
| *LOC_Os01g43700* | *Os01g0627400* | 1_25042849 | A | C | exonic | synonymous |  |
| *LOC_Os01g43700* | *Os01g0627400* | 1_25043081 | G | T | exonic | Nonsynonymous |  |
| *LOC_Os01g43700* | *Os01g0627400* | 1_25043192 | T | G | intronic |  |  |
| *LOC_Os01g43700* | *Os01g0627400* | 1_25043571 | G | A | exonic | synonymous |  |
| *LOC_Os01g43700* | *Os01g0627400* | 1_25043721 | T | G | UTR3 |  |  |
| *LOC_Os01g43700* | *Os01g0627400* | 1_25044763 | G | A | downstream |  |  |
| *LOC_Os01g43700* | *Os01g0627400* | 1_25044774 | C | T | downstream |  |  |
| *LOC_Os01g43700* | *Os01g0627400* | 1_25044825 | G | A | downstream |  |  |
| *LOC_Os01g43700* | *Os01g0627400* | 1_25044868 | A | G | downstream |  |  |
| *LOC_Os01g43700* | *Os01g0627400* | 1_25044869 | C | T | downstream |  |  |
| *LOC_Os01g43700* | *Os01g0627400* | 1_25044763 | G | A | downstream |  |  |
| *LOC_Os01g43700* | *Os01g0627400* | 1_25044774 | C | T | downstream |  |  |
| *LOC_Os01g43700* | *Os01g0627400* | 1_25044825 | G | A | downstream |  |  |
| *LOC_Os01g43700* | *Os01g0627400* | 1_25044868 | A | G | downstream |  |  |
| *LOC_Os01g43700* | *Os01g0627400* | 1_25044869 | C | T | downstream |  |  |
| *LOC_Os01g43710* | *Os01g0627500* | 1_25046485 | C | T | intronic |  | cytochrome P450 72A1, putative, expressed |
| *LOC_Os01g43710* | *Os01g0627500* | 1_25046608 | T | G | intronic |  |  |
| *LOC_Os01g43710* | *Os01g0627500* | 1_25047036 | C | T | exonic |  |  |
| *LOC_Os01g43710* | *Os01g0627500* | 1_25047154 | C | T | intronic |  |  |
| *LOC_Os01g43710* | *Os01g0627500* | 1_25047342 | A | C | intronic |  |  |
| *LOC_Os01g43710* | *Os01g0627500* | 1_25047359 | G | T | intronic |  |  |
| *LOC_Os01g43710* | *Os01g0627500* | 1_25047407 | T | C | intronic |  |  |
| *LOC_Os01g43710* | *Os01g0627500* | 1_25048562 | A | T | intronic |  |  |
| *LOC_Os01g43710* | *Os01g0627500* | 1_25048572 | C | T | intronic |  |  |
| *LOC_Os01g43710* | *Os01g0627500* | 1_25048811 | T | A | intronic |  |  |
| *LOC_Os01g43720* | *Os01g0627600* | 1_25054856 | G | T | downstream |  | cytochrome P450 72A1, putative, expressed |
| *LOC_Os01g43720* | *Os01g0627600* | 1_25054939 | C | A | downstream |  |  |
| *LOC_Os01g43720* | *Os01g0627600* | 1_25056763 | C | A | exonic |  |  |
| *LOC_Os01g43720* | *Os01g0627600* | 1_25057954 | G | C | exonic |  |  |
| *LOC_Os01g43720* | *Os01g0627600* | 1_25057973 | G | T | exonic |  |  |
| *LOC_Os01g43740* | *Os01g0627800* | 1_25060115 | C | T | upstream |  | cytochrome P450 72A1, putative, expressed |
| *LOC_Os01g43740* | *Os01g0627800* | 1_25060125 | C | T | upstream |  |  |
| *LOC_Os01g43740* | *Os01g0627800* | 1_25060247 | G | A | upstream |  |  |
| *LOC_Os01g43740* | *Os01g0627800* | 1_25060291 | A | T | upstream |  |  |
| *LOC_Os01g43740* | *Os01g0627800* | 1_25060348 | A | G | upstream |  |  |
| *LOC_Os01g43740* | *Os01g0627800* | 1_25061359 | T | C | intronic |  |  |
| *LOC_Os01g43740* | *Os01g0627800* | 1_25061514 | A | G | intronic |  |  |
| *LOC_Os01g43740* | *Os01g0627800* | 1_25061532 | C | T | intronic |  |  |
| *LOC_Os01g43740* | *Os01g0627800* | 1_25062003 | A | G | exonic |  |  |
| *LOC_Os01g43740* | *Os01g0627800* | 1_25062172 | A | G | exonic |  |  |
| *LOC_Os01g43740* | *Os01g0627800* | 1_25062874 | T | C | exonic |  |  |
| *LOC_Os01g43740* | *Os01g0627800* | 1_25063089 | A | G | exonic |  |  |
| *LOC_Os01g43740* | *Os01g0627800* | 1_25063132 | C | T | exonic |  |  |
| *LOC_Os01g43740* | *Os01g0627800* | 1_25063150 | T | C | exonic |  |  |
| *LOC_Os01g43740* | *Os01g0627800* | 1_25063164 | C | T | exonic |  |  |
| *LOC_Os01g43740* | *Os01g0627800* | 1_25063280 | C | A | exonic |  |  |
| *LOC_Os01g43740* | *Os01g0627800* | 1_25063366 | A | C | UTR3 |  |  |
| *LOC_Os01g43740* | *Os01g0627800* | 1_25063578 | T | C | downstream |  |  |
| *LOC_Os01g43740* | *Os01g0627800* | 1_25063652 | T | C | downstream |  |  |
| *LOC_Os01g43740* | *Os01g0627800* | 1_25063679 | G | T | downstream |  |  |
| *LOC_Os01g43740* | *Os01g0627800* | 1_25063817 | A | G | downstream |  |  |
| *LOC_Os01g43740* | *Os01g0627800* | 1_25064094 | C | A | downstream |  |  |
| *LOC_Os01g43740* | *Os01g0627800* | 1_25064396 | A | C | downstream |  |  |
| *LOC_Os01g43750* | *Os01g0627900* | 1_25066632 | G | A | exonic |  | cytochrome P450 72A1, putative, expressed |
| *LOC_Os01g43750* | *Os01g0627900* | 1_25066656 | A | G | exonic |  |  |
| *LOC_Os01g43760* | *Os01g0627933* | 1_25071906 | G | T | exonic |  |  |
| *LOC_Os01g43760* | *Os01g0627933* | 1_25072020 | G | A | exonic |  |  |
| *LOC_Os01g43760* | *Os01g0627933* | 1_25072023 | G | A | exonic |  |  |
| *LOC_Os01g43760* | *Os01g0627933* | 1_25072030 | A | G | exonic |  |  |
| *LOC_Os01g43760* | *Os01g0627933* | 1_25072127 | - | A | exonic |  |  |
| *LOC_Os01g43760* | *Os01g0627933* | 1_25072150 | G | A | exonic |  |  |
| *LOC_Os01g43760* | *Os01g0627933* | 1_25072170 | A | G | exonic |  |  |
| *LOC_Os01g43760* | *Os01g0627933* | 1_25072288 | G | A | exonic |  |  |
| *LOC_Os01g43760* | *Os01g0627933* | 1_25072320 | C | T | exonic |  |  |
| *LOC_Os01g43760* | *Os01g0627933* | 1_25072363 | T | C | UTR3 |  |  |
| *LOC_Os01g43760* | *Os01g0627933* | 1_25072564 | A | G | UTR3 |  |  |
| *LOC_Os01g43760* | *Os01g0627933* | 1_25072574 | A | G | UTR3 |  |  |
| *LOC_Os01g43760* | *Os01g0627933* | 1_25072672 | G | T | UTR3 |  |  |
| *LOC_Os01g43760* | *Os01g0627933* | 1_25072673 | C | T | UTR3 |  |  |
| *LOC_Os01g43760* | *Os01g0627933* | 1_25072771 | T | G | downstream |  |  |
| *LOC_Os01g43760* | *Os01g0627933* | 1_25073008 | C | T | downstream |  |  |
| *LOC_Os01g43760* | *Os01g0627933* | 1_25073322 | G | T | downstream |  |  |
| *LOC_Os01g43774* | *Os01g0628000* | 1_25077288 | T | A | UTR3 |  | cytochrome P450 72A1, putative, expressed |
| *LOC_Os01g43774* | *Os01g0628000* | 1_25077366 | C | A | UTR3 |  |  |
| *LOC_Os01g43844* | *Os01g0628700* | 1_25113249 | C | T | upstream |  | cytochrome P450 72A1, putative, expressed |
| *LOC_Os01g43844* | *Os01g0628700* | 1_25113344 | C | T | upstream |  |  |
| *LOC_Os01g43844* | *Os01g0628700* | 1_25113358 | T | C | upstream |  |  |
| *LOC_Os01g43844* | *Os01g0628700* | 1_25113396 | A | G | upstream |  |  |
| *LOC_Os01g43844* | *Os01g0628700* | 1_25113438 | G | A | upstream |  |  |
| *LOC_Os01g43844* | *Os01g0628700* | 1_25113470 | A | C | upstream |  |  |
| *LOC_Os01g43844* | *Os01g0628700* | 1_25113481 | T | C | upstream |  |  |
| *LOC_Os01g43844* | *Os01g0628700* | 1_25113483 | G | C | upstream |  |  |
| *LOC_Os01g43844* | *Os01g0628700* | 1_25113600 | G | A | upstream |  |  |
| *LOC_Os01g43844* | *Os01g0628700* | 1_25115813 | A | T | intronic |  |  |
| *LOC_Os01g43844* | *Os01g0628700* | 1_25116059 | A | C | intronic |  |  |
| *LOC_Os01g43844* | *Os01g0628700* | 1_25116448 | T | A | intronic |  |  |
| *LOC_Os01g43844* | *Os01g0628700* | 1_25116537 | C | T | exonic |  |  |
| *LOC_Os01g43844* | *Os01g0628700* | 1_25116783 | G | T | exonic |  |  |
| *LOC_Os01g43844* | *Os01g0628700* | 1_25117834 | T | C | exonic |  |  |
| *LOC_Os01g43844* | *Os01g0628700* | 1_25117984 | A | G | exonic |  |  |
| *LOC_Os01g43844* | *Os01g0628700* | 1_25118109 | G | A | UTR3 |  |  |
| *LOC_Os01g43844* | *Os01g0628700* | 1_25118345 | A | C | UTR3 |  |  |
| *LOC_Os01g43844* | *Os01g0628700* | 1_25118373 | G | T | downstream |  |  |
| *LOC_Os01g43851* | *Os01g0628900* | 1_25120185 | T | C | exonic |  | cytochrome P450 72A1, putative, expressed |
| *LOC_Os01g43851* | *Os01g0628900* | 1_25120414 | T | A | intronic |  |  |
| *LOC_Os01g43851* | *Os01g0628900* | 1_25120507 | G | T | intronic |  |  |
| *LOC_Os01g43851* | *Os01g0628900* | 1_25120884 | A | C | intronic |  |  |
| *LOC_Os01g43851* | *Os01g0628900* | 1_25121185 | G | A | exonic |  |  |
| *LOC_Os01g43851* | *Os01g0628900* | 1_25121480 | G | T | exonic |  |  |

**Supplementary Table 5**

Table S5. Annotations of candidate genes and information on SNPs in the LD region 15.28-15.51 Mb associated with PL.

| MSU ID | Gene ID | SNP Location | Reference | Alterative | Region | Variationtype | Annotation |
| --- | --- | --- | --- | --- | --- | --- | --- |
| *LOC_Os09g25490* | *Os09g0422500* | 9_15280471 | T | C | upstream |  | CESA9 - cellulose synthase, expressed |
| *LOC_Os09g25490* | *Os09g0422500* | 9_15281712 | G | T | intronic |  |  |
| *LOC_Os09g25490* | *Os09g0422500* | 9_15281715 | C | A | intronic |  |  |
| *LOC_Os09g25490* | *Os09g0422500* | 9_15282227 | G | T | intronic |  |  |
| *LOC_Os09g25490* | *Os09g0422500* | 9_15283153 | T | C | intronic |  |  |
| *LOC_Os09g25490* | *Os09g0422500* | 9_15283679 | T | C | exonic | synonymous |  |
| *LOC_Os09g25490* | *Os09g0422500* | 9_15283700 | T | G | exonic | synonymous |  |
| *LOC_Os09g25490* | *Os09g0422500* | 9_15283996 | A | T | exonic | synonymous |  |
| *LOC_Os09g25490* | *Os09g0422500* | 9_15284552 | G | A | exonic | synonymous |  |
| *LOC_Os09g25490* | *Os09g0422500* | 9_15284594 | A | G | exonic | synonymous |  |
| *LOC_Os09g25490* | *Os09g0422500* | 9_15284642 | G | T | exonic | synonymous |  |
| *LOC_Os09g25490* | *Os09g0422500* | 9_15285186 | G | C | exonic | synonymous |  |
| *LOC_Os09g25490* | *Os09g0422500* | 9_15285192 | C | A | exonic | synonymous |  |
| *LOC_Os09g25490* | *Os09g0422500* | 9_15285225 | G | A | exonic | synonymous |  |
| *LOC_Os09g25490* | *Os09g0422500* | 9_15285444 | T | C | exonic | synonymous |  |
| *LOC_Os09g25490* | *Os09g0422500* | 9_15285606 | G | A | UTR3 |  |  |
| *LOC_Os09g25540* | *Os09g0423200* | 9_15319668 | A | G | upstream |  | receptor-like protein kinase 2 precursor, putative, expressed |
| *LOC_Os09g25540* | *Os09g0423200* | 9_15319692 | C | T | upstream |  |  |
| *LOC_Os09g25540* | *Os09g0423200* | 9_15320146 | T | A | upstream |  |  |
| *LOC_Os09g25540* | *Os09g0423200* | 9_15320515 | C | T | exonic | nonsynonymous |  |
| *LOC_Os09g25540* | *Os09g0423200* | 9_15320712 | C | T | exonic | nonsynonymous |  |
| *LOC_Os09g25540* | *Os09g0423200* | 9_15321174 | G | A | exonic | nonsynonymous |  |
| *LOC_Os09g25540* | *Os09g0423200* | 9_15321395 | G | T | exonic | nonsynonymous |  |
| *LOC_Os09g25540* | *Os09g0423200* | 9_15321465 | A | T | exonic | nonsynonymous |  |
| *LOC_Os09g25540* | *Os09g0423200* | 9_15322414 | C | G | exonic | nonsynonymous |  |
| *LOC_Os09g25540* | *Os09g0423200* | 9_15323398 | G | A | intronic |  |  |
| *LOC_Os09g25540* | *Os09g0423200* | 9_15323403 | A | G | intronic |  |  |
| *LOC_Os09g25540* | *Os09g0423200* | 9_15323518 | A | G | exonic | synonymous |  |
| *LOC_Os09g25540* | *Os09g0423200* | 9_15323544 | G | A | exonic | nonsynonymous |  |
| *LOC_Os09g25540* | *Os09g0423200* | 9_15323611 | T | G | exonic | synonymous |  |
| *LOC_Os09g25540* | *Os09g0423200* | 9_15324660 | G | C | downstream |  |  |
| *LOC_Os09g25540* | *Os09g0423200* | 9_15324705 | A | G | downstream |  |  |
| *LOC_Os09g25550* | *Os09g0423300* | 9_15327945 | A | T | upstream |  | pentatricopeptide, putative, expressed |
| *LOC_Os09g25550* | *Os09g0423300* | 9_15327955 | A | G | upstream |  |  |
| *LOC_Os09g25550* | *Os09g0423300* | 9_15328010 | T | C | upstream |  |  |
| *LOC_Os09g25550* | *Os09g0423300* | 9_15328043 | C | T | upstream |  |  |
| *LOC_Os09g25550* | *Os09g0423300* | 9_15328091 | A | C | upstream |  |  |
| *LOC_Os09g25550* | *Os09g0423300* | 9_15328316 | C | A | upstream |  |  |
| *LOC_Os09g25550* | *Os09g0423300* | 9_15328386 | C | T | upstream |  |  |
| *LOC_Os09g25550* | *Os09g0423300* | 9_15328399 | T | G | upstream |  |  |
| *LOC_Os09g25550* | *Os09g0423300* | 9_15328523 | A | G | upstream |  |  |
| *LOC_Os09g25550* | *Os09g0423300* | 9_15328620 | C | A | upstream |  |  |
| *LOC_Os09g25550* | *Os09g0423300* | 9_15328628 | T | G | upstream |  |  |
| *LOC_Os09g25550* | *Os09g0423300* | 9_15329030 | C | T | intronic |  |  |
| *LOC_Os09g25550* | *Os09g0423300* | 9_15329532 | G | A | exonic | nonsynonymous |  |
| *LOC_Os09g25550* | *Os09g0423300* | 9_15329699 | T | C | exonic | nonsynonymous |  |
| *LOC_Os09g25550* | *Os09g0423300* | 9_15330256 | T | C | exonic | synonymous |  |
| *LOC_Os09g25550* | *Os09g0423300* | 9_15330472 | G | A | exonic | synonymous |  |
| *LOC_Os09g25550* | *Os09g0423300* | 9_15331444 | T | G | exonic | synonymous |  |
| *LOC_Os09g25550* | *Os09g0423300* | 9_15331475 | A | G | exonic | nonsynonymous |  |
| *LOC_Os09g25550* | *Os09g0423300* | 9_15332130 | G | C | UTR3 |  |  |
| *LOC_Os09g25550* | *Os09g0423300* | 9_15332219 | A | G | UTR3 |  |  |
| *LOC_Os09g25550* | *Os09g0423300* | 9_15332240 | T | A | UTR3 |  |  |
| *LOC_Os09g25550* | *Os09g0423300* | 9_15332251 | G | A | UTR3 |  |  |
| *LOC_Os09g25560* | *Os09g0423400* | 9_15333771 | G | A | intronic |  | BT1 family protein, putative, expressed |
| *LOC_Os09g25560* | *Os09g0423400* | 9_15333857 | G | A | intronic |  |  |
| *LOC_Os09g25560* | *Os09g0423400* | 9_15334119 | T | A | intronic |  |  |
| *LOC_Os09g25560* | *Os09g0423400* | 9_15334389 | A | C | intronic |  |  |
| *LOC_Os09g25560* | *Os09g0423400* | 9_15335049 | G | T | intronic |  |  |
| *LOC_Os09g25560* | *Os09g0423400* | 9_15335090 | T | C | intronic |  |  |
| *LOC_Os09g25560* | *Os09g0423400* | 9_15335300 | G | A | intronic |  |  |
| *LOC_Os09g25560* | *Os09g0423400* | 9_15335422 | A | G | intronic |  |  |
| *LOC_Os09g25560* | *Os09g0423400* | 9_15335440 | T | C | exonic | nonsynonymous |  |
| *LOC_Os09g25560* | *Os09g0423400* | 9_15335452 | T | C | exonic | nonsynonymous |  |
| *LOC_Os09g25560* | *Os09g0423400* | 9_15335526 | C | T | exonic | nonsynonymous |  |
| *LOC_Os09g25560* | *Os09g0423400* | 9_15336250 | G | A | intronic |  |  |
| *LOC_Os09g25560* | *Os09g0423400* | 9_15336683 | A | C | intronic |  |  |
| *LOC_Os09g25560* | *Os09g0423400* | 9_15337282 | C | T | intronic |  |  |
| *LOC_Os09g25560* | *Os09g0423400* | 9_15337358 | T | C | intronic |  |  |
| *LOC_Os09g25560* | *Os09g0423400* | 9_15337382 | T | C | intronic |  |  |
| *LOC_Os09g25560* | *Os09g0423400* | 9_15337552 | G | A | intronic |  |  |
| *LOC_Os09g25560* | *Os09g0423400* | 9_15338319 | C | T | UTR3 |  |  |
| *LOC_Os09g25560* | *Os09g0423400* | 9_15338722 | C | T | UTR3 |  |  |
| *LOC_Os09g25560* | *Os09g0423400* | 9_15338830 | A | T | downstream |  |  |
| *LOC_Os09g25560* | *Os09g0423400* | 9_15339046 | C | T | downstream |  |  |
| *LOC_Os09g25560* | *Os09g0423400* | 9_15339203 | C | T | downstream |  |  |
| *LOC_Os09g25560* | *Os09g0423400* | 9_15339278 | A | G | downstream |  |  |
| *LOC_Os09g25560* | *Os09g0423400* | 9_15339321 | G | A | downstream |  |  |
| *LOC_Os09g25560* | *Os09g0423400* | 9_15339411 | A | G | Downstream |  |  |
| *LOC_Os09g25560* | *Os09g0423400* | 9_15339432 | C | G | downstream |  |  |
| *LOC_Os09g25560* | *Os09g0423400* | 9_15339467 | C | T | downstream |  |  |
| *LOC_Os09g25560* | *Os09g0423400* | 9_15339523 | C | T | downstream |  |  |
| *LOC_Os09g25560* | *Os09g0423400* | 9_15339579 | G | T | downstream |  |  |
| *LOC_Os09g25560* | *Os09g0423400* | 9_15339581 | T | C | downstream |  |  |
| *LOC_Os09g25560* | *Os09g0423400* | 9_15339623 | G | A | downstream |  |  |
| *LOC_Os09g25560* | *Os09g0423400* | 9_15339676 | C | T | downstream |  |  |
| *LOC_Os09g25560* | *Os09g0423400* | 9_15339722 | G | A | downstream |  |  |
| *LOC_Os09g25560* | *Os09g0423400* | 9_15339770 | A | G | downstream |  |  |
| *LOC_Os09g25560* | *Os09g0423400* | 9_15339791 | A | G | downstream |  |  |
| *LOC_Os09g25570* | *Os09g0423500* | 9_15343713 | T | C | upstream |  | aspartic proteinase, putative, expressed |
| *LOC_Os09g25570* | *Os09g0423500* | 9_15343803 | T | C | upstream |  |  |
| *LOC_Os09g25570* | *Os09g0423500* | 9_15343811 | T | C | upstream |  |  |
| *LOC_Os09g25570* | *Os09g0423500* | 9_15343843 | C | T | upstream |  |  |
| *LOC_Os09g25570* | *Os09g0423500* | 9_15343862 | C | G | upstream |  |  |
| *LOC_Os09g25570* | *Os09g0423500* | 9_15343864 | T | A | upstream |  |  |
| *LOC_Os09g25570* | *Os09g0423500* | 9_15343910 | C | T | upstream |  |  |
| *LOC_Os09g25570* | *Os09g0423500* | 9_15343922 | T | C | upstream |  |  |
| *LOC_Os09g25570* | *Os09g0423500* | 9_15343938 | G | A | upstream |  |  |
| *LOC_Os09g25570* | *Os09g0423500* | 9_15343948 | C | G | upstream |  |  |
| *LOC_Os09g25570* | *Os09g0423500* | 9_15345167 | G | A | intronic |  |  |
| *LOC_Os09g25570* | *Os09g0423500* | 9_15345334 | A | T | intronic |  |  |
| *LOC_Os09g25580* | *Os09g0423600* | 9_15348115 | G | T | downstream |  | monogalactosyldiacylglycerol synthase, putative, expressed |
| *LOC_Os09g25580* | *Os09g0423600* | 9_15348133 | T | C | downstream |  |  |
| *LOC_Os09g25580* | *Os09g0423600* | 9_15348210 | C | G | downstream |  |  |
| *LOC_Os09g25580* | *Os09g0423600* | 9_15348656 | T | A | downstream |  |  |
| *LOC_Os09g25580* | *Os09g0423600* | 9_15348745 | C | T | downstream |  |  |
| *LOC_Os09g25580* | *Os09g0423600* | 9_15348750 | G | A | downstream |  |  |
| *LOC_Os09g25580* | *Os09g0423600* | 9_15348785 | A | C | downstream |  |  |
| *LOC_Os09g25580* | *Os09g0423600* | 9_15349209 | C | T | UTR3 |  |  |
| *LOC_Os09g25580* | *Os09g0423600* | 9_15349765 | T | G | intronic |  |  |
| *LOC_Os09g25580* | *Os09g0423600* | 9_15350028 | A | G | intronic |  |  |
| *LOC_Os09g25580* | *Os09g0423600* | 9_15350268 | C | T | intronic |  |  |
| *LOC_Os09g25580* | *Os09g0423600* | 9_15350575 | A | C | intronic |  |  |
| *LOC_Os09g25580* | *Os09g0423600* | 9_15351078 | T | C | exonic | synonymous |  |
| *LOC_Os09g25580* | *Os09g0423600* | 9_15351869 | C | G | intronic |  |  |
| *LOC_Os09g25580* | *Os09g0423600* | 9_15352963 | G | T | exonic | synonymous |  |
| *LOC_Os09g25580* | *Os09g0423600* | 9_15353465 | C | A | intronic |  |  |
| *LOC_Os09g25580* | *Os09g0423600* | 9_15353754 | C | G | intronic |  |  |
| *LOC_Os09g25580* | *Os09g0423600* | 9_15354096 | A | G | intronic |  |  |
| *LOC_Os09g25580* | *Os09g0423600* | 9_15354137 | C | A | intronic |  |  |
| *LOC_Os09g25580* | *Os09g0423600* | 9_15355905 | G | T | upstream |  |  |
| *LOC_Os09g25580* | *Os09g0423600* | 9_15355972 | C | A | upstream |  |  |
| *LOC_Os09g25580* | *Os09g0423600* | 9_15356405 | C | T | upstream |  |  |
| *LOC_Os09g25580* | *Os09g0423600* | 9_15356426 | C | T | upstream |  |  |
| *LOC_Os09g25580* | *Os09g0423600* | 9_15356434 | T | C | upstream |  |  |
| *LOC_Os09g25580* | *Os09g0423600* | 9_15356448 | T | G | upstream |  |  |
| *LOC_Os09g25580* | *Os09g0423600* | 9_15356486 | G | A | upstream |  |  |
| *LOC_Os09g25580* | *Os09g0423600* | 9_15356543 | C | T | upstream |  |  |
| *LOC_Os09g25580* | *Os09g0423600* | 9_15356553 | T | C | upstream |  |  |
| *LOC_Os09g25580* | *Os09g0423600* | 9_15356571 | G | A | upstream |  |  |
| *LOC_Os09g25590* | *Os09g0423700* | 9_15357447 | A | G | upstream |  | tsi1-interacting protein TSIP1, putative, expressed |
| *LOC_Os09g25590* | *Os09g0423700* | 9_15357482 | G | A | upstream |  |  |
| *LOC_Os09g25590* | *Os09g0423700* | 9_15357694 | G | A | upstream |  |  |
| *LOC_Os09g25590* | *Os09g0423700* | 9_15357768 | A | G | upstream |  |  |
| *LOC_Os09g25590* | *Os09g0423700* | 9_15357798 | T | C | upstream |  |  |
| *LOC_Os09g25590* | *Os09g0423700* | 9_15357906 | C | T | upstream |  |  |
| *LOC_Os09g25590* | *Os09g0423700* | 9_15357949 | G | C | upstream |  |  |
| *LOC_Os09g25590* | *Os09g0423700* | 9_15358013 | C | T | upstream |  |  |
| *LOC_Os09g25590* | *Os09g0423700* | 9_15358040 | G | A | upstream |  |  |
| *LOC_Os09g25590* | *Os09g0423700* | 9_15358214 | A | T | upstream |  |  |
| *LOC_Os09g25590* | *Os09g0423700* | 9_15358907 | C | T | intronic |  |  |
| *LOC_Os09g25590* | *Os09g0423700* | 9_15359043 | G | T | intronic |  |  |
| *LOC_Os09g25590* | *Os09g0423700* | 9_15359116 | T | G | intronic |  |  |
| *LOC_Os09g25590* | *Os09g0423700* | 9_15359269 | C | T | intronic |  |  |
| *LOC_Os09g25590* | *Os09g0423700* | 9_15359283 | G | T | intronic |  |  |
| *LOC_Os09g25590* | *Os09g0423700* | 9_15359353 | A | T | intronic |  |  |
| *LOC_Os09g25590* | *Os09g0423700* | 9_15359545 | T | C | intronic |  |  |
| *LOC_Os09g25590* | *Os09g0423700* | 9_15359667 | A | G | intronic |  |  |
| *LOC_Os09g25590* | *Os09g0423700* | 9_15359983 | A | T | exonic | nonsynonymous |  |
| *LOC_Os09g25590* | *Os09g0423700* | 9_15360180 | C | T | intronic |  |  |
| *LOC_Os09g25590* | *Os09g0423700* | 9_15360201 | C | T | intronic |  |  |
| *LOC_Os09g25590* | *Os09g0423700* | 9_15360213 | A | C | intronic |  |  |
| *LOC_Os09g25590* | *Os09g0423700* | 9_15360741 | A | G | downstream |  |  |
| *LOC_Os09g25590* | *Os09g0423700* | 9_15360781 | T | C | downstream |  |  |
| *LOC_Os09g25590* | *Os09g0423700* | 9_15360782 | G | A | downstream |  |  |
| *LOC_Os09g25590* | *Os09g0423700* | 9_15360891 | C | G | downstream |  |  |
| *LOC_Os09g25590* | *Os09g0423700* | 9_15361012 | A | G | downstream |  |  |
| *LOC_Os09g25590* | *Os09g0423700* | 9_15361126 | A | G | downstream |  |  |
| *LOC_Os09g25590* | *Os09g0423700* | 9_15361320 | C | T | downstream |  |  |
| *LOC_Os09g25600* | *Os09g0423800* | 9_15364441 | A | C | upstream |  | AP2 domain containing protein, expressed |
| *LOC_Os09g25600* | *Os09g0423800* | 9_15364783 | A | C | upstream |  |  |
| *LOC_Os09g25600* | *Os09g0423800* | 9_15365356 | C | T | intronic |  |  |
| *LOC_Os09g25600* | *Os09g0423800* | 9_15365683 | A | T | intronic |  |  |
| *LOC_Os09g25600* | *Os09g0423800* | 9_15365961 | T | A | intronic |  |  |
| *LOC_Os09g25600* | *Os09g0423800* | 9_15366093 | C | T | intronic |  |  |
| *LOC_Os09g25600* | *Os09g0423800* | 9_15366472 | A | G | intronic |  |  |
| *LOC_Os09g25600* | *Os09g0423800* | 9_15366835 | G | C | intronic |  |  |
| *LOC_Os09g25600* | *Os09g0423800* | 9_15368808 | A | G | exonic | synonymous |  |
| *LOC_Os09g25600* | *Os09g0423800* | 9_15369070 | G | A | exonic | nonsynonymous |  |
| *LOC_Os09g25610* | *Os09g0424200* | 9_15380643 | A | C | upstream |  | class I glutamine amidotransferase, putative, expressed |
| *LOC_Os09g25610* | *Os09g0424200* | 9_15381179 | G | A | upstream |  |  |
| *LOC_Os09g25610* | *Os09g0424200* | 9_15381181 | T | C | upstream |  |  |
| *LOC_Os09g25610* | *Os09g0424200* | 9_15381292 | C | T | upstream |  |  |
| *LOC_Os09g25610* | *Os09g0424200* | 9_15381424 | G | C | upstream |  |  |
| *LOC_Os09g25610* | *Os09g0424200* | 9_15381505 | A | G | upstream |  |  |
| *LOC_Os09g25610* | *Os09g0424200* | 9_15381561 | A | G | upstream |  |  |
| *LOC_Os09g25610* | *Os09g0424200* | 9_15381593 | T | C | upstream |  |  |
| *LOC_Os09g25610* | *Os09g0424200* | 9_15381849 | A | G | exonic | synonymous |  |
| *LOC_Os09g25610* | *Os09g0424200* | 9_15381955 | A | G | exonic | nonsynonymous |  |
| *LOC_Os09g25610* | *Os09g0424200* | 9_15383072 | C | T | intronic |  |  |
| *LOC_Os09g25610* | *Os09g0424200* | 9_15383084 | C | A | intronic |  |  |
| *LOC_Os09g25610* | *Os09g0424200* | 9_15383161 | A | G | intronic |  |  |
| *LOC_Os09g25610* | *Os09g0424200* | 9_15383288 | T | C | intronic |  |  |
| *LOC_Os09g25610* | *Os09g0424200* | 9_15383466 | G | A | intronic |  |  |
| *LOC_Os09g25610* | *Os09g0424200* | 9_15383647 | G | T | intronic |  |  |
| *LOC_Os09g25610* | *Os09g0424200* | 9_15383672 | T | C | intronic |  |  |
| *LOC_Os09g25610* | *Os09g0424200* | 9_15383693 | A | T | intronic |  |  |
| *LOC_Os09g25610* | *Os09g0424200* | 9_15384037 | G | A | exonic | nonsynonymous |  |
| *LOC_Os09g25610* | *Os09g0424200* | 9_15384328 | G | T | UTR3 |  |  |
| *LOC_Os09g25610* | *Os09g0424200* | 9_15384685 | G | A | downstream |  |  |
| *LOC_Os09g25610* | *Os09g0424200* | 9_15384701 | T | C | downstream |  |  |
| *LOC_Os09g25610* | *Os09g0424200* | 9_15384745 | G | A | downstream |  |  |
| *LOC_Os09g25610* | *Os09g0424200* | 9_15384745 | G | A | downstream |  |  |
| *LOC_Os09g25625* | *Os09g0424300* | 9_15386299 | C | T | exonic | nonsynonymous | S-adenosyl-l-methionine decarboxylase leader peptide, putative, expressed |
| *LOC_Os09g25625* | *Os09g0424300* | 9_15386536 | A | G | exonic | nonsynonymous |  |
| *LOC_Os09g25625* | *Os09g0424300* | 9_15386777 | C | T | exonic | synonymous |  |
| *LOC_Os09g25625* | *Os09g0424300* | 9_15386778 | A | G | exonic | nonsynonymous |  |
| *LOC_Os09g25625* | *Os09g0424300* | 9_15387813 | A | G | UTR5 |  |  |
| *LOC_Os09g25625* | *Os09g0424300* | 9_15387913 | T | A | UTR5 |  |  |
| *LOC_Os09g25625* | *Os09g0424300* | 9_15388233 | T | A | UTR5 |  |  |
| *LOC_Os09g25625* | *Os09g0424300* | 9_15388294 | C | T | UTR5 |  |  |
| *LOC_Os09g25625* | *Os09g0424300* | 9_15388391 | A | T | intronic |  |  |
| *LOC_Os09g25625* | *Os09g0424300* | 9_15388783 | G | A | intronic |  |  |
| *LOC_Os09g25625* | *Os09g0424300* | 9_15388826 | C | A | intronic |  |  |
| *LOC_Os09g25625* | *Os09g0424300* | 9_15388930 | G | C | UTR5 |  |  |
| *LOC_Os09g25625* | *Os09g0424300* | 9_15389076 | G | C | UTR5 |  |  |
| *LOC_Os09g25625* | *Os09g0424300* | 9_15389106 | G | T | upstream |  |  |
| *LOC_Os09g25625* | *Os09g0424300* | 9_15389304 | C | A | upstream |  |  |
| *LOC_Os09g25625* | *Os09g0424300* | 9_15389348 | G | A | upstream |  |  |
| *LOC_Os09g25625* | *Os09g0424300* | 9_15389808 | T | C | upstream |  |  |
| *LOC_Os09g25625* | *Os09g0424300* | 9_15389956 | G | A | upstream |  |  |
| *LOC_Os09g25625* | *Os09g0424300* | 9_15389987 | T | A | upstream |  |  |
| *LOC_Os09g25625* | *Os09g0424200* | 9_15384745 | G | A | downstream |  |  |
| *LOC_Os09g25625* | *Os09g0424300* | 9_15386299 | C | T | exonic | nonsynonymous |  |
| *LOC_Os09g25625* | *Os09g0424300* | 9_15386536 | A | G | exonic | nonsynonymous |  |
| *LOC_Os09g25625* | *Os09g0424300* | 9_15386777 | C | T | exonic | synonymous |  |
| *LOC_Os09g25625* | *Os09g0424300* | 9_15386778 | A | G | exonic | nonsynonymous |  |
| *LOC_Os09g25625* | *Os09g0424300* | 9_15387813 | A | G | UTR5 |  |  |
| *LOC_Os09g25625* | *Os09g0424300* | 9_15387913 | T | A | UTR5 |  |  |
| *LOC_Os09g25625* | *Os09g0424300* | 9_15388233 | T | A | UTR5 |  |  |
| *LOC_Os09g25625* | *Os09g0424300* | 9_15388294 | C | T | UTR5 |  |  |
| *LOC_Os09g25625* | *Os09g0424300* | 9_15388391 | A | T | intronic |  |  |
| *LOC_Os09g25625* | *Os09g0424300* | 9_15388783 | G | A | intronic |  |  |
| *LOC_Os09g25625* | *Os09g0424300* | 9_15388826 | C | A | intronic |  |  |
| *LOC_Os09g25625* | *Os09g0424300* | 9_15388930 | G | C | UTR5 |  |  |
| *LOC_Os09g25625* | *Os09g0424300* | 9_15389076 | G | C | UTR5 |  |  |
| *LOC_Os09g25625* | *Os09g0424300* | 9_15389106 | G | T | upstream |  |  |
| *LOC_Os09g25625* | *Os09g0424300* | 9_15389304 | C | A | upstream |  |  |
| *LOC_Os09g25625* | *Os09g0424300* | 9_15389348 | G | A | upstream |  |  |
| *LOC_Os09g25625* | *Os09g0424300* | 9_15389808 | T | C | upstream |  |  |
| *LOC_Os09g25625* | *Os09g0424300* | 9_15389956 | G | A | upstream |  |  |
| *LOC_Os09g25625* | *Os09g0424300* | 9_15389987 | T | A | upstream |  |  |
| *LOC_Os09g25640* | *Os09g0424501* | 9_15399744 | G | A | upstream |  | 5-nucleotidase domain-containing protein, putative, expressed |
| *LOC_Os09g25650* | *Os09g0424701* | 9_15407452 | C | T | downstream |  | GEX2, putative, expressed |
| *LOC_Os09g25650* | *Os09g0424701* | 9_15407604 | G | A | downstream |  |  |
| *LOC_Os09g25650* | *Os09g0424701* | 9_15407625 | A | G | downstream |  |  |
| *LOC_Os09g25650* | *Os09g0424701* | 9_15407628 | A | C | downstream |  |  |
| *LOC_Os09g25650* | *Os09g0424701* | 9_15407644 | G | A | downstream |  |  |
| *LOC_Os09g25650* | *Os09g0424701* | 9_15407716 | C | T | downstream |  |  |
| *LOC_Os09g25650* | *Os09g0424701* | 9_15407717 | T | C | downstream |  |  |
| *LOC_Os09g25650* | *Os09g0424701* | 9_15407725 | C | T | downstream |  |  |
| *LOC_Os09g25650* | *Os09g0424701* | 9_15407747 | T | C | downstream |  |  |
| *LOC_Os09g25650* | *Os09g0424701* | 9_15407785 | A | - | downstream |  |  |
| *LOC_Os09g25650* | *Os09g0424701* | 9_15407890 | G | T | downstream |  |  |
| *LOC_Os09g25650* | *Os09g0424701* | 9_15408111 | T | C | exonic |  |  |
| *LOC_Os09g25650* | *Os09g0424701* | 9_15408254 | G | T | intronic |  |  |
| *LOC_Os09g25650* | *Os09g0424701* | 9_15408671 | T | C | intronic |  |  |
| *LOC_Os09g25650* | *Os09g0424701* | 9_15408697 | T | C | intronic |  |  |
| *LOC_Os09g25650* | *Os09g0424701* | 9_15408844 | T | G | exonic | nonsynonymous |  |
| *LOC_Os09g25650* | *Os09g0424701* | 9_15408884 | A | T | exonic | synonymous |  |
| *LOC_Os09g25650* | *Os09g0424701* | 9_15409773 | G | A | intronic |  |  |
| *LOC_Os09g25650* | *Os09g0424701* | 9_15409912 | C | T | exonic | nonsynonymous |  |
| *LOC_Os09g25650* | *Os09g0424701* | 9_15409991 | C | T | exonic | nonsynonymous |  |
| *LOC_Os09g25650* | *Os09g0424701* | 9_15410433 | G | A | upstream |  |  |
| *LOC_Os09g25650* | *Os09g0424701* | 9_15411071 | A | - | upstream |  |  |
| *LOC_Os09g25650* | *Os09g0424701* | 9_15411071 | A | T | upstream |  |  |
| *LOC_Os09g25650* | *Os09g0424701* | 9_15411224 | T | C | upstream |  |  |
| *LOC_Os09g25650* | *Os09g0424701* | 9_15411335 | A | G | upstream |  |  |
| *LOC_Os09g25700* | *Os09g0425300* | 9_15422629 | A | G | upstream |  | TsetseEP precursor, putative, expressed |
| *LOC_Os09g25700* | *Os09g0425300* | 9_15422701 | G | A | upstream |  |  |
| *LOC_Os09g25700* | *Os09g0425300* | 9_15422830 | A | G | upstream |  |  |
| *LOC_Os09g25700* | *Os09g0425300* | 9_15422924 | A | T | upstream |  |  |
| *LOC_Os09g25700* | *Os09g0425300* | 9_15423088 | A | G | upstream |  |  |
| *LOC_Os09g25700* | *Os09g0425300* | 9_15423119 | C | A | upstream |  |  |
| *LOC_Os09g25700* | *Os09g0425300* | 9_15423174 | G | A | upstream |  |  |
| *LOC_Os09g25700* | *Os09g0425300* | 9_15423243 | G | T | upstream |  |  |
| *LOC_Os09g25700* | *Os09g0425300* | 9_15423333 | C | T | upstream |  |  |
| *LOC_Os09g25700* | *Os09g0425300* | 9_15423572 | G | A | exonic | nonsynonymous |  |
| *LOC_Os09g25700* | *Os09g0425300* | 9_15423741 | C | T | exonic | nonsynonymous |  |
| *LOC_Os09g25700* | *Os09g0425300* | 9_15423798 | A | G | exonic | nonsynonymous |  |
| *LOC_Os09g25700* | *Os09g0425300* | 9_15423803 | A | G | exonic | nonsynonymous |  |
| *LOC_Os09g25700* | *Os09g0425300* | 9_15423863 | A | G | exonic |  |  |
| *LOC_Os09g25700* | *Os09g0425300* | 9_15423900 | C | T | exonic |  |  |
| *LOC_Os09g25700* | *Os09g0425300* | 9_15424344 | T | A | exonic |  |  |
| *LOC_Os09g25700* | *Os09g0425300* | 9_15424450 | T | C | downstream |  |  |
| *LOC_Os09g25700* | *Os09g0425300* | 9_15424590 | G | A | downstream |  |  |
| *LOC_Os09g25700* | *Os09g0425300* | 9_15424593 | G | C | downstream |  |  |
| *LOC_Os09g25700* | *Os09g0425300* | 9_15424625 | T | C | downstream |  |  |
| *LOC_Os09g25700* | *Os09g0425300* | 9_15424747 | T | A | downstream |  |  |
| *LOC_Os09g25700* | *Os09g0425300* | 9_15424763 | C | G | downstream |  |  |
| *LOC_Os09g25700* | *Os09g0425300* | 9_15425060 | C | A | downstream |  |  |
| *LOC_Os09g25700* | *Os09g0425300* | 9_15425130 | G | A | downstream |  |  |
| *LOC_Os09g25700* | *Os09g0425300* | 9_15425134 | C | T | downstream |  |  |
| *LOC_Os09g25720* | *Os09g0425500* | 9_15429911 | C | G | upstream |  | glycine-rich cell wall structural protein 2 precursor, putative, expressed |
| *LOC_Os09g25720* | *Os09g0425500* | 9_15429983 | C | A | upstream |  |  |
| *LOC_Os09g25720* | *Os09g0425500* | 9_15429992 | C | A | upstream |  |  |
| *LOC_Os09g25720* | *Os09g0425500* | 9_15430362 | C | T | upstream |  |  |
| *LOC_Os09g25720* | *Os09g0425500* | 9_15431046 | G | A | exonic |  |  |
| *LOC_Os09g25720* | *Os09g0425500* | 9_15431128 | T | A | exonic |  |  |
| *LOC_Os09g25720* | *Os09g0425500* | 9_15431133 | T | C | exonic |  |  |
| *LOC_Os09g25720* | *Os09g0425500* | 9_15431473 | T | A | downstream |  |  |
| *LOC_Os09g25720* | *Os09g0425500* | 9_15431609 | C | G | downstream |  |  |
| *LOC_Os09g25720* | *Os09g0425500* | 9_15431633 | C | T | downstream |  |  |
| *LOC_Os09g25760* | *Os09g0425900* | 9_15446176 | C | T | exonic |  | tetraspanin family protein, putative, expressed |
| *LOC_Os09g25760* | *Os09g0425900* | 9_15446200 | T | C | exonic |  |  |
| *LOC_Os09g25760* | *Os09g0425900* | 9_15446817 | A | G | downstream |  |  |
| *LOC_Os09g25760* | *Os09g0425900* | 9_15447146 | T | G | downstream |  |  |
| *LOC_Os09g25760* | *Os09g0425900* | 9_15447239 | G | C | downstream |  |  |
| *LOC_Os09g25760* | *Os09g0425900* | 9_15447270 | T | C | downstream |  |  |
| *LOC_Os09g25760* | *Os09g0425900* | 9_15447376 | G | T | downstream |  |  |
| *LOC_Os09g25760* | *Os09g0425900* | 9_15447388 | T | C | downstream |  |  |
| *LOC_Os09g25760* | *Os09g0425900* | 9_15446817 | A | G | downstream |  |  |
| *LOC_Os09g25760* | *Os09g0425900* | 9_15447146 | T | G | downstream |  |  |
| *LOC_Os09g25760* | *Os09g0425900* | 9_15447239 | G | C | downstream |  |  |
| *LOC_Os09g25760* | *Os09g0425900* | 9_15447270 | T | C | downstream |  |  |
| *LOC_Os09g25760* | *Os09g0425900* | 9_15447376 | G | T | downstream |  |  |
| *LOC_Os09g25760* | *Os09g0425900* | 9_15447388 | T | C | downstream |  |  |
| *LOC_Os09g25770* | *Os09g0426000* | 9_15447778 | A | G | UTR3 |  | auxin-induced protein 5NG4, putative, expressed |
| *LOC_Os09g25784* | *Os09g0426100* | 9_15450744 | C | G | UTR3 |  | auxin-induced protein 5NG4, putative, expressed |
| *LOC_Os09g25784* | *Os09g0426100* | 9_15451364 | T | C | UTR3 |  |  |
| *LOC_Os09g25784* | *Os09g0426100* | 9_15451491 | G | A | UTR3 |  |  |
| *LOC_Os09g25784* | *Os09g0426100* | 9_15451509 | C | T | UTR3 |  |  |
| *LOC_Os09g25784* | *Os09g0426100* | 9_15451522 | A | T | UTR3 |  |  |
| *LOC_Os09g25784* | *Os09g0426100* | 9_15451669 | A | C | UTR3 |  |  |
| *LOC_Os09g25784* | *Os09g0426100* | 9_15451798 | G | A | intronic |  |  |
| *LOC_Os09g25784* | *Os09g0426100* | 9_15451802 | G | A | intronic |  |  |
| *LOC_Os09g25784* | *Os09g0426100* | 9_15451805 | A | G | intronic |  |  |
| *LOC_Os09g25784* | *Os09g0426100* | 9_15451808 | A | G | intronic |  |  |
| *LOC_Os09g25784* | *Os09g0426100* | 9_15451812 | G | A | intronic |  |  |
| *LOC_Os09g25784* | *Os09g0426100* | 9_15451854 | C | T | intronic |  |  |
| *LOC_Os09g25784* | *Os09g0426100* | 9_15451862 | A | G | intronic |  |  |
| *LOC_Os09g25784* | *Os09g0426100* | 9_15451870 | C | T | intronic |  |  |
| *LOC_Os09g25784* | *Os09g0426100* | 9_15451949 | A | G | intronic |  |  |
| *LOC_Os09g25784* | *Os09g0426100* | 9_15451985 | G | A | UTR3 |  |  |
| *LOC_Os09g25784* | *Os09g0426100* | 9_15452079 | A | G | UTR3 |  |  |
| *LOC_Os09g25784* | *Os09g0426100* | 9_15452190 | A | T | exonic |  |  |
| *LOC_Os09g25784* | *Os09g0426100* | 9_15452196 | T | C | exonic |  |  |
| *LOC_Os09g25784* | *Os09g0426100* | 9_15452264 | C | T | intronic |  |  |
| *LOC_Os09g25784* | *Os09g0426100* | 9_15452285 | C | T | intronic |  |  |
| *LOC_Os09g25784* | *Os09g0426100* | 9_15452439 | C | T | intronic |  |  |
| *LOC_Os09g25784* | *Os09g0426100* | 9_15454045 | T | A | intronic |  |  |
| *LOC_Os09g25784* | *Os09g0426100* | 9_15454066 | G | A | intronic |  |  |
| *LOC_Os09g25784* | *Os09g0426100* | 9_15454168 | A | C | intronic |  |  |
| *LOC_Os09g25784* | *Os09g0426100* | 9_15454177 | C | T | intronic |  |  |
| *LOC_Os09g25784* | *Os09g0426100* | 9_15454293 | T | C | intronic |  |  |
| *LOC_Os09g25784* | *Os09g0426100* | 9_15454311 | C | T | intronic |  |  |
| *LOC_Os09g25784* | *Os09g0426100* | 9_15454348 | T | G | intronic |  |  |
| *LOC_Os09g25784* | *Os09g0426100* | 9_15454364 | A | T | intronic |  |  |
| *LOC_Os09g25784* | *Os09g0426100* | 9_15454421 | A | G | intronic |  |  |
| *LOC_Os09g25784* | *Os09g0426100* | 9_15454631 | G | A | intronic |  |  |
| *LOC_Os09g25784* | *Os09g0426100* | 9_15454712 | A | C | intronic |  |  |
| *LOC_Os09g25784* | *Os09g0426100* | 9_15454776 | T | C | intronic |  |  |
| *LOC_Os09g25784* | *Os09g0426100* | 9_15454908 | C | T | intronic |  |  |
| *LOC_Os09g25784* | *Os09g0426100* | 9_15454942 | G | A | intronic |  |  |
| *LOC_Os09g25784* | *Os09g0426100* | 9_15455034 | G | A | intronic |  |  |
| *LOC_Os09g25784* | *Os09g0426100* | 9_15455186 | A | G | exonic |  |  |
| *LOC_Os09g25784* | *Os09g0426100* | 9_15455189 | T | G | exonic |  |  |
| *LOC_Os09g25784* | *Os09g0426100* | 9_15455295 | A | G | intronic |  |  |
| *LOC_Os09g25784* | *Os09g0426100* | 9_15455335 | T | G | intronic |  |  |
| *LOC_Os09g25784* | *Os09g0426100* | 9_15455348 | C | T | intronic |  |  |
| *LOC_Os09g25784* | *Os09g0426100* | 9_15455397 | G | A | exonic |  |  |
| *LOC_Os09g25784* | *Os09g0426100* | 9_15455437 | C | T | exonic |  |  |
| *LOC_Os09g25784* | *Os09g0426100* | 9_15455475 | A | G | UTR3 |  |  |
| *LOC_Os09g25784* | *Os09g0426100* | 9_15455528 | C | A | UTR3 |  |  |
| *LOC_Os09g25784* | *Os09g0426100* | 9_15455590 | C | T | UTR3 |  |  |
| *LOC_Os09g25784* | *Os09g0426100* | 9_15455602 | A | G | UTR3 |  |  |
| *LOC_Os09g25784* | *Os09g0426100* | 9_15455649 | C | T | UTR3 |  |  |
| *LOC_Os09g25784* | *Os09g0426100* | 9_15455700 | G | T | exonic | nonsynonymous |  |
| *LOC_Os09g25800* | *Os09g0426200* | 9_15457243 | T | A | downstream |  | auxin-induced protein 5NG4, putative, expressed |
| *LOC_Os09g25800* | *Os09g0426200* | 9_15457759 | T | A | exonic | synonymous |  |
| *LOC_Os09g25800* | *Os09g0426200* | 9_15457881 | C | A | exonic | nonsynonymous |  |
| *LOC_Os09g25800* | *Os09g0426200* | 9_15457882 | C | A | exonic | synonymous |  |
| *LOC_Os09g25800* | *Os09g0426200* | 9_15457924 | C | T | intronic |  |  |
| *LOC_Os09g25800* | *Os09g0426200* | 9_15458640 | C | G | intronic |  |  |
| *LOC_Os09g25800* | *Os09g0426200* | 9_15459001 | C | A | intronic |  |  |
| *LOC_Os09g25800* | *Os09g0426200* | 9_15459487 | C | T | upstream |  |  |
| *LOC_Os09g25800* | *Os09g0426200* | 9_15459495 | T | A | upstream |  |  |
| *LOC_Os09g25810* | *Os09g0426500* | 9_15464782 | A | G | UTR3 |  | nodulin, putative, expressed |
| *LOC_Os09g25810* | *Os09g0426500* | 9_15464805 | T | C | UTR3 |  |  |
| *LOC_Os09g25810* | *Os09g0426500* | 9_15464816 | C | T | UTR3 |  |  |
| *LOC_Os09g25810* | *Os09g0426500* | 9_15464866 | C | T | UTR3 |  |  |
| *LOC_Os09g25810* | *Os09g0426500* | 9_15465185 | T | C | intronic |  |  |
| *LOC_Os09g25810* | *Os09g0426500* | 9_15465196 | T | C | intronic |  |  |
| *LOC_Os09g25810* | *Os09g0426500* | 9_15465211 | A | G | intronic |  |  |
| *LOC_Os09g25810* | *Os09g0426500* | 9_15465294 | T | G | exonic | nonsynonymous |  |
| *LOC_Os09g25810* | *Os09g0426500* | 9_15465511 | T | G | exonic | synonymous |  |
| *LOC_Os09g25810* | *Os09g0426500* | 9_15465895 | A | C | exonic |  |  |
| *LOC_Os09g25810* | *Os09g0426500* | 9_15466399 | G | A | intronic |  |  |
| *LOC_Os09g25810* | *Os09g0426500* | 9_15466659 | A | T | intronic |  |  |
| *LOC_Os09g25810* | *Os09g0426500* | 9_15466698 | G | A | intronic |  |  |
| *LOC_Os09g25810* | *Os09g0426500* | 9_15466847 | A | T | intronic |  |  |
| *LOC_Os09g25810* | *Os09g0426500* | 9_15466922 | C | G | intronic |  |  |
| *LOC_Os09g25810* | *Os09g0426500* | 9_15466955 | C | A | intronic |  |  |
| *LOC_Os09g25810* | *Os09g0426500* | 9_15467385 | T | C | intronic |  |  |
| *LOC_Os09g25810* | *Os09g0426500* | 9_15467403 | A | G | intronic |  |  |
| *LOC_Os09g25810* | *Os09g0426500* | 9_15467601 | C | A | intronic |  |  |
| *LOC_Os09g25810* | *Os09g0426500* | 9_15468081 | G | A | intronic |  |  |
| *LOC_Os09g25810* | *Os09g0426500* | 9_15468445 | G | A | intronic |  |  |
| *LOC_Os09g25810* | *Os09g0426500* | 9_15468483 | G | A | intronic |  |  |
| *LOC_Os09g25850* | *Os09g0426800* | 9_15492044 | A | G | upstream |  | WAX2, putative, expressed |
| *LOC_Os09g25850* | *Os09g0426800* | 9_15492298 | C | T | upstream |  |  |
| *LOC_Os09g25850* | *Os09g0426800* | 9_15492354 | A | G | upstream |  |  |
| *LOC_Os09g25850* | *Os09g0426800* | 9_15492409 | G | T | upstream |  |  |
| *LOC_Os09g25850* | *Os09g0426800* | 9_15492761 | C | G | upstream |  |  |
| *LOC_Os09g25850* | *Os09g0426800* | 9_15492819 | G | C | upstream |  |  |
| *LOC_Os09g25850* | *Os09g0426800* | 9_15495041 | A | C | intronic |  |  |
| *LOC_Os09g25850* | *Os09g0426800* | 9_15495300 | C | G | intronic |  |  |
| *LOC_Os09g25850* | *Os09g0426800* | 9_15495741 | G | A | exonic | synonymous |  |
| *LOC_Os09g25850* | *Os09g0426800* | 9_15496017 | T | C | intronic |  |  |
| *LOC_Os09g25850* | *Os09g0426800* | 9_15496660 | G | C | exonic | synonymous |  |
| *LOC_Os09g25850* | *Os09g0426800* | 9_15496913 | C | T | intronic |  |  |
| *LOC_Os09g25850* | *Os09g0426800* | 9_15497004 | A | C | exonic | synonymous |  |
| *LOC_Os09g25850* | *Os09g0426800* | 9_15497846 | C | T | downstream |  |  |
| *LOC_Os09g25850* | *Os09g0426800* | 9_15497936 | G | A | downstream |  |  |
| *LOC_Os09g25850* | *Os09g0426800* | 9_15497968 | G | A | downstream |  |  |
| *LOC_Os09g25850* | *Os09g0426800* | 9_15497987 | C | T | downstream |  |  |
| *LOC_Os09g25850* | *Os09g0426800* | 9_15497994 | G | A | downstream |  |  |
| *LOC_Os09g25850* | *Os09g0426800* | 9_15498009 | G | A | downstream |  |  |
| *LOC_Os09g25850* | *Os09g0426800* | 9_15498038 | A | G | downstream |  |  |
| *LOC_Os09g25850* | *Os09g0426800* | 9_15498140 | T | C | downstream |  |  |
| *LOC_Os09g25850* | *Os09g0426800* | 9_15498332 | G | C | downstream |  |  |
| *LOC_Os09g25850* | *Os09g0426800* | 9_15498339 | C | T | downstream |  |  |

**Supplementary Table 6**

Table S6. Annotations of candidate genes and information on SNPs in the LD region 28.81-29.17 Mb associated with TGP, FGP and GWP.

| MSU ID | Gene ID | SNP Location | Reference | Alterative | Region | Variationtype | Annotation |
| --- | --- | --- | --- | --- | --- | --- | --- |
| *LOC_Os04g47330* | *Os04g0561200* | 4_28095370 | A | T | downstream |  | rho-GTPase-activating protein-related, putative, expressed |
| *LOC_Os04g47330* | *Os04g0561200* | 4_28095466 | C | T | downstream |  |  |
| *LOC_Os04g47330* | *Os04g0561200* | 4_28096070 | A | T | downstream |  |  |
| *LOC_Os04g47330* | *Os04g0561200* | 4_28096269 | C | T | downstream |  |  |
| *LOC_Os04g47330* | *Os04g0561200* | 4_28096521 | C | G | exonic | synonymous |  |
| *LOC_Os04g47330* | *Os04g0561200* | 4_28098060 | T | A | upstream |  |  |
| *LOC_Os04g47330* | *Os04g0561200* | 4_28098061 | A | G | upstream |  |  |
| *LOC_Os04g47330* | *Os04g0561200* | 4_28098157 | G | A | upstream |  |  |
| *LOC_Os04g47330* | *Os04g0561200* | 4_28098467 | T | C | upstream |  |  |
| *LOC_Os04g47330* | *Os04g0561200* | 4_28098508 | T | C | upstream |  |  |
| *LOC_Os04g47320* | *Os04g0561000* | 4_28090799 | G | A | upstream |  | uncharacterized mscS family protein, putative, expressed |
| *LOC_Os04g47300* | *Os04g0560600* | 4_28079524 | T | G | UTR3 |  | CAMK_CAMK_like.26 - CAMK includes calcium/calmodulin depedent protein kinases, expressed |
| *LOC_Os04g47300* | *Os04g0560600* | 4_28079526 | A | T | UTR3 |  |  |
| *LOC_Os04g47300* | *Os04g0560600* | 4_28079605 | G | A | UTR3 |  |  |
| *LOC_Os04g47290* | *Os04g0560500* | 4_28080049 | C | T | intronic |  | amine oxidase, putative, expressed |
| *LOC_Os04g47300* | *Os04g0560600* | 4_28080429 | G | A | exonic | synonymous | CAMK_CAMK_like.26 - CAMK includes calcium/calmodulin depedent protein kinases, expressed |
| *LOC_Os04g47290* | *Os04g0560500* | 4_28080773 | G | A | intronic |  | amine oxidase, putative, expressed |
| *LOC_Os04g47300* | *Os04g0560600* | 4_28082307 | T | G | UTR5 |  | CAMK_CAMK_like.26 - CAMK includes calcium/calmodulin depedent protein kinases, expressed |
| *LOC_Os04g47300* | *Os04g0560600* | 4_28082431 | C | T | upstream |  |  |
| *LOC_Os04g47300* | *Os04g0560600* | 4_28082613 | A | G | upstream |  |  |
| *LOC_Os04g47300* | *Os04g0560600* | 4_28083241 | A | G | upstream |  |  |
| *LOC_Os04g47300* | *Os04g0560600* | 4_28083259 | A | C | upstream |  |  |
| *LOC_Os04g47280* | *Os04g0560400* | 4_28074912 | C | T | upstream |  | ATFUC1, putative, expressed |
| *LOC_Os04g47280* | *Os04g0560400* | 4_28075046 | A | G | upstream |  |  |
| *LOC_Os04g47280* | *Os04g0560400* | 4_28075098 | A | C | upstream |  |  |
| *LOC_Os04g47280* | *Os04g0560400* | 4_28075246 | A | T | upstream |  |  |
| *LOC_Os04g47280* | *Os04g0560400* | 4_28075279 | G | A | upstream |  |  |
| *LOC_Os04g47280* | *Os04g0560400* | 4_28075337 | C | A | upstream |  |  |
| *LOC_Os04g47290* | *Os04g0560500* | 4_28076330 | G | A | exonic |  | amine oxidase, putative, expressed |
| *LOC_Os04g47290* | *Os04g0560500* | 4_28076562 | T | C | intronic |  |  |
| *LOC_Os04g47290* | *Os04g0560500* | 4_28076594 | T | G | intronic |  |  |
| *LOC_Os04g47290* | *Os04g0560500* | 4_28076720 | A | C | intronic |  |  |
| *LOC_Os04g47290* | *Os04g0560500* | 4_28076950 | T | A | intronic |  |  |
| *LOC_Os04g47290* | *Os04g0560500* | 4_28077027 | T | C | intronic |  |  |
| *LOC_Os04g47290* | *Os04g0560500* | 4_28077235 | A | C | intronic |  |  |
| *LOC_Os04g47290* | *Os04g0560500* | 4_28077332 | C | T | exonic | nonsynonymous |  |
| *LOC_Os04g47290* | *Os04g0560500* | 4_28077509 | C | A | exonic | nonsynonymous |  |
| *LOC_Os04g47290* | *Os04g0560500* | 4_28078201 | A | G | exonic | nonsynonymous |  |
| *LOC_Os04g47290* | *Os04g0560500* | 4_28078467 | C | T | exonic | synonymous |  |
| *LOC_Os04g47290* | *Os04g0560500* | 4_28078904 | C | A | exonic | nonsynonymous |  |
| *LOC_Os04g47290* | *Os04g0560500* | 4_28079360 | T | C | intronic |  |  |
| *LOC_Os04g47290* | *Os04g0560500* | 4_28080049 | C | T | intronic |  |  |
| *LOC_Os04g47290* | *Os04g0560500* | 4_28080773 | G | A | intronic |  |  |
| *LOC_Os04g47290* | *Os04g0560500* | 4_28081051 | G | T | UTR3 |  |  |
| *LOC_Os04g47280* | *Os04g0560400* | 4_28071497 | T | C | UTR3 |  | ATFUC1, putative, expressed |
| *LOC_Os04g47280* | *Os04g0560400* | 4_28071574 | T | C | UTR3 |  |  |
| *LOC_Os04g47280* | *Os04g0560400* | 4_28071713 | C | G | exonic | nonsynonymous |  |
| *LOC_Os04g47280* | *Os04g0560400* | 4_28071727 | G | A | exonic | nonsynonymous |  |
| *LOC_Os04g47280* | *Os04g0560400* | 4_28071741 | A | G | exonic |  |  |
| *LOC_Os04g47280* | *Os04g0560400* | 4_28072191 | C | T | exonic |  |  |
| *LOC_Os04g47280* | *Os04g0560400* | 4_28072439 | A | C | intronic |  |  |
| *LOC_Os04g47280* | *Os04g0560400* | 4_28072463 | C | A | intronic |  |  |
| *LOC_Os04g47280* | *Os04g0560400* | 4_28072960 | A | T | intronic |  |  |
| *LOC_Os04g47280* | *Os04g0560400* | 4_28073016 | T | A | intronic |  |  |
| *LOC_Os04g47280* | *Os04g0560400* | 4_28073048 | G | C | intronic |  |  |
| *LOC_Os04g47280* | *Os04g0560400* | 4_28073319 | C | T | intronic |  |  |
| *LOC_Os04g47280* | *Os04g0560400* | 4_28073412 | A | C | exonic |  |  |
| *LOC_Os04g47280* | *Os04g0560400* | 4_28073978 | G | A | intronic |  |  |
| *LOC_Os04g47280* | *Os04g0560400* | 4_28074912 | C | T | upstream |  |  |
| *LOC_Os04g47280* | *Os04g0560400* | 4_28075046 | A | G | upstream |  |  |
| *LOC_Os04g47280* | *Os04g0560400* | 4_28075098 | A | C | upstream |  |  |
| *LOC_Os04g47280* | *Os04g0560400* | 4_28075246 | A | T | upstream |  |  |
| *LOC_Os04g47280* | *Os04g0560400* | 4_28075279 | G | A | upstream |  |  |
| *LOC_Os04g47280* | *Os04g0560400* | 4_28075337 | C | A | upstream |  |  |
| *LOC_Os04g47270* | *Os04g0560300* | 4_28067414 | T | C | UTR3 |  | amine oxidase, putative, expressed |
| *LOC_Os04g47270* | *Os04g0560300* | 4_28067533 | A | G | UTR3 |  |  |
| *LOC_Os04g47270* | *Os04g0560300* | 4_28067926 | T | C | exonic |  |  |
| *LOC_Os04g47270* | *Os04g0560300* | 4_28068494 | T | C | intronic |  |  |
| *LOC_Os04g47270* | *Os04g0560300* | 4_28068948 | T | G | exonic |  |  |
| *LOC_Os04g47270* | *Os04g0560300* | 4_28069061 | A | T | exonic |  |  |
| *LOC_Os04g47270* | *Os04g0560300* | 4_28069131 | A | G | exonic |  |  |
| *LOC_Os04g47270* | *Os04g0560300* | 4_28069401 | A | C | exonic |  |  |
| *LOC_Os04g47250* | *Os04g0560100* | 4_28061047 | G | A | downstream |  | cytochrome P450, putative, expressed |
| *LOC_Os04g47250* | *Os04g0560100* | 4_28061192 | A | G | downstream |  |  |
| *LOC_Os04g47250* | *Os04g0560100* | 4_28061681 | C | G | exonic |  |  |
| *LOC_Os04g47250* | *Os04g0560100* | 4_28063590 | A | T | UTR5 |  |  |
| *LOC_Os04g47250* | *Os04g0560100* | 4_28064156 | G | A | upstream |  |  |
| *LOC_Os04g47250* | *Os04g0560100* | 4_28064317 | C | T | upstream |  |  |
| *LOC_Os04g47250* | *Os04g0560100* | 4_28064351 | C | A | upstream |  |  |
| *LOC_Os04g47250* | *Os04g0560100* | 4_28064380 | T | C | upstream |  |  |
| *LOC_Os04g47250* | *Os04g0560100* | 4_28064390 | C | T | upstream |  |  |
| *LOC_Os04g47250* | *Os04g0560100* | 4_28064452 | G | A | upstream |  |  |
| *LOC_Os04g47220* | *Os04g0559700* | 4_28049155 | G | A | downstream |  | aquaporin protein, putative, expressed |
| *LOC_Os04g47240* | *Os04g0559800* | 4_28049331 | T | A | UTR3 |  | STE_MEKK_ste11_MAP3K.17 - STE kinases include homologs to sterile 7, sterile 11 and sterile 20 from yeast, expressed |
| *LOC_Os04g47240* | *Os04g0559800* | 4_28049452 | G | C | UTR3 |  |  |
| *LOC_Os04g47240* | *Os04g0559800* | 4_28049642 | T | C | UTR3 |  |  |
| *LOC_Os04g47240* | *Os04g0559800* | 4_28049692 | T | C | UTR3 |  |  |
| *LOC_Os04g47240* | *Os04g0559800* | 4_28049745 | T | C | intronic |  |  |
| *LOC_Os04g47240* | *Os04g0559800* | 4_28050161 | T | A | intronic |  |  |
| *LOC_Os04g47240* | *Os04g0559800* | 4_28050268 | A | G | UTR3 |  |  |
| *LOC_Os04g47240* | *Os04g0559800* | 4_28050666 | C | T | exonic |  |  |
| *LOC_Os04g47240* | *Os04g0559800* | 4_28050823 | C | T | exonic |  |  |
| *LOC_Os04g47240* | *Os04g0559800* | 4_28051001 | T | G | intronic |  |  |
| *LOC_Os04g47240* | *Os04g0559800* | 4_28051088 | T | G | intronic |  |  |
| *LOC_Os04g47240* | *Os04g0559800* | 4_28051417 | T | A | intronic |  |  |
| *LOC_Os04g47240* | *Os04g0559800* | 4_28051434 | A | G | exonic |  |  |
| *LOC_Os04g47240* | *Os04g0559800* | 4_28051684 | T | C | intronic |  |  |
| *LOC_Os04g47240* | *Os04g0559800* | 4_28051970 | T | G | exonic |  |  |
| *LOC_Os04g47240* | *Os04g0559800* | 4_28052234 | A | C | intronic |  |  |
| *LOC_Os04g47240* | *Os04g0559800* | 4_28052508 | A | G | intronic |  |  |
| *LOC_Os04g47240* | *Os04g0559800* | 4_28052687 | C | T | intronic |  |  |
| *LOC_Os04g47240* | *Os04g0559800* | 4_28052801 | T | C | exonic |  |  |
| *LOC_Os04g47240* | *Os04g0559800* | 4_28053537 | C | T | intronic |  |  |
| *LOC_Os04g47240* | *Os04g0559800* | 4_28053553 | A | T | intronic |  |  |
| *LOC_Os04g47240* | *Os04g0559800* | 4_28053739 | G | A | exonic |  |  |
| *LOC_Os04g47240* | *Os04g0559800* | 4_28055009 | C | T | UTR5 |  |  |
| *LOC_Os04g47240* | *Os04g0559800* | 4_28055890 | C | T | intronic |  |  |
| *LOC_Os04g47240* | *Os04g0559800* | 4_28056083 | C | T | intronic |  |  |
| *LOC_Os04g47240* | *Os04g0559800* | 4_28056322 | A | C | intronic |  |  |
| *LOC_Os04g47240* | *Os04g0559800* | 4_28057179 | T | A | upstream |  |  |
| *LOC_Os04g47220* | *Os04g0559700* | 4_28045651 | C | T | upstream |  | aquaporin protein, putative, expressed |
| *LOC_Os04g47220* | *Os04g0559700* | 4_28048044 | C | A | intronic |  |  |
| *LOC_Os04g47220* | *Os04g0559700* | 4_28049155 | G | A | downstream |  |  |
| *LOC_Os04g47190* | *Os04g0559400* | 4_28025568 | C | T | downstream |  | aminotransferase domain containing protein, putative, expressed |
| *LOC_Os04g47190* | *Os04g0559400* | 4_28025575 | G | A | downstream |  |  |
| *LOC_Os04g47190* | *Os04g0559400* | 4_28025729 | A | G | downstream |  |  |
| *LOC_Os04g47190* | *Os04g0559400* | 4_28025990 | A | G | downstream |  |  |
| *LOC_Os04g47190* | *Os04g0559400* | 4_28026159 | T | C | downstream |  |  |
| *LOC_Os04g47190* | *Os04g0559400* | 4_28026225 | G | A | downstream |  |  |
| *LOC_Os04g47190* | *Os04g0559400* | 4_28026507 | A | T | downstream |  |  |
| *LOC_Os04g47190* | *Os04g0559400* | 4_28026929 | C | T | UTR3 |  |  |
| *LOC_Os04g47190* | *Os04g0559400* | 4_28027144 | A | G | intronic |  |  |
| *LOC_Os04g47190* | *Os04g0559400* | 4_28027337 | T | A | intronic |  |  |
| *LOC_Os04g47190* | *Os04g0559400* | 4_28027719 | G | A | intronic |  |  |
| *LOC_Os04g47190* | *Os04g0559400* | 4_28028067 | T | G | intronic |  |  |
| *LOC_Os04g47190* | *Os04g0559400* | 4_28028514 | A | G | intronic |  |  |
| *LOC_Os04g47190* | *Os04g0559400* | 4_28028583 | T | C | intronic |  |  |
| *LOC_Os04g47190* | *Os04g0559400* | 4_28029034 | G | A | intronic |  |  |
| *LOC_Os04g47190* | *Os04g0559400* | 4_28029361 | A | G | intronic |  |  |
| *LOC_Os04g47190* | *Os04g0559400* | 4_28029451 | T | A | exonic |  |  |
| *LOC_Os04g47190* | *Os04g0559400* | 4_28029474 | C | T | exonic |  |  |
| *LOC_Os04g47190* | *Os04g0559400* | 4_28029542 | T | G | exonic |  |  |
| *LOC_Os04g47190* | *Os04g0559400* | 4_28029612 | G | A | intronic |  |  |
| *LOC_Os04g47170* | *Os04g0559100* | 4_28014734 | T | C | upstream |  | ATROPGEF7/ROPGEF7, putative, expressed |
| *LOC_Os04g47170* | *Os04g0559100* | 4_28015532 | G | A | exonic | nonsynonymous |  |
| *LOC_Os04g47170* | *Os04g0559100* | 4_28015756 | G | A | UTR3 |  |  |
| *LOC_Os04g47360* | *Os04g0561500* | 4_28107893 | A | G | upstream |  | OsPOP9 - Putative Prolyl Oligopeptidase homologue, expressed |
| *LOC_Os04g47360* | *Os04g0561500* | 4_28108209 | G | T | upstream |  |  |
| *LOC_Os04g47360* | *Os04g0561500* | 4_28108210 | A | T | upstream |  |  |
| *LOC_Os04g47360* | *Os04g0561500* | 4_28108261 | A | G | upstream |  |  |
| *LOC_Os04g47360* | *Os04g0561500* | 4_28108296 | T | G | upstream |  |  |
| *LOC_Os04g47360* | *Os04g0561500* | 4_28108849 | T | C | UTR5 |  |  |
| *LOC_Os04g47360* | *Os04g0561500* | 4_28109323 | G | T | intronic |  |  |
| *LOC_Os04g47360* | *Os04g0561500* | 4_28109454 | A | G | intronic |  |  |
| *LOC_Os04g47360* | *Os04g0561500* | 4_28109517 | A | G | intronic |  |  |
| *LOC_Os04g47360* | *Os04g0561500* | 4_28109724 | G | A | intronic |  |  |
| *LOC_Os04g47360* | *Os04g0561500* | 4_28110036 | T | G | exonic |  |  |
| *LOC_Os04g47360* | *Os04g0561500* | 4_28111725 | T | G | exonic |  |  |
| *LOC_Os04g47360* | *Os04g0561500* | 4_28111963 | C | T | exonic |  |  |
| *LOC_Os04g47360* | *Os04g0561500* | 4_28112391 | A | G | intronic |  |  |
| *LOC_Os04g47360* | *Os04g0561500* | 4_28112443 | C | T | intronic |  |  |
| *LOC_Os04g47360* | *Os04g0561500* | 4_28112605 | A | C | intronic |  |  |
| *LOC_Os04g47360* | *Os04g0561500* | 4_28112993 | A | G | UTR3 |  |  |
| *LOC_Os04g47360* | *Os04g0561500* | 4_28113369 | G | A | UTR3 |  |  |
| *LOC_Os04g47360* | *Os04g0561500* | 4_28113410 | T | C | UTR3 |  |  |
| *LOC_Os04g47360* | *Os04g0561500* | 4_28113503 | C | T | UTR3 |  |  |
| *LOC_Os04g47360* | *Os04g0561500* | 4_28113521 | C | T | UTR3 |  |  |
| *LOC_Os04g47360* | *Os04g0561500* | 4_28113707 | A | G | downstream |  |  |
| *LOC_Os04g47370* | *Os04g0561600* | 4_28116360 | A | T | intronic |  | coiled-coil domain-containing protein 124, putative, expressed |
| *LOC_Os04g47370* | *Os04g0561600* | 4_28116438 | C | G | intronic |  |  |
| *LOC_Os04g47370* | *Os04g0561600* | 4_28116595 | T | A | intronic |  |  |
| *LOC_Os04g47370* | *Os04g0561600* | 4_28116746 | T | C | intronic |  |  |
| *LOC_Os04g47370* | *Os04g0561600* | 4_28117713 | A | G | downstream |  |  |
| *LOC_Os04g47370* | *Os04g0561600* | 4_28117735 | T | C | downstream |  |  |
| *LOC_Os04g47370* | *Os04g0561600* | 4_28117984 | T | A | downstream |  |  |
| *LOC_Os04g47370* | *Os04g0561600* | 4_28117713 | A | G | downstream |  |  |
| *LOC_Os04g47370* | *Os04g0561600* | 4_28117735 | T | C | downstream |  |  |
| *LOC_Os04g47370* | *Os04g0561600* | 4_28117984 | T | A | downstream |  |  |
| *LOC_Os04g47380* | *Os04g0561700* | 4_28118300 | A | C | UTR3 |  | coiled-coil domain-containing protein 139, putative, expressed |
| *LOC_Os04g47380* | *Os04g0561700* | 4_28118457 | C | T | intronic |  |  |
| *LOC_Os04g47380* | *Os04g0561700* | 4_28118707 | C | T | intronic |  |  |
| *LOC_Os04g47380* | *Os04g0561700* | 4_28118861 | C | T | intronic |  |  |
| *LOC_Os04g47380* | *Os04g0561700* | 4_28119449 | A | G | intronic |  |  |
| *LOC_Os04g47380* | *Os04g0561700* | 4_28119707 | A | C | intronic |  |  |
| *LOC_Os04g47380* | *Os04g0561700* | 4_28120594 | G | A | intronic |  |  |
| *LOC_Os04g47380* | *Os04g0561700* | 4_28120766 | A | G | intronic |  |  |
| *LOC_Os04g47380* | *Os04g0561700* | 4_28120998 | T | G | exonic | nonsynonymous |  |
| *LOC_Os04g47380* | *Os04g0561700* | 4_28121072 | A | G | exonic | synonymous |  |
| *LOC_Os04g47380* | *Os04g0561700* | 4_28121192 | C | T | exonic | nonsynonymous |  |
| *LOC_Os04g47380* | *Os04g0561700* | 4_28121206 | A | T | exonic | synonymous |  |
| *LOC_Os04g47380* | *Os04g0561700* | 4_28121919 | T | C | exonic | nonsynonymous |  |
| *LOC_Os04g47380* | *Os04g0561700* | 4_28122087 | G | A | exonic | nonsynonymous |  |
| *LOC_Os04g47380* | *Os04g0561700* | 4_28122369 | T | A | intronic |  |  |
| *LOC_Os04g47380* | *Os04g0561700* | 4_28122407 | G | A | intronic |  |  |
| *LOC_Os04g47390* | *Os04g0561800* | 4_28124282 | C | A | intronic |  | GDSL-like lipase/acylhydrolase, putative, expressed |
| *LOC_Os04g47400* | *Os04g0561900* | 4_28127265 | A | C | UTR3 |  | monocopper oxidase, putative, expressed |
| *LOC_Os04g47400* | *Os04g0561900* | 4_28128433 | C | G | exonic | synonymous |  |
| *LOC_Os04g47400* | *Os04g0561900* | 4_28128550 | A | G | exonic | synonymous |  |
| *LOC_Os04g47400* | *Os04g0561900* | 4_28129196 | C | T | intronic |  |  |
| *LOC_Os04g47400* | *Os04g0561900* | 4_28129385 | G | T | intronic |  |  |
| *LOC_Os04g47400* | *Os04g0561900* | 4_28129390 | T | C | intronic |  |  |
| *LOC_Os04g47400* | *Os04g0561900* | 4_28129544 | A | G | intronic |  |  |
| *LOC_Os04g47400* | *Os04g0561900* | 4_28129738 | T | G | intronic |  |  |
| *LOC_Os04g47400* | *Os04g0561900* | 4_28129943 | T | C | intronic |  |  |
| *LOC_Os04g47400* | *Os04g0561900* | 4_28129949 | A | C | intronic |  |  |
| *LOC_Os04g47400* | *Os04g0561900* | 4_28130223 | G | A | UTR5 |  |  |
| *LOC_Os04g47410* | *Os04g0562000* | 4_28131736 | T | G | intronic |  | DHHC zinc finger domain containing protein, expressed |
| *LOC_Os04g47410* | *Os04g0562000* | 4_28132085 | T | G | intronic |  |  |
| *LOC_Os04g47410* | *Os04g0562000* | 4_28132172 | G | T | intronic |  |  |
| *LOC_Os04g47410* | *Os04g0562000* | 4_28132177 | T | A | intronic |  |  |
| *LOC_Os04g47410* | *Os04g0562000* | 4_28132238 | A | G | intronic |  |  |
| *LOC_Os04g47410* | *Os04g0562000* | 4_28132255 | G | A | intronic |  |  |
| *LOC_Os04g47410* | *Os04g0562000* | 4_28132975 | G | A | intronic |  |  |
| *LOC_Os04g47410* | *Os04g0562000* | 4_28133191 | T | G | exonic | nonsynonymous |  |
| *LOC_Os04g47410* | *Os04g0562000* | 4_28133515 | T | G | intronic |  |  |
| *LOC_Os04g47410* | *Os04g0562000* | 4_28134732 | C | G | intronic |  |  |
| *LOC_Os04g47410* | *Os04g0562000* | 4_28135060 | A | G | intronic |  |  |
| *LOC_Os04g47410* | *Os04g0562000* | 4_28135712 | T | C | intronic |  |  |
| *LOC_Os04g47410* | *Os04g0562000* | 4_28135762 | G | A | intronic |  |  |
| *LOC_Os04g47410* | *Os04g0562000* | 4_28135810 | A | G | intronic |  |  |
| *LOC_Os04g47410* | *Os04g0562000* | 4_28136457 | A | C | UTR3 |  |  |
| *LOC_Os04g47410* | *Os04g0562000* | 4_28136566 | T | C | downstream |  |  |
| *LOC_Os04g47410* | *Os04g0562000* | 4_28136617 | G | T | downstream |  |  |
| *LOC_Os04g47410* | *Os04g0562000* | 4_28136954 | A | G | downstream |  |  |
| *LOC_Os04g47410* | *Os04g0562000* | 4_28136964 | A | T | downstream |  |  |
| *LOC_Os04g47410* | *Os04g0562000* | 4_28137053 | G | A | downstream |  |  |
| *LOC_Os04g47410* | *Os04g0562000* | 4_28137059 | T | C | downstream |  |  |
| *LOC_Os04g47410* | *Os04g0562000* | 4_28137286 | C | T | downstream |  |  |
| *LOC_Os04g47410* | *Os04g0562000* | 4_28137374 | C | T | downstream |  |  |
| *LOC_Os04g47410* | *Os04g0562000* | 4_28137414 | T | C | downstream |  |  |
| *LOC_Os04g47410* | *Os04g0562000* | 4_28136954 | A | G | downstream |  |  |
| *LOC_Os04g47410* | *Os04g0562000* | 4_28136964 | A | T | downstream |  |  |
| *LOC_Os04g47410* | *Os04g0562000* | 4_28137053 | G | A | downstream |  |  |
| *LOC_Os04g47410* | *Os04g0562000* | 4_28137059 | T | C | downstream |  |  |
| *LOC_Os04g47410* | *Os04g0562000* | 4_28137286 | C | T | downstream |  |  |
| *LOC_Os04g47410* | *Os04g0562000* | 4_28137374 | C | T | downstream |  |  |
| *LOC_Os04g47410* | *Os04g0562000* | 4_28137414 | T | C | downstream |  |  |
| *LOC_Os04g47420* | *Os04g0562100* | 4_28138295 | A | G | exonic | synonymous | transmembrane amino acid transporter protein, putative, expressed |
| *LOC_Os04g47420* | *Os04g0562100* | 4_28138793 | T | C | exonic | nonsynonymous |  |
| *LOC_Os04g47420* | *Os04g0562100* | 4_28138899 | G | T | exonic | synonymous |  |
| *LOC_Os04g47420* | *Os04g0562100* | 4_28139376 | A | G | intronic |  |  |
| *LOC_Os04g47420* | *Os04g0562100* | 4_28140009 | C | A | upstream |  |  |
| *LOC_Os04g47420* | *Os04g0562100* | 4_28140202 | C | T | upstream |  |  |
| *LOC_Os04g47420* | *Os04g0562100* | 4_28140236 | A | G | upstream |  |  |
| *LOC_Os04g47420* | *Os04g0562100* | 4_28140308 | C | T | upstream |  |  |
| *LOC_Os04g47420* | *Os04g0562100* | 4_28140432 | C | T | upstream |  |  |
| *LOC_Os04g47420* | *Os04g0562100* | 4_28140729 | G | A | upstream |  |  |
| *LOC_Os04g47420* | *Os04g0562100* | 4_28140843 | C | T | upstream |  |  |
| *LOC_Os04g47420* | *Os04g0562100* | 4_28140857 | G | A | upstream |  |  |
| *LOC_Os04g47480* | *Os04g0562800* | 4_28171610 | T | C | exonic | nonsynonymous | DUF630/DUF632 domains containing protein, putative, expressed |
| *LOC_Os04g47480* | *Os04g0562800* | 4_28172792 | T | G | intronic |  |  |
| *LOC_Os04g47480* | *Os04g0562800* | 4_28173151 | C | G | exonic | nonsynonymous |  |
| *LOC_Os04g47480* | *Os04g0562800* | 4_28174446 | C | T | downstream |  |  |
| *LOC_Os04g47480* | *Os04g0562800* | 4_28174631 | T | G | downstream |  |  |
| *LOC_Os04g47480* | *Os04g0562800* | 4_28174713 | T | A | downstream |  |  |
| *LOC_Os04g47480* | *Os04g0562800* | 4_28174766 | A | G | downstream |  |  |
| *LOC_Os04g47480* | *Os04g0562800* | 4_28175028 | A | G | downstream |  |  |
| *LOC_Os04g47480* | *Os04g0562800* | 4_28175125 | G | C | downstream |  |  |
| *LOC_Os04g47480* | *Os04g0562800* | 4_28175185 | G | T | downstream |  |  |
| *LOC_Os04g47480* | *Os04g0562800* | 4_28175270 | A | C | downstream |  |  |
| *LOC_Os04g47520* | *Os04g0563000* | 4_28181643 | C | G | intronic |  | growth regulator related protein, putative, expressed |
| *LOC_Os04g47520* | *Os04g0563000* | 4_28181673 | A | C | intronic |  |  |
| *LOC_Os04g47520* | *Os04g0563000* | 4_28181736 | C | A | intronic |  |  |
| *LOC_Os04g47520* | *Os04g0563000* | 4_28181737 | A | C | intronic |  |  |
| *LOC_Os04g47520* | *Os04g0563000* | 4_28182502 | G | A | exonic | synonymous |  |
| *LOC_Os04g47520* | *Os04g0563000* | 4_28183207 | C | T | exonic | synonymous |  |
| *LOC_Os04g47520* | *Os04g0563000* | 4_28183268 | G | A | intronic |  |  |
| *LOC_Os04g47520* | *Os04g0563000* | 4_28183348 | A | G | exonic | synonymous |  |
| *LOC_Os04g47520* | *Os04g0563000* | 4_28183933 | T | G | intronic |  |  |
| *LOC_Os04g47520* | *Os04g0563000* | 4_28184205 | C | T | intronic |  |  |
| *LOC_Os04g47520* | *Os04g0563000* | 4_28184627 | G | A | exonic | synonymous |  |
| *LOC_Os04g47520* | *Os04g0563000* | 4_28184729 | T | C | intronic |  |  |
| *LOC_Os04g47530* | *Os04g0563100* | 4_28191535 | C | T | downstream |  | transmembrane protein, putative, expressed |
| *LOC_Os04g47530* | *Os04g0563100* | 4_28191942 | T | A | downstream |  |  |
| *LOC_Os04g47530* | *Os04g0563100* | 4_28192207 | C | A | downstream |  |  |
| *LOC_Os04g47530* | *Os04g0563100* | 4_28192247 | A | T | downstream |  |  |
| *LOC_Os04g47530* | *Os04g0563100* | 4_28192939 | T | A | intronic |  |  |
| *LOC_Os04g47530* | *Os04g0563100* | 4_28193076 | T | G | exonic | nonsynonymous |  |
| *LOC_Os04g47530* | *Os04g0563100* | 4_28193244 | G | A | intronic |  |  |
| *LOC_Os04g47530* | *Os04g0563100* | 4_28193327 | G | A | intronic |  |  |
| *LOC_Os04g47530* | *Os04g0563100* | 4_28193341 | C | T | intronic |  |  |
| *LOC_Os04g47530* | *Os04g0563100* | 4_28194169 | A | G | exonic | synonymous |  |
| *LOC_Os04g47530* | *Os04g0563100* | 4_28194352 | A | C | intronic |  |  |
| *LOC_Os04g47530* | *Os04g0563100* | 4_28194632 | G | A | intronic |  |  |
| *LOC_Os04g47530* | *Os04g0563100* | 4_28194916 | C | T | exonic | synonymous |  |
| *LOC_Os04g47530* | *Os04g0563100* | 4_28195021 | A | G | exonic | synonymous |  |
| *LOC_Os04g47530* | *Os04g0563100* | 4_28195231 | C | T | exonic | nonsynonymous |  |
| *LOC_Os04g47530* | *Os04g0563100* | 4_28195339 | A | G | UTR5 |  |  |
| *LOC_Os04g47530* | *Os04g0563100* | 4_28195340 | T | C | UTR5 |  |  |
| *LOC_Os04g47580* | *Os04g0563700* | 4_28218391 | A | T | downstream |  | cyclin, putative, expressed |
| *LOC_Os04g47580* | *Os04g0563700* | 4_28218456 | C | A | downstream |  |  |
| *LOC_Os04g47580* | *Os04g0563700* | 4_28218564 | A | T | downstream |  |  |
| *LOC_Os04g47580* | *Os04g0563700* | 4_28218597 | A | G | downstream |  |  |
| *LOC_Os04g47580* | *Os04g0563700* | 4_28218643 | C | A | downstream |  |  |
| *LOC_Os04g47580* | *Os04g0563700* | 4_28218696 | T | G | downstream |  |  |
| *LOC_Os04g47580* | *Os04g0563700* | 4_28219058 | G | T | exonic | nonsynonymous |  |
| *LOC_Os04g47580* | *Os04g0563700* | 4_28219176 | C | G | exonic | synonymous |  |
| *LOC_Os04g47580* | *Os04g0563700* | 4_28220143 | C | T | exonic | synonymous |  |
| *LOC_Os04g47580* | *Os04g0563700* | 4_28220852 | A | C | exonic | nonsynonymous |  |
| *LOC_Os04g47580* | *Os04g0563700* | 4_28221080 | G | A | intronic |  |  |
| *LOC_Os04g47580* | *Os04g0563700* | 4_28221096 | A | G | intronic |  |  |
| *LOC_Os04g47580* | *Os04g0563700* | 4_28221310 | G | T | intronic |  |  |
| *LOC_Os04g47580* | *Os04g0563700* | 4_28222107 | T | A | upstream |  |  |
| *LOC_Os04g47580* | *Os04g0563700* | 4_28222243 | G | A | upstream |  |  |
| *LOC_Os04g47590* | *Os04g0563801* | 4_28223906 | T | A | downstream |  | niemann-Pick C1 protein precursor, putative, expressed |
| *LOC_Os04g47590* | *Os04g0563801* | 4_28223980 | A | C | downstream |  |  |
| *LOC_Os04g47590* | *Os04g0563801* | 4_28224030 | G | C | downstream |  |  |
| *LOC_Os04g47590* | *Os04g0563801* | 4_28224107 | G | A | downstream |  |  |
| *LOC_Os04g47590* | *Os04g0563801* | 4_28224265 | G | A | downstream |  |  |
| *LOC_Os04g47590* | *Os04g0563801* | 4_28224287 | A | G | downstream |  |  |
| *LOC_Os04g47590* | *Os04g0563801* | 4_28224288 | G | A | downstream |  |  |
| *LOC_Os04g47590* | *Os04g0563801* | 4_28224317 | G | A | downstream |  |  |
| *LOC_Os04g47590* | *Os04g0563801* | 4_28224593 | T | C | downstream |  |  |
| *LOC_Os04g47590* | *Os04g0563801* | 4_28224918 | A | C | exonic | nonsynonymous |  |
| *LOC_Os04g47590* | *Os04g0563801* | 4_28225944 | A | C | intronic |  |  |
| *LOC_Os04g47590* | *Os04g0563801* | 4_28226297 | T | A | intronic |  |  |
| *LOC_Os04g47590* | *Os04g0563801* | 4_28226773 | A | G | intronic |  |  |
| *LOC_Os04g47590* | *Os04g0563801* | 4_28226922 | T | C | exonic | synonymous |  |
| *LOC_Os04g47590* | *Os04g0563801* | 4_28227063 | A | C | exonic | synonymous |  |
| *LOC_Os04g47590* | *Os04g0563801* | 4_28227449 | T | G | intronic |  |  |
| *LOC_Os04g47590* | *Os04g0563801* | 4_28227510 | A | G | intronic |  |  |
| *LOC_Os04g47590* | *Os04g0563801* | 4_28227661 | G | C | intronic |  |  |
| *LOC_Os04g47590* | *Os04g0563801* | 4_28228028 | A | G | intronic |  |  |
| *LOC_Os04g47590* | *Os04g0563801* | 4_28228137 | T | C | exonic | synonymous |  |
| *LOC_Os04g47590* | *Os04g0563801* | 4_28228430 | C | A | exonic | nonsynonymous |  |
| *LOC_Os04g47590* | *Os04g0563801* | 4_28228434 | A | G | exonic | nonsynonymous |  |
| *LOC_Os04g47590* | *Os04g0563801* | 4_28229158 | G | A | intronic |  |  |
| *LOC_Os04g47590* | *Os04g0563801* | 4_28229772 | C | A | intronic |  |  |
| *LOC_Os04g47590* | *Os04g0563801* | 4_28230606 | T | C | intronic |  |  |
| *LOC_Os04g47590* | *Os04g0563801* | 4_28231423 | A | C | intronic |  |  |
| *LOC_Os04g47590* | *Os04g0563801* | 4_28231511 | C | T | intronic |  |  |
| *LOC_Os04g47590* | *Os04g0563801* | 4_28231732 | C | G | intronic |  |  |
| *LOC_Os04g47590* | *Os04g0563801* | 4_28232104 | A | C | exonic |  |  |
| *LOC_Os04g47590* | *Os04g0563801* | 4_28232543 | A | G | intronic |  |  |
| *LOC_Os04g47590* | *Os04g0563801* | 4_28232774 | A | G | intronic |  |  |
| *LOC_Os04g47590* | *Os04g0563801* | 4_28232876 | G | T | intronic |  |  |
| *LOC_Os04g47590* | *Os04g0563801* | 4_28233451 | G | A | intronic |  |  |
| *LOC_Os04g47590* | *Os04g0563801* | 4_28233656 | A | G | intronic |  |  |
| *LOC_Os04g47590* | *Os04g0563801* | 4_28233748 | T | C | exonic |  |  |
| *LOC_Os04g47590* | *Os04g0563801* | 4_28233798 | T | C | exonic |  |  |
| *LOC_Os04g47590* | *Os04g0563801* | 4_28233830 | A | G | intronic |  |  |
| *LOC_Os04g47590* | *Os04g0563801* | 4_28234252 | C | T | intronic |  |  |
| *LOC_Os04g47590* | *Os04g0563801* | 4_28234313 | T | C | intronic |  |  |
| *LOC_Os04g47590* | *Os04g0563801* | 4_28234567 | G | A | intronic |  |  |
| *LOC_Os04g47590* | *Os04g0563801* | 4_28234614 | T | C | intronic |  |  |
| *LOC_Os04g47590* | *Os04g0563801* | 4_28234819 | A | G | intronic |  |  |
| *LOC_Os04g47590* | *Os04g0563801* | 4_28235048 | G | A | intronic |  |  |
| *LOC_Os04g47590* | *Os04g0563801* | 4_28235471 | C | A | upstream |  |  |
| *LOC_Os04g47590* | *Os04g0563801* | 4_28235556 | A | C | upstream |  |  |
| *LOC_Os04g47590* | *Os04g0563801* | 4_28235873 | A | T | upstream |  |  |
| *LOC_Os04g47620* | *Os04g0563900* | 4_28261962 | C | T | downstream |  | protein kinase APK1B, chloroplast precursor, putative, expressed |
| *LOC_Os04g47620* | *Os04g0563900* | 4_28263400 | C | T | exonic |  |  |
| *LOC_Os04g47620* | *Os04g0563900* | 4_28263878 | A | G | exonic |  |  |
| *LOC_Os04g47620* | *Os04g0563900* | 4_28266732 | T | A | upstream |  |  |
| *LOC_Os04g47620* | *Os04g0563900* | 4_28266735 | T | C | upstream |  |  |
| *LOC_Os04g47640* | *Os04g0564000* | 4_28272569 | T | C | downstream |  | RWP-RK domain-containing protein, putative, expressed |
| *LOC_Os04g47640* | *Os04g0564000* | 4_28272570 | C | T | downstream |  |  |
| *LOC_Os04g47640* | *Os04g0564000* | 4_28272609 | C | T | downstream |  |  |
| *LOC_Os04g47640* | *Os04g0564000* | 4_28272785 | T | C | downstream |  |  |
| *LOC_Os04g47640* | *Os04g0564000* | 4_28272893 | A | C | downstream |  |  |
| *LOC_Os04g47640* | *Os04g0564000* | 4_28272976 | T | C | downstream |  |  |
| *LOC_Os04g47640* | *Os04g0564000* | 4_28273136 | C | T | downstream |  |  |
| *LOC_Os04g47640* | *Os04g0564000* | 4_28273173 | G | A | downstream |  |  |
| *LOC_Os04g47640* | *Os04g0564000* | 4_28273210 | C | A | downstream |  |  |
| *LOC_Os04g47640* | *Os04g0564000* | 4_28273639 | G | A | exonic |  |  |
| *LOC_Os04g47640* | *Os04g0564000* | 4_28273903 | A | G | exonic | synonymous |  |
| *LOC_Os04g47640* | *Os04g0564000* | 4_28274367 | A | G | intronic |  |  |
| *LOC_Os04g47640* | *Os04g0564000* | 4_28274458 | G | A | intronic |  |  |
| *LOC_Os04g47640* | *Os04g0564000* | 4_28275914 | T | C | upstream |  |  |
| *LOC_Os04g47640* | *Os04g0564000* | 4_28275992 | T | G | upstream |  |  |
| *LOC_Os04g47640* | *Os04g0564000* | 4_28276671 | C | T | upstream |  |  |
| *LOC_Os04g47680* | *Os04g0564500* | 4_28289331 | T | G | upstream |  | Ser/Thr-rich protein T10 in DGCR region, putative, expressed |
| *LOC_Os04g47680* | *Os04g0564500* | 4_28289332 | T | C | upstream |  |  |
| *LOC_Os04g47680* | *Os04g0564500* | 4_28289403 | G | A | upstream |  |  |
| *LOC_Os04g47680* | *Os04g0564500* | 4_28289448 | G | T | upstream |  |  |
| *LOC_Os04g47680* | *Os04g0564500* | 4_28289656 | A | C | upstream |  |  |
| *LOC_Os04g47680* | *Os04g0564500* | 4_28289707 | C | T | upstream |  |  |
| *LOC_Os04g47680* | *Os04g0564500* | 4_28290417 | T | G | exonic | nonsynonymous |  |
| *LOC_Os04g47680* | *Os04g0564500* | 4_28290541 | T | C | intronic |  |  |
| *LOC_Os04g47680* | *Os04g0564500* | 4_28290978 | T | C | intronic |  |  |
| *LOC_Os04g47680* | *Os04g0564500* | 4_28291188 | T | A | exonic | synonymous |  |
| *LOC_Os04g47680* | *Os04g0564500* | 4_28291238 | G | T | intronic |  |  |
| *LOC_Os04g47680* | *Os04g0564500* | 4_28292012 | A | T | UTR3 |  |  |
| *LOC_Os04g47680* | *Os04g0564500* | 4_28292057 | T | C | UTR3 |  |  |
| *LOC_Os04g47690* | *Os04g0564600* | 4_28293613 | G | C | intronic |  | HMG1/2, putative, expressed |
| *LOC_Os04g47690* | *Os04g0564600* | 4_28293657 | T | A | intronic |  |  |
| *LOC_Os04g47690* | *Os04g0564600* | 4_28294311 | A | G | UTR3 |  |  |
| *LOC_Os04g47690* | *Os04g0564600* | 4_28294328 | A | G | UTR3 |  |  |
| *LOC_Os04g47690* | *Os04g0564600* | 4_28294481 | T | C | intronic |  |  |
| *LOC_Os04g47690* | *Os04g0564600* | 4_28294495 | T | C | intronic |  |  |
| *LOC_Os04g47690* | *Os04g0564600* | 4_28294619 | G | C | intronic |  |  |
| *LOC_Os04g47690* | *Os04g0564600* | 4_28294730 | T | G | intronic |  |  |
| *LOC_Os04g47690* | *Os04g0564600* | 4_28294768 | C | T | intronic |  |  |
| *LOC_Os04g47690* | *Os04g0564600* | 4_28294806 | G | A | intronic |  |  |
| *LOC_Os04g47690* | *Os04g0564600* | 4_28295044 | G | A | intronic |  |  |
| *LOC_Os04g47690* | *Os04g0564600* | 4_28295675 | A | C | intronic |  |  |
| *LOC_Os04g47690* | *Os04g0564600* | 4_28295745 | C | T | intronic |  |  |
| *LOC_Os04g47690* | *Os04g0564600* | 4_28295952 | T | C | UTR3 |  |  |
| *LOC_Os04g47690* | *Os04g0564600* | 4_28296230 | C | T | downstream |  |  |
| *LOC_Os04g47690* | *Os04g0564600* | 4_28296231 | A | C | downstream |  |  |
| *LOC_Os04g47690* | *Os04g0564600* | 4_28296384 | C | T | downstream |  |  |
| *LOC_Os04g47720* | *Os04g0565200* | 4_28314084 | T | A | upstream |  | cis-zeatin O-glucosyltransferase, putative, expressed |
| *LOC_Os04g47720* | *Os04g0565200* | 4_28314656 | G | A | upstream |  |  |
| *LOC_Os04g47720* | *Os04g0565200* | 4_28314665 | G | A | upstream |  |  |
| *LOC_Os04g47720* | *Os04g0565200* | 4_28314793 | C | G | upstream |  |  |
| *LOC_Os04g47720* | *Os04g0565200* | 4_28314826 | C | T | upstream |  |  |
| *LOC_Os04g47720* | *Os04g0565200* | 4_28314875 | A | T | upstream |  |  |
| *LOC_Os04g47720* | *Os04g0565200* | 4_28314984 | T | G | upstream |  |  |
| *LOC_Os04g47720* | *Os04g0565200* | 4_28316482 | A | G | exonic | synonymous |  |
| *LOC_Os04g47720* | *Os04g0565200* | 4_28316906 | A | C | downstream |  |  |
| *LOC_Os04g47720* | *Os04g0565200* | 4_28316929 | T | C | downstream |  |  |
| *LOC_Os04g47720* | *Os04g0565200* | 4_28316993 | T | C | downstream |  |  |
| *LOC_Os04g47720* | *Os04g0565200* | 4_28317011 | C | T | downstream |  |  |
| *LOC_Os04g47770* | *Os04g0565400* | 4_28335972 | C | T | upstream |  | cis-zeatin O-glucosyltransferase, putative, expressed |
| *LOC_Os04g47770* | *Os04g0565400* | 4_28336011 | G | A | upstream |  |  |
| *LOC_Os04g47770* | *Os04g0565400* | 4_28336067 | G | A | upstream |  |  |
| *LOC_Os04g47770* | *Os04g0565400* | 4_28336149 | C | T | upstream |  |  |
| *LOC_Os04g47770* | *Os04g0565400* | 4_28336215 | T | C | upstream |  |  |
| *LOC_Os04g47770* | *Os04g0565400* | 4_28336234 | C | T | upstream |  |  |
| *LOC_Os04g47770* | *Os04g0565400* | 4_28336253 | T | C | upstream |  |  |
| *LOC_Os04g47770* | *Os04g0565400* | 4_28336284 | G | A | upstream |  |  |
| *LOC_Os04g47770* | *Os04g0565400* | 4_28336456 | G | A | upstream |  |  |
| *LOC_Os04g47770* | *Os04g0565400* | 4_28336637 | A | C | UTR5 |  |  |
| *LOC_Os04g47770* | *Os04g0565400* | 4_28336644 | C | T | UTR5 |  |  |
| *LOC_Os04g47770* | *Os04g0565400* | 4_28337946 | T | G | exonic | nonsynonymous |  |
| *LOC_Os04g47770* | *Os04g0565400* | 4_28338430 | C | T | downstream |  |  |
| *LOC_Os04g47770* | *Os04g0565400* | 4_28338502 | G | A | downstream |  |  |
| *LOC_Os04g47770* | *Os04g0565400* | 4_28338944 | G | A | downstream |  |  |
| *LOC_Os04g47780* | *Os04g0565500* | 4_28340997 | T | G | downstream |  | transmembrane amino acid transporter protein, putative, expressed |
| *LOC_Os04g47780* | *Os04g0565500* | 4_28341202 | A | C | downstream |  |  |
| *LOC_Os04g47780* | *Os04g0565500* | 4_28341522 | G | A | downstream |  |  |
| *LOC_Os04g47780* | *Os04g0565500* | 4_28341545 | C | A | downstream |  |  |
| *LOC_Os04g47780* | *Os04g0565500* | 4_28341697 | C | T | downstream |  |  |
| *LOC_Os04g47780* | *Os04g0565500* | 4_28342253 | T | C | exonic | nonsynonymous |  |
| *LOC_Os04g47780* | *Os04g0565500* | 4_28343502 | G | C | upstream |  |  |
| *LOC_Os04g47780* | *Os04g0565500* | 4_28343536 | A | G | upstream |  |  |
| *LOC_Os04g47780* | *Os04g0565500* | 4_28343708 | T | G | upstream |  |  |
| *LOC_Os04g47780* | *Os04g0565500* | 4_28343748 | G | T | upstream |  |  |
| *LOC_Os04g47780* | *Os04g0565500* | 4_28343790 | C | T | upstream |  |  |
| *LOC_Os04g47810* | *Os04g0565900* | 4_28369064 | A | G | downstream |  | ethylene-responsive protein related, putative, expressed |
| *LOC_Os04g47810* | *Os04g0565900* | 4_28369221 | A | C | downstream |  |  |
| *LOC_Os04g47810* | *Os04g0565900* | 4_28369264 | G | A | downstream |  |  |
| *LOC_Os04g47810* | *Os04g0565900* | 4_28369362 | C | T | downstream |  |  |
| *LOC_Os04g47810* | *Os04g0565900* | 4_28369373 | A | G | UTR3 |  |  |
| *LOC_Os04g47810* | *Os04g0565900* | 4_28369446 | C | T | UTR3 |  |  |
| *LOC_Os04g47810* | *Os04g0565900* | 4_28369525 | A | G | UTR3 |  |  |
| *LOC_Os04g47810* | *Os04g0565900* | 4_28369534 | T | C | UTR3 |  |  |
| *LOC_Os04g47810* | *Os04g0565900* | 4_28370135 | G | A | intronic |  |  |
| *LOC_Os04g47810* | *Os04g0565900* | 4_28370381 | C | T | intronic |  |  |
| *LOC_Os04g47810* | *Os04g0565900* | 4_28370718 | C | T | intronic |  |  |
| *LOC_Os04g47810* | *Os04g0565900* | 4_28370762 | C | T | exonic | synonymous |  |
| *LOC_Os04g47810* | *Os04g0565900* | 4_28370892 | T | C | intronic |  |  |
| *LOC_Os04g47810* | *Os04g0565900* | 4_28371008 | A | G | exonic | nonsynonymous |  |
| *LOC_Os04g47810* | *Os04g0565900* | 4_28371055 | G | C | exonic | nonsynonymous |  |
| *LOC_Os04g47810* | *Os04g0565900* | 4_28371257 | T | C | exonic | nonsynonymous |  |
| *LOC_Os04g47810* | *Os04g0565900* | 4_28371617 | T | C | exonic | nonsynonymous |  |
| *LOC_Os04g47810* | *Os04g0565900* | 4_28371768 | T | G | exonic | nonsynonymous |  |
| *LOC_Os04g47810* | *Os04g0565900* | 4_28372068 | A | T | upstream |  |  |
| *LOC_Os04g47810* | *Os04g0565900* | 4_28372335 | G | A | upstream |  |  |
| *LOC_Os04g47810* | *Os04g0565900* | 4_28372337 | A | G | upstream |  |  |
| *LOC_Os04g47810* | *Os04g0565900* | 4_28372355 | G | A | upstream |  |  |
| *LOC_Os04g47810* | *Os04g0565900* | 4_28372525 | C | T | upstream |  |  |
| *LOC_Os04g47810* | *Os04g0565900* | 4_28372605 | A | C | upstream |  |  |
| *LOC_Os04g47810* | *Os04g0565900* | 4_28372676 | C | A | upstream |  |  |
| *LOC_Os04g47810* | *Os04g0565900* | 4_28372694 | C | T | upstream |  |  |
| *LOC_Os04g47810* | *Os04g0565900* | 4_28372712 | T | C | upstream |  |  |
| *LOC_Os04g47830* | *Os04g0566100* | 4_28378547 | G | A | upstream |  | SNF2 family N-terminal domain containing protein, expressed |
| *LOC_Os04g47830* | *Os04g0566100* | 4_28378563 | C | T | upstream |  |  |
| *LOC_Os04g47830* | *Os04g0566100* | 4_28378638 | A | G | upstream |  |  |
| *LOC_Os04g47830* | *Os04g0566100* | 4_28378810 | G | A | upstream |  |  |
| *LOC_Os04g47830* | *Os04g0566100* | 4_28378822 | T | C | upstream |  |  |
| *LOC_Os04g47830* | *Os04g0566100* | 4_28378946 | A | C | upstream |  |  |
| *LOC_Os04g47830* | *Os04g0566100* | 4_28378997 | T | C | upstream |  |  |
| *LOC_Os04g47830* | *Os04g0566100* | 4_28379115 | A | G | upstream |  |  |
| *LOC_Os04g47830* | *Os04g0566100* | 4_28379180 | G | A | upstream |  |  |
| *LOC_Os04g47830* | *Os04g0566100* | 4_28379208 | G | A | upstream |  |  |
| *LOC_Os04g47830* | *Os04g0566100* | 4_28379255 | T | G | upstream |  |  |
| *LOC_Os04g47830* | *Os04g0566100* | 4_28379307 | G | A | upstream |  |  |
| *LOC_Os04g47830* | *Os04g0566100* | 4_28379331 | C | T | upstream |  |  |
| *LOC_Os04g47830* | *Os04g0566100* | 4_28379420 | T | A | upstream |  |  |
| *LOC_Os04g47830* | *Os04g0566100* | 4_28380156 | C | G | exonic | synonymous |  |
| *LOC_Os04g47830* | *Os04g0566100* | 4_28380306 | G | A | exonic | synonymous |  |
| *LOC_Os04g47830* | *Os04g0566100* | 4_28380839 | C | T | intronic |  |  |
| *LOC_Os04g47830* | *Os04g0566100* | 4_28380962 | T | A | intronic |  |  |
| *LOC_Os04g47830* | *Os04g0566100* | 4_28381616 | A | T | intronic |  |  |
| *LOC_Os04g47830* | *Os04g0566100* | 4_28381641 | A | G | intronic |  |  |
| *LOC_Os04g47830* | *Os04g0566100* | 4_28382257 | T | G | exonic | nonsynonymous |  |
| *LOC_Os04g47830* | *Os04g0566100* | 4_28382505 | T | C | intronic |  |  |
| *LOC_Os04g47830* | *Os04g0566100* | 4_28382647 | G | A | intronic |  |  |
| *LOC_Os04g47830* | *Os04g0566100* | 4_28382706 | T | A | intronic |  |  |
| *LOC_Os04g47830* | *Os04g0566100* | 4_28382823 | C | T | intronic |  |  |
| *LOC_Os04g47830* | *Os04g0566100* | 4_28383106 | G | T | intronic |  |  |
| *LOC_Os04g47830* | *Os04g0566100* | 4_28383209 | C | T | exonic | synonymous |  |
| *LOC_Os04g47830* | *Os04g0566100* | 4_28383385 | A | C | intronic |  |  |
| *LOC_Os04g47830* | *Os04g0566100* | 4_28383539 | T | G | intronic |  |  |
| *LOC_Os04g47830* | *Os04g0566100* | 4_28384106 | T | A | intronic |  |  |
| *LOC_Os04g47830* | *Os04g0566100* | 4_28384134 | G | C | intronic |  |  |
| *LOC_Os04g47830* | *Os04g0566100* | 4_28384564 | C | A | exonic | nonsynonymous |  |
| *LOC_Os04g47830* | *Os04g0566100* | 4_28385143 | A | T | UTR3 |  |  |
| *LOC_Os04g47830* | *Os04g0566100* | 4_28385198 | A | G | UTR3 |  |  |
| *LOC_Os04g47830* | *Os04g0566100* | 4_28385569 | T | G | downstream |  |  |
| *LOC_Os04g47830* | *Os04g0566100* | 4_28385886 | A | T | downstream |  |  |
| *LOC_Os04g47830* | *Os04g0566100* | 4_28385889 | T | G | downstream |  |  |
| *LOC_Os04g47830* | *Os04g0566100* | 4_28386322 | G | A | downstream |  |  |
| *LOC_Os04g47830* | *Os04g0566100* | 4_28386323 | T | C | downstream |  |  |
| *LOC_Os04g47830* | *Os04g0566100* | 4_28386421 | T | C | downstream |  |  |
| *LOC_Os04g47860* | *Os04g0566400* | 4_28407959 | G | A | upstream |  | ZOS4-11 - C2H2 zinc finger protein, expressed |
| *LOC_Os04g47860* | *Os04g0566400* | 4_28408039 | G | C | upstream |  |  |
| *LOC_Os04g47860* | *Os04g0566400* | 4_28408240 | C | T | upstream |  |  |
| *LOC_Os04g47860* | *Os04g0566400* | 4_28408404 | A | G | upstream |  |  |
| *LOC_Os04g47860* | *Os04g0566400* | 4_28408538 | C | T | upstream |  |  |
| *LOC_Os04g47860* | *Os04g0566400* | 4_28409560 | C | A | UTR5 |  |  |
| *LOC_Os04g47860* | *Os04g0566400* | 4_28409972 | T | G | exonic | nonsynonymous |  |
| *LOC_Os04g47860* | *Os04g0566400* | 4_28410058 | T | C | UTR3 |  |  |
| *LOC_Os04g47860* | *Os04g0566400* | 4_28410332 | A | G | UTR3 |  |  |
| *LOC_Os04g47860* | *Os04g0566400* | 4_28411059 | G | T | intronic |  |  |
| *LOC_Os04g47860* | *Os04g0566400* | 4_28411182 | A | G | intronic |  |  |
| *LOC_Os04g47860* | *Os04g0566400* | 4_28412052 | T | C | intronic |  |  |
| *LOC_Os04g47860* | *Os04g0566400* | 4_28412327 | A | G | intronic |  |  |
| *LOC_Os04g47860* | *Os04g0566400* | 4_28412405 | A | T | intronic |  |  |
| *LOC_Os04g47860* | *Os04g0566400* | 4_28412710 | G | A | intronic |  |  |
| *LOC_Os04g47860* | *Os04g0566400* | 4_28412873 | G | A | intronic |  |  |
| *LOC_Os04g47860* | *Os04g0566400* | 4_28413031 | T | A | intronic |  |  |
| *LOC_Os04g47860* | *Os04g0566400* | 4_28413207 | A | T | intronic |  |  |
| *LOC_Os04g47860* | *Os04g0566400* | 4_28413264 | A | T | intronic |  |  |
| *LOC_Os04g47860* | *Os04g0566400* | 4_28413302 | T | C | intronic |  |  |
| *LOC_Os04g47860* | *Os04g0566400* | 4_28413385 | G | A | intronic |  |  |
| *LOC_Os04g47860* | *Os04g0566400* | 4_28413772 | A | G | intronic |  |  |
| *LOC_Os04g47860* | *Os04g0566400* | 4_28413888 | C | G | intronic |  |  |
| *LOC_Os04g47860* | *Os04g0566400* | 4_28415727 | G | A | UTR3 |  |  |
| *LOC_Os04g47860* | *Os04g0566400* | 4_28415871 | T | G | UTR3 |  |  |
| *LOC_Os04g47860* | *Os04g0566400* | 4_28415886 | C | A | UTR3 |  |  |
| *LOC_Os04g47860* | *Os04g0566400* | 4_28416637 | T | A | downstream |  |  |
| *LOC_Os04g47860* | *Os04g0566400* | 4_28416717 | A | C | downstream |  |  |
| *LOC_Os04g47860* | *Os04g0566400* | 4_28416757 | C | T | downstream |  |  |
| *LOC_Os04g47860* | *Os04g0566400* | 4_28416772 | A | C | downstream |  |  |
| *LOC_Os04g47860* | *Os04g0566400* | 4_28416871 | A | G | downstream |  |  |
| *LOC_Os04g47860* | *Os04g0566400* | 4_28416888 | G | C | downstream |  |  |
| *LOC_Os04g47860* | *Os04g0566400* | 4_28416927 | C | T | downstream |  |  |
| *LOC_Os04g47870* | *Os04g0566500* | 4_28425651 | C | A | downstream |  | PINHEAD, putative, expressed |
| *LOC_Os04g47870* | *Os04g0566500* | 4_28426729 | G | A | UTR3 |  |  |
| *LOC_Os04g47870* | *Os04g0566500* | 4_28427211 | T | A | intronic |  |  |
| *LOC_Os04g47870* | *Os04g0566500* | 4_28428124 | C | T | intronic |  |  |
| *LOC_Os04g47870* | *Os04g0566500* | 4_28428312 | G | A | intronic |  |  |
| *LOC_Os04g47870* | *Os04g0566500* | 4_28429203 | C | T | intronic |  |  |
| *LOC_Os04g47870* | *Os04g0566500* | 4_28429238 | T | A | intronic |  |  |
| *LOC_Os04g47870* | *Os04g0566500* | 4_28429665 | C | T | intronic |  |  |
| *LOC_Os04g47870* | *Os04g0566500* | 4_28429967 | G | T | intronic |  |  |
| *LOC_Os04g47870* | *Os04g0566500* | 4_28430175 | G | A | intronic |  |  |
| *LOC_Os04g47870* | *Os04g0566500* | 4_28430213 | T | A | intronic |  |  |
| *LOC_Os04g47870* | *Os04g0566500* | 4_28430353 | A | G | intronic |  |  |
| *LOC_Os04g47870* | *Os04g0566500* | 4_28430456 | T | A | intronic |  |  |
| *LOC_Os04g47870* | *Os04g0566500* | 4_28430585 | G | A | intronic |  |  |
| *LOC_Os04g47870* | *Os04g0566500* | 4_28430618 | C | T | intronic |  |  |
| *LOC_Os04g47870* | *Os04g0566500* | 4_28430647 | C | T | intronic |  |  |
| *LOC_Os04g47870* | *Os04g0566500* | 4_28430782 | G | A | intronic |  |  |
| *LOC_Os04g47870* | *Os04g0566500* | 4_28430992 | A | G | exonic | synonymous |  |
| *LOC_Os04g47870* | *Os04g0566500* | 4_28430999 | A | G | exonic | synonymous |  |
| *LOC_Os04g47870* | *Os04g0566500* | 4_28431073 | A | C | intronic |  |  |
| *LOC_Os04g47870* | *Os04g0566500* | 4_28431239 | C | T | exonic | synonymous |  |
| *LOC_Os04g47870* | *Os04g0566500* | 4_28431426 | G | A | intronic |  |  |
| *LOC_Os04g47870* | *Os04g0566500* | 4_28431578 | T | C | intronic |  |  |
| *LOC_Os04g47870* | *Os04g0566500* | 4_28431606 | C | T | intronic |  |  |
| *LOC_Os04g47870* | *Os04g0566500* | 4_28431815 | G | A | intronic |  |  |
| *LOC_Os04g47870* | *Os04g0566500* | 4_28432375 | T | A | exonic | synonymous |  |
| *LOC_Os04g47870* | *Os04g0566500* | 4_28433153 | A | G | exonic | synonymous |  |
| *LOC_Os04g47870* | *Os04g0566500* | 4_28433210 | G | A | exonic | synonymous |  |
| *LOC_Os04g47870* | *Os04g0566500* | 4_28433234 | A | C | exonic | nonsynonymous |  |
| *LOC_Os04g47870* | *Os04g0566500* | 4_28433346 | C | T | exonic | nonsynonymous |  |
| *LOC_Os04g47870* | *Os04g0566500* | 4_28434032 | C | G | intronic |  |  |
| *LOC_Os04g47870* | *Os04g0566500* | 4_28434243 | C | A | intronic |  |  |
| *LOC_Os04g47870* | *Os04g0566500* | 4_28434306 | G | A | intronic |  |  |
| *LOC_Os04g47870* | *Os04g0566500* | 4_28434567 | A | T | intronic |  |  |
| *LOC_Os04g47870* | *Os04g0566500* | 4_28434658 | G | A | intronic |  |  |
| *LOC_Os04g47870* | *Os04g0566500* | 4_28434705 | T | A | intronic |  |  |
| *LOC_Os04g47870* | *Os04g0566500* | 4_28434814 | G | T | intronic |  |  |
| *LOC_Os04g47870* | *Os04g0566500* | 4_28435156 | A | G | intronic |  |  |
| *LOC_Os04g47870* | *Os04g0566500* | 4_28435389 | T | A | intronic |  |  |
| *LOC_Os04g47870* | *Os04g0566500* | 4_28436390 | A | G | intronic |  |  |
| *LOC_Os04g47870* | *Os04g0566500* | 4_28436994 | T | G | intronic |  |  |
| *LOC_Os04g47870* | *Os04g0566500* | 4_28437291 | A | T | intronic |  |  |
| *LOC_Os04g47870* | *Os04g0566500* | 4_28437389 | G | A | intronic |  |  |
| *LOC_Os04g47870* | *Os04g0566500* | 4_28437931 | C | A | intronic |  |  |
| *LOC_Os04g47870* | *Os04g0566500* | 4_28438335 | G | T | intronic |  |  |
| *LOC_Os04g47870* | *Os04g0566500* | 4_28438511 | T | C | intronic |  |  |
| *LOC_Os04g47870* | *Os04g0566500* | 4_28438753 | A | G | intronic |  |  |
| *LOC_Os04g47870* | *Os04g0566500* | 4_28438774 | C | T | intronic |  |  |
| *LOC_Os04g47870* | *Os04g0566500* | 4_28438910 | G | A | intronic |  |  |
| *LOC_Os04g47870* | *Os04g0566500* | 4_28439639 | C | T | intronic |  |  |
| *LOC_Os04g47870* | *Os04g0566500* | 4_28439727 | T | C | intronic |  |  |
| *LOC_Os04g47870* | *Os04g0566500* | 4_28439736 | G | C | intronic |  |  |
| *LOC_Os04g47870* | *Os04g0566500* | 4_28439803 | G | T | intronic |  |  |
| *LOC_Os04g47870* | *Os04g0566500* | 4_28439809 | C | T | intronic |  |  |
| *LOC_Os04g47870* | *Os04g0566500* | 4_28440106 | C | T | intronic |  |  |
| *LOC_Os04g47870* | *Os04g0566500* | 4_28440162 | A | C | intronic |  |  |
| *LOC_Os04g47870* | *Os04g0566500* | 4_28440757 | T | C | intronic |  |  |
| *LOC_Os04g47870* | *Os04g0566500* | 4_28440874 | C | T | intronic |  |  |
| *LOC_Os04g47870* | *Os04g0566500* | 4_28441277 | A | C | intronic |  |  |
| *LOC_Os04g47870* | *Os04g0566500* | 4_28441656 | A | T | upstream |  |  |
| *LOC_Os04g47870* | *Os04g0566500* | 4_28441785 | T | G | upstream |  |  |
| *LOC_Os04g47870* | *Os04g0566500* | 4_28441956 | T | C | upstream |  |  |
| *LOC_Os04g47870* | *Os04g0566500* | 4_28442112 | G | A | upstream |  |  |
| *LOC_Os04g47870* | *Os04g0566500* | 4_28442152 | G | A | upstream |  |  |
| *LOC_Os04g47870* | *Os04g0566500* | 4_28442154 | A | G | upstream |  |  |
| *LOC_Os04g47870* | *Os04g0566500* | 4_28442219 | C | A | upstream |  |  |
| *LOC_Os04g47870* | *Os04g0566500* | 4_28442220 | A | C | upstream |  |  |
| *LOC_Os04g47870* | *Os04g0566500* | 4_28442308 | A | G | upstream |  |  |
| *LOC_Os04g47870* | *Os04g0566500* | 4_28442313 | A | G | upstream |  |  |
| *LOC_Os04g47870* | *Os04g0566500* | 4_28442321 | G | A | upstream |  |  |
| *LOC_Os04g47870* | *Os04g0566500* | 4_28442427 | C | T | upstream |  |  |
| *LOC_Os04g47870* | *Os04g0566500* | 4_28442488 | A | T | upstream |  |  |
| *LOC_Os04g47870* | *Os04g0566500* | 4_28442515 | T | C | upstream |  |  |
| *LOC_Os04g47870* | *Os04g0566500* | 4_28442541 | G | A | upstream |  |  |
| *LOC_Os04g47870* | *Os04g0566500* | 4_28442570 | C | A | upstream |  |  |
| *LOC_Os04g47890* | *Os04g0566600* | 4_28469452 | C | A | upstream |  | MYB family transcription factor, putative, expressed |
| *LOC_Os04g47890* | *Os04g0566600* | 4_28469458 | A | G | upstream |  |  |
| *LOC_Os04g47890* | *Os04g0566600* | 4_28469512 | T | C | upstream |  |  |
| *LOC_Os04g47890* | *Os04g0566600* | 4_28469674 | T | C | upstream |  |  |
| *LOC_Os04g47890* | *Os04g0566600* | 4_28469786 | T | C | upstream |  |  |
| *LOC_Os04g47890* | *Os04g0566600* | 4_28470049 | G | A | upstream |  |  |
| *LOC_Os04g47890* | *Os04g0566600* | 4_28470066 | C | T | upstream |  |  |
| *LOC_Os04g47890* | *Os04g0566600* | 4_28470072 | C | T | upstream |  |  |
| *LOC_Os04g47890* | *Os04g0566600* | 4_28470327 | T | A | upstream |  |  |
| *LOC_Os04g47890* | *Os04g0566600* | 4_28470490 | T | C | UTR5 |  |  |
| *LOC_Os04g47890* | *Os04g0566600* | 4_28471159 | G | T | intronic |  |  |
| *LOC_Os04g47890* | *Os04g0566600* | 4_28471463 | G | T | exonic | nonsynonymous |  |
| *LOC_Os04g47890* | *Os04g0566600* | 4_28471547 | A | C | intronic |  |  |
| *LOC_Os04g47890* | *Os04g0566600* | 4_28471549 | C | T | intronic |  |  |
| *LOC_Os04g47890* | *Os04g0566600* | 4_28473005 | G | A | intronic |  |  |
| *LOC_Os04g47890* | *Os04g0566600* | 4_28473439 | A | G | intronic |  |  |
| *LOC_Os04g47890* | *Os04g0566600* | 4_28473680 | T | C | intronic |  |  |
| *LOC_Os04g47890* | *Os04g0566600* | 4_28473777 | T | C | intronic |  |  |
| *LOC_Os04g47890* | *Os04g0566600* | 4_28473848 | A | G | exonic | nonsynonymous |  |
| *LOC_Os04g47890* | *Os04g0566600* | 4_28474937 | A | T | downstream |  |  |
| *LOC_Os04g47890* | *Os04g0566600* | 4_28475107 | G | A | downstream |  |  |
| *LOC_Os04g47890* | *Os04g0566600* | 4_28475194 | T | G | downstream |  |  |
| *LOC_Os04g47906* | *Os04g0566800* | 4_28483490 | C | A | downstream |  | gamma-tubulin complex component 6, putative, expressed |
| *LOC_Os04g47906* | *Os04g0566800* | 4_28483673 | T | G | downstream |  |  |
| *LOC_Os04g47906* | *Os04g0566800* | 4_28483791 | T | C | downstream |  |  |
| *LOC_Os04g47906* | *Os04g0566800* | 4_28483898 | C | A | downstream |  |  |
| *LOC_Os04g47906* | *Os04g0566800* | 4_28484039 | A | C | downstream |  |  |
| *LOC_Os04g47906* | *Os04g0566800* | 4_28484779 | G | A | intronic |  |  |
| *LOC_Os04g47906* | *Os04g0566800* | 4_28485140 | A | G | intronic |  |  |
| *LOC_Os04g47906* | *Os04g0566800* | 4_28486019 | A | G | intronic |  |  |
| *LOC_Os04g47906* | *Os04g0566800* | 4_28486147 | T | G | intronic |  |  |
| *LOC_Os04g47906* | *Os04g0566800* | 4_28486201 | T | G | intronic |  |  |
| *LOC_Os04g47906* | *Os04g0566800* | 4_28486208 | A | C | intronic |  |  |
| *LOC_Os04g47906* | *Os04g0566800* | 4_28486332 | G | A | intronic |  |  |
| *LOC_Os04g47906* | *Os04g0566800* | 4_28486672 | C | T | intronic |  |  |
| *LOC_Os04g47906* | *Os04g0566800* | 4_28486797 | G | A | intronic |  |  |
| *LOC_Os04g47906* | *Os04g0566800* | 4_28486984 | C | A | intronic |  |  |
| *LOC_Os04g47906* | *Os04g0566800* | 4_28487263 | A | C | intronic |  |  |
| *LOC_Os04g47906* | *Os04g0566800* | 4_28487642 | G | A | intronic |  |  |
| *LOC_Os04g47906* | *Os04g0566800* | 4_28487643 | A | T | intronic |  |  |
| *LOC_Os04g47906* | *Os04g0566800* | 4_28487936 | T | C | intronic |  |  |
| *LOC_Os04g47906* | *Os04g0566800* | 4_28488200 | T | A | intronic |  |  |
| *LOC_Os04g47906* | *Os04g0566800* | 4_28488339 | G | A | intronic |  |  |
| *LOC_Os04g47930* | *Os04g0567200* | 4_28499099 | G | A | downstream |  | aluminum-activated malate transporter, putative, expressed |
| *LOC_Os04g47930* | *Os04g0567200* | 4_28499157 | G | A | downstream |  |  |
| *LOC_Os04g47930* | *Os04g0567200* | 4_28499188 | G | A | downstream |  |  |
| *LOC_Os04g47930* | *Os04g0567200* | 4_28499238 | C | T | downstream |  |  |
| *LOC_Os04g47930* | *Os04g0567200* | 4_28499266 | T | A | downstream |  |  |
| *LOC_Os04g47930* | *Os04g0567200* | 4_28499272 | G | T | downstream |  |  |
| *LOC_Os04g47930* | *Os04g0567200* | 4_28500046 | A | G | UTR3 |  |  |
| *LOC_Os04g47930* | *Os04g0567200* | 4_28500128 | C | A | UTR3 |  |  |
| *LOC_Os04g47930* | *Os04g0567200* | 4_28502295 | T | C | UTR5 |  |  |
| *LOC_Os04g47930* | *Os04g0567200* | 4_28502559 | C | T | upstream |  |  |
| *LOC_Os04g47930* | *Os04g0567200* | 4_28503190 | A | G | upstream |  |  |
| *LOC_Os04g47930* | *Os04g0567200* | 4_28503371 | C | T | upstream |  |  |
| *LOC_Os04g47930* | *Os04g0567200* | 4_28503465 | A | G | upstream |  |  |
| *LOC_Os04g47970* | *Os04g0567700* | 4_28520957 | G | T | upstream |  | ORM1, putative, expressed |
| *LOC_Os04g47970* | *Os04g0567700* | 4_28521811 | A | G | upstream |  |  |
| *LOC_Os04g47970* | *Os04g0567700* | 4_28522104 | T | G | UTR5 |  |  |
| *LOC_Os04g47970* | *Os04g0567700* | 4_28522617 | T | G | intronic |  |  |
| *LOC_Os04g47970* | *Os04g0567700* | 4_28522725 | A | C | intronic |  |  |
| *LOC_Os04g47970* | *Os04g0567700* | 4_28522811 | C | G | intronic |  |  |
| *LOC_Os04g47970* | *Os04g0567700* | 4_28523238 | A | G | intronic |  |  |
| *LOC_Os04g47970* | *Os04g0567700* | 4_28523277 | T | G | intronic |  |  |
| *LOC_Os04g47970* | *Os04g0567700* | 4_28523293 | T | A | intronic |  |  |
| *LOC_Os04g47970* | *Os04g0567700* | 4_28524144 | T | G | intronic |  |  |
| *LOC_Os04g47970* | *Os04g0567700* | 4_28524272 | G | C | intronic |  |  |
| *LOC_Os04g47970* | *Os04g0567700* | 4_28524328 | T | C | intronic |  |  |
| *LOC_Os04g47970* | *Os04g0567700* | 4_28524637 | A | G | intronic |  |  |
| *LOC_Os04g47970* | *Os04g0567700* | 4_28524779 | A | T | intronic |  |  |
| *LOC_Os04g47970* | *Os04g0567700* | 4_28524886 | A | G | intronic |  |  |
| *LOC_Os04g47970* | *Os04g0567700* | 4_28524994 | T | G | intronic |  |  |
| *LOC_Os04g47970* | *Os04g0567700* | 4_28525192 | T | C | exonic | nonsynonymous SNV |  |
| *LOC_Os04g47970* | *Os04g0567700* | 4_28525370 | A | G | UTR3 |  |  |
| *LOC_Os04g47970* | *Os04g0567700* | 4_28525411 | G | A | UTR3 |  |  |
| *LOC_Os04g47970* | *Os04g0567700* | 4_28525986 | T | G | downstream |  |  |
| *LOC_Os04g47970* | *Os04g0567700* | 4_28526103 | A | T | downstream |  |  |
| *LOC_Os04g47970* | *Os04g0567700* | 4_28526360 | A | T | downstream |  |  |
| *LOC_Os04g47970* | *Os04g0567700* | 4_28526458 | G | A | downstream |  |  |
| *LOC_Os04g47990* | *Os04g0567800* | 4_28534750 | G | C | downstream |  | dof zinc finger domain containing protein, putative, expressed |
| *LOC_Os04g47990* | *Os04g0567800* | 4_28534860 | C | T | downstream |  |  |
| *LOC_Os04g47990* | *Os04g0567800* | 4_28535026 | G | A | UTR3 |  |  |
| *LOC_Os04g47990* | *Os04g0567800* | 4_28535152 | A | C | UTR3 |  |  |
| *LOC_Os04g47990* | *Os04g0567800* | 4_28536875 | G | A | upstream |  |  |
| *LOC_Os04g47990* | *Os04g0567800* | 4_28537412 | C | T | upstream |  |  |
| *LOC_Os04g48010* | *Os04g0568300* | 4_28563008 | C | T | upstream |  | WD-40 repeat family protein, putative, expressed |
| *LOC_Os04g48010* | *Os04g0568300* | 4_28563250 | T | G | upstream |  |  |
| *LOC_Os04g48010* | *Os04g0568300* | 4_28563282 | A | G | upstream |  |  |
| *LOC_Os04g48010* | *Os04g0568300* | 4_28563550 | T | A | upstream |  |  |
| *LOC_Os04g48010* | *Os04g0568300* | 4_28563616 | A | C | upstream |  |  |
| *LOC_Os04g48010* | *Os04g0568300* | 4_28563641 | G | T | upstream |  |  |
| *LOC_Os04g48010* | *Os04g0568300* | 4_28564144 | T | C | intronic |  |  |
| *LOC_Os04g48010* | *Os04g0568300* | 4_28565776 | A | C | intronic |  |  |
| *LOC_Os04g48010* | *Os04g0568300* | 4_28565804 | T | A | intronic |  |  |
| *LOC_Os04g48010* | *Os04g0568300* | 4_28566769 | T | A | intronic |  |  |
| *LOC_Os04g48010* | *Os04g0568300* | 4_28567929 | T | C | intronic |  |  |
| *LOC_Os04g48010* | *Os04g0568300* | 4_28568855 | T | G | UTR3 |  |  |
| *LOC_Os04g48010* | *Os04g0568300* | 4_28568947 | T | C | UTR3 |  |  |
| *LOC_Os04g48010* | *Os04g0568300* | 4_28569046 | G | T | UTR3 |  |  |
| *LOC_Os04g48010* | *Os04g0568300* | 4_28569528 | C | T | downstream |  |  |
| *LOC_Os04g48010* | *Os04g0568300* | 4_28569698 | C | T | downstream |  |  |
| *LOC_Os04g48010* | *Os04g0568300* | 4_28569885 | C | A | downstream |  |  |
| *LOC_Os04g48010* | *Os04g0568300* | 4_28570092 | C | T | downstream |  |  |
| *LOC_Os04g48010* | *Os04g0568300* | 4_28570132 | C | G | downstream |  |  |
| *LOC_Os04g48020* | *Os04g0568600* | 4_28573677 | T | C | exonic | synonymous | 3-hexulose-6-phosphate isomerase, putative, expressed |
| *LOC_Os04g48030* | *Os04g0568700* | 4_28574775 | T | C | intronic |  | heat stress transcription factor B-1, putative, expressed |
| *LOC_Os04g48030* | *Os04g0568700* | 4_28575530 | G | T | UTR3 |  |  |
| *LOC_Os04g48030* | *Os04g0568700* | 4_28575590 | A | C | UTR3 |  |  |
| *LOC_Os04g48030* | *Os04g0568700* | 4_28575678 | C | A | UTR3 |  |  |
| *LOC_Os04g48050* | *Os04g0568900* | 4_28592262 | G | A | UTR3 |  | RING zinc finger protein, putative, expressed |
| *LOC_Os04g48050* | *Os04g0568900* | 4_28592671 | A | T | intronic |  |  |
| *LOC_Os04g48050* | *Os04g0568900* | 4_28592798 | T | C | intronic |  |  |
| *LOC_Os04g48050* | *Os04g0568900* | 4_28592957 | C | G | intronic |  |  |
| *LOC_Os04g48050* | *Os04g0568900* | 4_28593297 | G | A | intronic |  |  |
| *LOC_Os04g48050* | *Os04g0568900* | 4_28593323 | T | C | intronic |  |  |
| *LOC_Os04g48050* | *Os04g0568900* | 4_28593340 | C | A | intronic |  |  |
| *LOC_Os04g48050* | *Os04g0568900* | 4_28594829 | C | T | intronic |  |  |
| *LOC_Os04g48050* | *Os04g0568900* | 4_28595318 | A | G | exonic |  |  |
| *LOC_Os04g48060* | *Os04g0569000* | 4_28596713 | G | A | upstream |  | RFC2 - Putative clamp loader of PCNA, replication factor C subunit 2, expressed |
| *LOC_Os04g48060* | *Os04g0569000* | 4_28596754 | T | G | upstream |  |  |
| *LOC_Os04g48060* | *Os04g0569000* | 4_28596815 | T | C | upstream |  |  |
| *LOC_Os04g48060* | *Os04g0569000* | 4_28596883 | G | A | upstream |  |  |
| *LOC_Os04g48060* | *Os04g0569000* | 4_28596972 | T | A | upstream |  |  |
| *LOC_Os04g48060* | *Os04g0569000* | 4_28597607 | A | C | intronic |  |  |
| *LOC_Os04g48060* | *Os04g0569000* | 4_28598129 | C | G | intronic |  |  |
| *LOC_Os04g48060* | *Os04g0569000* | 4_28598200 | T | C | intronic |  |  |
| *LOC_Os04g48060* | *Os04g0569000* | 4_28598792 | C | T | intronic |  |  |
| *LOC_Os04g48060* | *Os04g0569000* | 4_28598825 | T | C | intronic |  |  |
| *LOC_Os04g48060* | *Os04g0569000* | 4_28599038 | A | G | intronic |  |  |
| *LOC_Os04g48060* | *Os04g0569000* | 4_28599249 | C | G | intronic |  |  |
| *LOC_Os04g48060* | *Os04g0569000* | 4_28599613 | G | T | intronic |  |  |
| *LOC_Os04g48060* | *Os04g0569000* | 4_28600294 | A | G | UTR3 |  |  |
| *LOC_Os04g48060* | *Os04g0569000* | 4_28600536 | C | A | downstream |  |  |
| *LOC_Os04g48060* | *Os04g0569000* | 4_28600601 | A | T | downstream |  |  |
| *LOC_Os04g48060* | *Os04g0569000* | 4_28600603 | A | C | downstream |  |  |
| *LOC_Os04g48060* | *Os04g0569000* | 4_28600536 | C | A | downstream |  |  |
| *LOC_Os04g48060* | *Os04g0569000* | 4_28600601 | A | T | downstream |  |  |
| *LOC_Os04g48060* | *Os04g0569000* | 4_28600603 | A | C | downstream |  |  |
| *LOC_Os04g48070* | *Os04g0569100* | 4_28601130 | G | A | UTR3 |  | homeobox and START domains containing protein, putative, expressed |
| *LOC_Os04g48070* | *Os04g0569100* | 4_28601155 | T | C | UTR3 |  |  |
| *LOC_Os04g48070* | *Os04g0569100* | 4_28601621 | C | T | intronic |  |  |
| *LOC_Os04g48070* | *Os04g0569100* | 4_28601660 | G | A | intronic |  |  |
| *LOC_Os04g48070* | *Os04g0569100* | 4_28602055 | T | C | intronic |  |  |
| *LOC_Os04g48070* | *Os04g0569100* | 4_28602234 | C | G | intronic |  |  |
| *LOC_Os04g48070* | *Os04g0569100* | 4_28603050 | G | A | exonic | synonymous |  |
| *LOC_Os04g48070* | *Os04g0569100* | 4_28603059 | C | T | exonic | synonymous |  |
| *LOC_Os04g48070* | *Os04g0569100* | 4_28603182 | C | A | intronic |  |  |
| *LOC_Os04g48070* | *Os04g0569100* | 4_28603391 | C | T | intronic |  |  |
| *LOC_Os04g48070* | *Os04g0569100* | 4_28603502 | T | A | intronic |  |  |
| *LOC_Os04g48070* | *Os04g0569100* | 4_28603961 | A | G | intronic |  |  |
| *LOC_Os04g48070* | *Os04g0569100* | 4_28604374 | T | A | intronic |  |  |
| *LOC_Os04g48070* | *Os04g0569100* | 4_28604645 | T | G | intronic |  |  |
| *LOC_Os04g48070* | *Os04g0569100* | 4_28604876 | C | T | exonic |  |  |
| *LOC_Os04g48070* | *Os04g0569100* | 4_28605877 | G | C | exonic |  |  |
| *LOC_Os04g48070* | *Os04g0569100* | 4_28606952 | A | G | UTR5 |  |  |
| *LOC_Os04g48070* | *Os04g0569100* | 4_28607808 | C | A | upstream |  |  |
| *LOC_Os04g48070* | *Os04g0569100* | 4_28607895 | C | T | upstream |  |  |
| *LOC_Os04g48070* | *Os04g0569100* | 4_28607971 | A | G | upstream |  |  |
| *LOC_Os04g48070* | *Os04g0569100* | 4_28607976 | G | A | upstream |  |  |
| *LOC_Os04g48130* | *Os04g0569300* | 4_28642356 | A | G | upstream |  | OsRhmbd11 - Putative Rhomboid homologue, expressed |
| *LOC_Os04g48130* | *Os04g0569300* | 4_28642391 | T | C | upstream |  |  |
| *LOC_Os04g48130* | *Os04g0569300* | 4_28642487 | G | C | upstream |  |  |
| *LOC_Os04g48130* | *Os04g0569300* | 4_28642562 | C | G | upstream |  |  |
| *LOC_Os04g48130* | *Os04g0569300* | 4_28644026 | A | C | exonic |  |  |
| *LOC_Os04g48140* | *Os04g0569400* | 4_28645949 | C | T | exonic |  | methyltransferase, putative, expressed |
| *LOC_Os04g48140* | *Os04g0569400* | 4_28646652 | T | G | intronic |  |  |
| *LOC_Os04g48140* | *Os04g0569400* | 4_28646695 | A | T | intronic |  |  |
| *LOC_Os04g48140* | *Os04g0569400* | 4_28646709 | C | T | intronic |  |  |
| *LOC_Os04g48140* | *Os04g0569400* | 4_28646833 | T | C | intronic |  |  |
| *LOC_Os04g48140* | *Os04g0569400* | 4_28646900 | C | T | intronic |  |  |
| *LOC_Os04g48140* | *Os04g0569400* | 4_28647007 | T | C | intronic |  |  |
| *LOC_Os04g48140* | *Os04g0569400* | 4_28648912 | G | T | intronic |  |  |
| *LOC_Os04g48160* | *Os04g0569900* | 4_28663153 | T | C | downstream |  | IQ calmodulin-binding motif family protein, putative, expressed |
| *LOC_Os04g48160* | *Os04g0569900* | 4_28663408 | T | C | downstream |  |  |
| *LOC_Os04g48160* | *Os04g0569900* | 4_28663626 | C | A | downstream |  |  |
| *LOC_Os04g48160* | *Os04g0569900* | 4_28663829 | A | G | downstream |  |  |
| *LOC_Os04g48160* | *Os04g0569900* | 4_28663831 | G | A | downstream |  |  |
| *LOC_Os04g48160* | *Os04g0569900* | 4_28665020 | C | T | exonic |  |  |
| *LOC_Os04g48160* | *Os04g0569900* | 4_28666413 | C | T | UTR5 |  |  |
| *LOC_Os04g48160* | *Os04g0569900* | 4_28666445 | A | G | upstream |  |  |
| *LOC_Os04g48160* | *Os04g0569900* | 4_28666825 | G | T | upstream |  |  |
| *LOC_Os04g48160* | *Os04g0569900* | 4_28666965 | C | T | upstream |  |  |
| *LOC_Os04g48160* | *Os04g0569900* | 4_28667021 | T | G | upstream |  |  |
| *LOC_Os04g48160* | *Os04g0569900* | 4_28667081 | G | T | upstream |  |  |
| *LOC_Os04g48160* | *Os04g0569900* | 4_28667233 | G | A | upstream |  |  |
| *LOC_Os04g48160* | *Os04g0569900* | 4_28667254 | G | A | upstream |  |  |
| *LOC_Os04g48160* | *Os04g0569900* | 4_28667288 | G | A | upstream |  |  |
| *LOC_Os04g48160* | *Os04g0569900* | 4_28667320 | A | G | upstream |  |  |
| *LOC_Os04g48160* | *Os04g0569900* | 4_28667339 | A | T | upstream |  |  |
| *LOC_Os04g48170* | *Os04g0570000* | 4_28672833 | T | C | UTR3 |  | cytochrome P450, putative, expressed |
| *LOC_Os04g48170* | *Os04g0570000* | 4_28672960 | T | C | UTR3 |  |  |
| *LOC_Os04g48170* | *Os04g0570000* | 4_28673373 | G | A | intronic |  |  |
| *LOC_Os04g48170* | *Os04g0570000* | 4_28673476 | A | C | exonic |  |  |
| *LOC_Os04g48170* | *Os04g0570000* | 4_28673732 | A | T | intronic |  |  |
| *LOC_Os04g48170* | *Os04g0570000* | 4_28674257 | A | T | intronic |  |  |
| *LOC_Os04g48170* | *Os04g0570000* | 4_28674361 | A | C | intronic |  |  |
| *LOC_Os04g48170* | *Os04g0570000* | 4_28674482 | G | A | intronic |  |  |
| *LOC_Os04g48170* | *Os04g0570000* | 4_28674610 | A | G | exonic |  |  |
| *LOC_Os04g48170* | *Os04g0570000* | 4_28674768 | A | G | intronic |  |  |
| *LOC_Os04g48170* | *Os04g0570000* | 4_28675483 | T | C | intronic |  |  |
| *LOC_Os04g48170* | *Os04g0570000* | 4_28675882 | G | A | intronic |  |  |
| *LOC_Os04g48170* | *Os04g0570000* | 4_28676027 | A | G | intronic |  |  |
| *LOC_Os04g48170* | *Os04g0570000* | 4_28676159 | A | G | exonic |  |  |
| *LOC_Os04g48170* | *Os04g0570000* | 4_28676688 | T | C | exonic |  |  |
| *LOC_Os04g48170* | *Os04g0570000* | 4_28676834 | G | A | exonic |  |  |
| *LOC_Os04g48170* | *Os04g0570000* | 4_28677101 | C | T | exonic |  |  |
| *LOC_Os04g48200* | *Os04g0570500* | 4_28704314 | A | G | exonic |  | cytochrome P450, putative, expressed |
| *LOC_Os04g48200* | *Os04g0570500* | 4_28704863 | T | A | intronic |  |  |
| *LOC_Os04g48200* | *Os04g0570500* | 4_28705144 | A | T | intronic |  |  |
| *LOC_Os04g48200* | *Os04g0570500* | 4_28705281 | A | G | intronic |  |  |
| *LOC_Os04g48200* | *Os04g0570500* | 4_28705811 | G | C | intronic |  |  |
| *LOC_Os04g48200* | *Os04g0570500* | 4_28705906 | A | G | intronic |  |  |
| *LOC_Os04g48200* | *Os04g0570500* | 4_28706193 | C | T | intronic |  |  |
| *LOC_Os04g48200* | *Os04g0570500* | 4_28706840 | C | T | intronic |  |  |
| *LOC_Os04g48200* | *Os04g0570500* | 4_28706913 | G | T | intronic |  |  |
| *LOC_Os04g48200* | *Os04g0570500* | 4_28706961 | C | T | intronic |  |  |
| *LOC_Os04g48200* | *Os04g0570500* | 4_28707021 | T | C | intronic |  |  |
| *LOC_Os04g48200* | *Os04g0570500* | 4_28707406 | C | T | intronic |  |  |
| *LOC_Os04g48200* | *Os04g0570500* | 4_28707444 | G | A | exonic |  |  |
| *LOC_Os04g48200* | *Os04g0570500* | 4_28707573 | G | A | exonic |  |  |
| *LOC_Os04g48200* | *Os04g0570500* | 4_28708052 | C | T | exonic |  |  |
| *LOC_Os04g48200* | *Os04g0570500* | 4_28708444 | A | G | upstream |  |  |
| *LOC_Os04g48210* | *Os04g0570600* | 4_28714540 | A | G | downstream |  | cytochrome P450, putative, expressed |
| *LOC_Os04g48210* | *Os04g0570600* | 4_28714826 | G | T | downstream |  |  |
| *LOC_Os04g48210* | *Os04g0570600* | 4_28715597 | T | C | UTR3 |  |  |
| *LOC_Os04g48210* | *Os04g0570600* | 4_28715615 | A | G | UTR3 |  |  |
| *LOC_Os04g48210* | *Os04g0570600* | 4_28715652 | T | C | UTR3 |  |  |
| *LOC_Os04g48210* | *Os04g0570600* | 4_28716311 | A | C | intronic |  |  |
| *LOC_Os04g48210* | *Os04g0570600* | 4_28717130 | C | T | intronic |  |  |
| *LOC_Os04g48210* | *Os04g0570600* | 4_28717199 | T | A | intronic |  |  |
| *LOC_Os04g48210* | *Os04g0570600* | 4_28717269 | T | C | intronic |  |  |
| *LOC_Os04g48210* | *Os04g0570600* | 4_28717323 | T | C | intronic |  |  |
| *LOC_Os04g48210* | *Os04g0570600* | 4_28717659 | C | T | intronic |  |  |
| *LOC_Os04g48210* | *Os04g0570600* | 4_28717765 | C | T | intronic |  |  |
| *LOC_Os04g48210* | *Os04g0570600* | 4_28717812 | C | T | intronic |  |  |
| *LOC_Os04g48210* | *Os04g0570600* | 4_28718775 | T | C | intronic |  |  |
| *LOC_Os04g48210* | *Os04g0570600* | 4_28718854 | T | G | intronic |  |  |
| *LOC_Os04g48210* | *Os04g0570600* | 4_28718892 | C | A | intronic |  |  |
| *LOC_Os04g48210* | *Os04g0570600* | 4_28720452 | C | T | intronic |  |  |
| *LOC_Os04g48230* | *Os04g0570800* | 4_28722899 | A | C | UTR3 |  | dehydration response related protein, putative, expressed |
| *LOC_Os04g48230* | *Os04g0570800* | 4_28723198 | A | T | intronic |  |  |
| *LOC_Os04g48230* | *Os04g0570800* | 4_28723199 | A | T | intronic |  |  |
| *LOC_Os04g48230* | *Os04g0570800* | 4_28723972 | C | T | UTR3 |  |  |
| *LOC_Os04g48230* | *Os04g0570800* | 4_28724000 | A | C | UTR3 |  |  |
| *LOC_Os04g48230* | *Os04g0570800* | 4_28724314 | G | A | exonic |  |  |
| *LOC_Os04g48230* | *Os04g0570800* | 4_28725260 | G | A | intronic |  |  |
| *LOC_Os04g48230* | *Os04g0570800* | 4_28725578 | A | C | exonic |  |  |
| *LOC_Os04g48230* | *Os04g0570800* | 4_28726187 | A | C | intronic |  |  |
| *LOC_Os04g48230* | *Os04g0570800* | 4_28728173 | T | A | upstream |  |  |
| *LOC_Os04g48230* | *Os04g0570800* | 4_28728192 | C | T | upstream |  |  |
| *LOC_Os04g48230* | *Os04g0570800* | 4_28728322 | G | T | upstream |  |  |
| *LOC_Os04g48230* | *Os04g0570800* | 4_28728489 | C | G | upstream |  |  |
| *LOC_Os04g48260* | *Os04g0571200* | 4_28743504 | T | G | upstream |  | zinc RING finger protein, putative, expressed |
| *LOC_Os04g48260* | *Os04g0571200* | 4_28743525 | T | G | upstream |  |  |
| *LOC_Os04g48260* | *Os04g0571200* | 4_28743550 | T | C | upstream |  |  |
| *LOC_Os04g48260* | *Os04g0571200* | 4_28743735 | C | T | upstream |  |  |
| *LOC_Os04g48260* | *Os04g0571200* | 4_28743770 | T | C | upstream |  |  |
| *LOC_Os04g48260* | *Os04g0571200* | 4_28744669 | T | G | intronic |  |  |
| *LOC_Os04g48260* | *Os04g0571200* | 4_28744735 | A | T | intronic |  |  |
| *LOC_Os04g48260* | *Os04g0571200* | 4_28744768 | A | G | intronic |  |  |
| *LOC_Os04g48260* | *Os04g0571200* | 4_28745041 | C | A | intronic |  |  |
| *LOC_Os04g48260* | *Os04g0571200* | 4_28745343 | C | T | intronic |  |  |
| *LOC_Os04g48260* | *Os04g0571200* | 4_28745580 | A | G | exonic |  |  |
| *LOC_Os04g48260* | *Os04g0571200* | 4_28745638 | C | T | exonic |  |  |
| *LOC_Os04g48260* | *Os04g0571200* | 4_28746483 | A | G | intronic |  |  |
| *LOC_Os04g48260* | *Os04g0571200* | 4_28747141 | T | C | intronic |  |  |
| *LOC_Os04g48260* | *Os04g0571200* | 4_28747195 | A | G | intronic |  |  |
| *LOC_Os04g48260* | *Os04g0571200* | 4_28747360 | C | T | intronic |  |  |
| *LOC_Os04g48260* | *Os04g0571200* | 4_28747824 | T | C | UTR3 |  |  |
| *LOC_Os04g48260* | *Os04g0571200* | 4_28747833 | T | A | UTR3 |  |  |
| *LOC_Os04g48260* | *Os04g0571200* | 4_28747836 | A | C | UTR3 |  |  |
| *LOC_Os04g48260* | *Os04g0571200* | 4_28747838 | T | G | UTR3 |  |  |
| *LOC_Os04g48260* | *Os04g0571200* | 4_28747840 | T | C | UTR3 |  |  |
| *LOC_Os04g48260* | *Os04g0571200* | 4_28747972 | A | G | downstream |  |  |
| *LOC_Os04g48260* | *Os04g0571200* | 4_28748091 | T | C | downstream |  |  |
| *LOC_Os04g48260* | *Os04g0571200* | 4_28748123 | T | C | downstream |  |  |
| *LOC_Os04g48260* | *Os04g0571200* | 4_28748774 | G | T | downstream |  |  |
| *LOC_Os04g48270* | *Os04g0571300* | 4_28750463 | T | G | upstream |  | OsFBX148 - F-box domain containing protein, expressed |
| *LOC_Os04g48270* | *Os04g0571300* | 4_28751101 | G | C | upstream |  |  |
| *LOC_Os04g48270* | *Os04g0571300* | 4_28751251 | A | G | UTR5 |  |  |
| *LOC_Os04g48270* | *Os04g0571300* | 4_28751895 | C | T | exonic |  |  |
| *LOC_Os04g48270* | *Os04g0571300* | 4_28752110 | A | T | intronic |  |  |
| *LOC_Os04g48270* | *Os04g0571300* | 4_28752182 | A | G | intronic |  |  |
| *LOC_Os04g48270* | *Os04g0571300* | 4_28752748 | G | A | UTR3 |  |  |
| *LOC_Os04g48270* | *Os04g0571300* | 4_28752911 | C | T | UTR3 |  |  |
| *LOC_Os04g48270* | *Os04g0571300* | 4_28753223 | G | A | intronic |  |  |
| *LOC_Os04g48270* | *Os04g0571300* | 4_28753749 | A | G | exonic |  |  |
| *LOC_Os04g48270* | *Os04g0571300* | 4_28754119 | T | A | UTR3 |  |  |
| *LOC_Os04g48270* | *Os04g0571300* | 4_28754593 | C | T | downstream |  |  |
| *LOC_Os04g48270* | *Os04g0571300* | 4_28754594 | T | C | downstream |  |  |
| *LOC_Os04g48270* | *Os04g0571300* | 4_28754615 | A | G | downstream |  |  |
| *LOC_Os04g48270* | *Os04g0571300* | 4_28754706 | C | T | downstream |  |  |
| *LOC_Os04g48270* | *Os04g0571300* | 4_28754819 | G | A | downstream |  |  |
| *LOC_Os04g48270* | *Os04g0571300* | 4_28754870 | C | T | downstream |  |  |
| *LOC_Os04g48270* | *Os04g0571300* | 4_28754944 | G | A | downstream |  |  |
| *LOC_Os04g48270* | *Os04g0571300* | 4_28755036 | C | T | downstream |  |  |
| *LOC_Os04g48270* | *Os04g0571300* | 4_28755135 | T | C | downstream |  |  |
| *LOC_Os04g48270* | *Os04g0571300* | 4_28755284 | T | C | downstream |  |  |
| *LOC_Os04g48290* | *Os04g0571600* | 4_28774614 | A | C | UTR3 |  | MATE efflux family protein, putative, expressed |
| *LOC_Os04g48310* | *Os04g0571800* | 4_28787784 | G | A | upstream |  | RING-H2 finger protein, putative, expressed |
| *LOC_Os04g48310* | *Os04g0571800* | 4_28787816 | A | G | upstream |  |  |
| *LOC_Os04g48310* | *Os04g0571800* | 4_28789837 | G | A | intronic |  |  |
| *LOC_Os04g48310* | *Os04g0571800* | 4_28789989 | T | G | exonic |  |  |
| *LOC_Os04g48310* | *Os04g0571800* | 4_28790065 | A | T | intronic |  |  |
| *LOC_Os04g48310* | *Os04g0571800* | 4_28790779 | C | A | exonic |  |  |
| *LOC_Os04g48310* | *Os04g0571800* | 4_28791095 | T | C | UTR3 |  |  |
| *LOC_Os04g48330* | *Os04g0572200* | 4_28814574 | T | G | UTR3 |  | ethylene-responsive transcription factor ERF027, putative, expressed |
| *LOC_Os04g48330* | *Os04g0572200* | 4_28815062 | A | G | downstream |  |  |
| *LOC_Os04g48330* | *Os04g0572200* | 4_28815141 | A | G | downstream |  |  |
| *LOC_Os04g48330* | *Os04g0572200* | 4_28815156 | C | T | downstream |  |  |
| *LOC_Os04g48330* | *Os04g0572200* | 4_28815651 | C | T | downstream |  |  |
| *LOC_Os04g48330* | *Os04g0572200* | 4_28815652 | A | G | downstream |  |  |
| *LOC_Os04g48350* | *Os04g0572400* | 4_28819924 | G | T | upstream |  | dehydration-responsive element-binding protein, putative, expressed |
| *LOC_Os04g48350* | *Os04g0572400* | 4_28820101 | T | G | upstream |  |  |
| *LOC_Os04g48350* | *Os04g0572400* | 4_28820387 | T | G | upstream |  |  |
| *LOC_Os04g48350* | *Os04g0572400* | 4_28820388 | T | C | upstream |  |  |
| *LOC_Os04g48350* | *Os04g0572400* | 4_28821501 | T | G | exonic |  |  |
| *LOC_Os04g48350* | *Os04g0572400* | 4_28821696 | G | A | downstream |  |  |
| *LOC_Os04g48350* | *Os04g0572400* | 4_28821954 | T | C | downstream |  |  |
| *LOC_Os04g48350* | *Os04g0572400* | 4_28822311 | C | A | downstream |  |  |
| *LOC_Os04g48350* | *Os04g0572400* | 4_28822392 | C | T | downstream |  |  |
| *LOC_Os04g48350* | *Os04g0572400* | 4_28822408 | A | G | downstream |  |  |
| *LOC_Os04g48370* | *Os04g0572600* | 4_28840299 | G | A | upstream |  | RNA polymerase IV subunit, putative, expressed |
| *LOC_Os04g48370* | *Os04g0572600* | 4_28840694 | T | C | upstream |  |  |
| *LOC_Os04g48370* | *Os04g0572600* | 4_28841078 | A | G | upstream |  |  |
| *LOC_Os04g48370* | *Os04g0572600* | 4_28841557 | T | C | intronic |  |  |
| *LOC_Os04g48370* | *Os04g0572600* | 4_28841594 | G | A | intronic |  |  |
| *LOC_Os04g48370* | *Os04g0572600* | 4_28841872 | C | T | intronic |  |  |
| *LOC_Os04g48370* | *Os04g0572600* | 4_28841999 | T | G | intronic |  |  |
| *LOC_Os04g48370* | *Os04g0572600* | 4_28842181 | A | C | intronic |  |  |
| *LOC_Os04g48370* | *Os04g0572600* | 4_28842546 | G | A | intronic |  |  |
| *LOC_Os04g48370* | *Os04g0572600* | 4_28842950 | A | G | intronic |  |  |
| *LOC_Os04g48370* | *Os04g0572600* | 4_28842969 | A | T | intronic |  |  |
| *LOC_Os04g48370* | *Os04g0572600* | 4_28843231 | A | G | intronic |  |  |
| *LOC_Os04g48370* | *Os04g0572600* | 4_28843322 | C | T | intronic |  |  |
| *LOC_Os04g48370* | *Os04g0572600* | 4_28843380 | T | G | intronic |  |  |
| *LOC_Os04g48370* | *Os04g0572600* | 4_28843443 | A | T | exonic |  |  |
| *LOC_Os04g48370* | *Os04g0572600* | 4_28844852 | G | A | exonic |  |  |
| *LOC_Os04g48370* | *Os04g0572600* | 4_28845293 | C | T | intronic |  |  |
| *LOC_Os04g48370* | *Os04g0572600* | 4_28845556 | G | T | exonic |  |  |
| *LOC_Os04g48370* | *Os04g0572600* | 4_28845775 | G | A | exonic |  |  |
| *LOC_Os04g48370* | *Os04g0572600* | 4_28846899 | C | G | downstream |  |  |
| *LOC_Os04g48370* | *Os04g0572600* | 4_28847268 | C | T | downstream |  |  |
| *LOC_Os04g48370* | *Os04g0572600* | 4_28847389 | A | G | downstream |  |  |
| *LOC_Os04g48370* | *Os04g0572600* | 4_28847633 | C | T | downstream |  |  |
| *LOC_Os04g48375* | *Os04g0572700* | 4_28848308 | G | A | upstream |  | WD domain, G-beta repeat domain containing protein, expressed |
| *LOC_Os04g48375* | *Os04g0572700* | 4_28848320 | G | A | upstream |  |  |
| *LOC_Os04g48375* | *Os04g0572700* | 4_28848391 | T | C | upstream |  |  |
| *LOC_Os04g48375* | *Os04g0572700* | 4_28848404 | C | T | upstream |  |  |
| *LOC_Os04g48375* | *Os04g0572700* | 4_28848581 | A | G | upstream |  |  |
| *LOC_Os04g48375* | *Os04g0572700* | 4_28848596 | G | T | upstream |  |  |
| *LOC_Os04g48375* | *Os04g0572700* | 4_28848851 | C | A | upstream |  |  |
| *LOC_Os04g48375* | *Os04g0572700* | 4_28849878 | G | A | intronic |  |  |
| *LOC_Os04g48375* | *Os04g0572700* | 4_28850688 | A | G | UTR3 |  |  |
| *LOC_Os04g48375* | *Os04g0572700* | 4_28851029 | T | A | intronic |  |  |
| *LOC_Os04g48375* | *Os04g0572700* | 4_28851894 | T | G | UTR3 |  |  |
| *LOC_Os04g48380* | *Os04g0572800* | 4_28855272 | A | T | downstream |  | lysM domain containing protein, putative, expressed |
| *LOC_Os04g48380* | *Os04g0572800* | 4_28855463 | G | T | downstream |  |  |
| *LOC_Os04g48390* | *Os04g0573000* | 4_28862082 | A | G | upstream |  | uncharacterized membrane protein, putative, expressed |
| *LOC_Os04g48390* | *Os04g0573000* | 4_28862116 | T | G | upstream |  |  |
| *LOC_Os04g48390* | *Os04g0573000* | 4_28862420 | A | G | upstream |  |  |
| *LOC_Os04g48390* | *Os04g0573000* | 4_28862658 | G | C | UTR5 |  |  |
| *LOC_Os04g48390* | *Os04g0573000* | 4_28862786 | G | A | UTR5 |  |  |
| *LOC_Os04g48390* | *Os04g0573000* | 4_28863487 | C | T | intronic |  |  |
| *LOC_Os04g48390* | *Os04g0573000* | 4_28863770 | A | T | intronic |  |  |
| *LOC_Os04g48390* | *Os04g0573000* | 4_28864432 | G | A | intronic |  |  |
| *LOC_Os04g48390* | *Os04g0573000* | 4_28864616 | C | T | intronic |  |  |
| *LOC_Os04g48390* | *Os04g0573000* | 4_28864841 | C | G | intronic |  |  |
| *LOC_Os04g48390* | *Os04g0573000* | 4_28864911 | G | A | intronic |  |  |
| *LOC_Os04g48390* | *Os04g0573000* | 4_28865805 | A | C | exonic |  |  |
| *LOC_Os04g48390* | *Os04g0573000* | 4_28866695 | G | A | intronic |  |  |
| *LOC_Os04g48390* | *Os04g0573000* | 4_28867126 | G | T | exonic |  |  |
| *LOC_Os04g48390* | *Os04g0573000* | 4_28867647 | T | C | intronic |  |  |
| *LOC_Os04g48390* | *Os04g0573000* | 4_28867672 | A | G | intronic |  |  |
| *LOC_Os04g48390* | *Os04g0573000* | 4_28868575 | T | C | intronic |  |  |
| *LOC_Os04g48390* | *Os04g0573000* | 4_28869115 | C | G | downstream |  |  |
| *LOC_Os04g48400* | *Os04g0573100* | 4_28870510 | C | G | exonic | synonymous | HOTHEAD precursor, putative, expressed |
| *LOC_Os04g48400* | *Os04g0573100* | 4_28872058 | T | G | exonic | nonsynonymous |  |
| *LOC_Os04g48400* | *Os04g0573100* | 4_28872424 | T | G | intronic |  |  |
| *LOC_Os04g48400* | *Os04g0573100* | 4_28872568 | T | C | UTR3 |  |  |
| *LOC_Os04g48410* | *Os04g0573200* | 4_28875330 | A | G | intronic |  | copper chaperone for superoxide dismutase, putative, expressed |
| *LOC_Os04g48410* | *Os04g0573200* | 4_28876285 | A | G | exonic | nonsynonymous |  |
| *LOC_Os04g48410* | *Os04g0573200* | 4_28876708 | T | G | intronic |  |  |
| *LOC_Os04g48410* | *Os04g0573200* | 4_28876749 | A | T | intronic |  |  |
| *LOC_Os04g48410* | *Os04g0573200* | 4_28877188 | A | G | intronic |  |  |
| *LOC_Os04g48410* | *Os04g0573200* | 4_28877323 | G | A | intronic |  |  |
| *LOC_Os04g48410* | *Os04g0573200* | 4_28877340 | T | C | intronic |  |  |
| *LOC_Os04g48410* | *Os04g0573200* | 4_28877594 | C | G | intronic |  |  |
| *LOC_Os04g48410* | *Os04g0573200* | 4_28877762 | T | C | intronic |  |  |
| *LOC_Os04g48410* | *Os04g0573200* | 4_28877910 | T | G | intronic |  |  |
| *LOC_Os04g48410* | *Os04g0573200* | 4_28878030 | A | G | intronic |  |  |
| *LOC_Os04g48410* | *Os04g0573200* | 4_28878122 | A | G | intronic |  |  |
| *LOC_Os04g48410* | *Os04g0573200* | 4_28878135 | A | G | intronic |  |  |
| *LOC_Os04g48410* | *Os04g0573200* | 4_28878259 | G | A | intronic |  |  |
| *LOC_Os04g48410* | *Os04g0573200* | 4_28878746 | G | T | UTR3 |  |  |
| *LOC_Os04g48410* | *Os04g0573200* | 4_28881468 | T | C | UTR3 |  |  |
| *LOC_Os04g48410* | *Os04g0573200* | 4_28881562 | G | A | UTR3 |  |  |
| *LOC_Os04g48410* | *Os04g0573200* | 4_28881597 | T | C | UTR3 |  |  |
| *LOC_Os04g48410* | *Os04g0573200* | 4_28882358 | C | T | downstream |  |  |
| *LOC_Os04g48410* | *Os04g0573200* | 4_28882361 | C | A | downstream |  |  |
| *LOC_Os04g48410* | *Os04g0573200* | 4_28882503 | G | T | downstream |  |  |
| *LOC_Os04g48410* | *Os04g0573200* | 4_28883131 | T | G | downstream |  |  |
| *LOC_Os04g48410* | *Os04g0573200* | 4_28875330 | A | G | intronic |  |  |
| *LOC_Os04g48410* | *Os04g0573200* | 4_28876708 | T | G | intronic |  |  |
| *LOC_Os04g48410* | *Os04g0573200* | 4_28876749 | A | T | intronic |  |  |
| *LOC_Os04g48410* | *Os04g0573200* | 4_28877188 | A | G | intronic |  |  |
| *LOC_Os04g48410* | *Os04g0573200* | 4_28877323 | G | A | intronic |  |  |
| *LOC_Os04g48410* | *Os04g0573200* | 4_28877340 | T | C | intronic |  |  |
| *LOC_Os04g48410* | *Os04g0573200* | 4_28877594 | C | G | intronic |  |  |
| *LOC_Os04g48410* | *Os04g0573200* | 4_28877762 | T | C | intronic |  |  |
| *LOC_Os04g48410* | *Os04g0573200* | 4_28877910 | T | G | intronic |  |  |
| *LOC_Os04g48410* | *Os04g0573200* | 4_28878030 | A | G | intronic |  |  |
| *LOC_Os04g48410* | *Os04g0573200* | 4_28878122 | A | G | intronic |  |  |
| *LOC_Os04g48410* | *Os04g0573200* | 4_28878135 | A | G | intronic |  |  |
| *LOC_Os04g48410* | *Os04g0573200* | 4_28878259 | G | A | intronic |  |  |
| *LOC_Os04g48416* | *Os04g0573300* | 4_28881178 | C | T | exonic | synonymous | OsSub45 - Putative Subtilisin homologue, expressed |
| *LOC_Os04g48460* | *Os04g0573900* | 4_28891703 | T | A | upstream |  | cytochrome P450, putative, expressed |
| *LOC_Os04g48460* | *Os04g0573900* | 4_28892263 | C | T | upstream |  |  |
| *LOC_Os04g48460* | *Os04g0573900* | 4_28892958 | C | T | exonic | synonymous |  |
| *LOC_Os04g48460* | *Os04g0573900* | 4_28894493 | A | T | exonic | nonsynonymous |  |
| *LOC_Os04g48460* | *Os04g0573900* | 4_28894717 | T | G | UTR3 |  |  |
| *LOC_Os04g48460* | *Os04g0573900* | 4_28894753 | G | A | UTR3 |  |  |
| *LOC_Os04g48460* | *Os04g0573900* | 4_28894930 | G | A | downstream |  |  |
| *LOC_Os04g48480* | *Os04g0574100* | 4_28899910 | A | T | upstream |  | exostosin family domain containing protein, expressed |
| *LOC_Os04g48480* | *Os04g0574100* | 4_28900243 | T | C | upstream |  |  |
| *LOC_Os04g48480* | *Os04g0574100* | 4_28900482 | A | T | upstream |  |  |
| *LOC_Os04g48480* | *Os04g0574100* | 4_28900636 | C | A | upstream |  |  |
| *LOC_Os04g48480* | *Os04g0574100* | 4_28902972 | T | G | downstream |  |  |
| *LOC_Os04g48480* | *Os04g0574100* | 4_28903236 | T | C | downstream |  |  |
| *LOC_Os04g48490* | *Os04g0574200* | 4_28909336 | A | C | downstream |  | fasciclin-like arabinogalactan protein, putative, expressed |
| *LOC_Os04g48490* | *Os04g0574200* | 4_28910054 | G | A | downstream |  |  |
| *LOC_Os04g48490* | *Os04g0574200* | 4_28910102 | C | G | downstream |  |  |
| *LOC_Os04g48490* | *Os04g0574200* | 4_28910199 | A | G | downstream |  |  |
| *LOC_Os04g48490* | *Os04g0574200* | 4_28911846 | G | T | UTR5 |  |  |
| *LOC_Os04g48490* | *Os04g0574200* | 4_28911928 | G | A | upstream |  |  |
| *LOC_Os04g48490* | *Os04g0574200* | 4_28912110 | C | A | upstream |  |  |
| *LOC_Os04g48490* | *Os04g0574200* | 4_28912160 | A | G | upstream |  |  |
| *LOC_Os04g48490* | *Os04g0574200* | 4_28912410 | G | C | upstream |  |  |
| *LOC_Os04g48510* | *Os04g0574500* | 4_28922788 | C | T | intronic |  | growth regulating factor protein, putative, expressed |
| *LOC_Os04g48510* | *Os04g0574500* | 4_28923115 | T | C | exonic |  |  |
| *LOC_Os04g48520* | *Os04g0574600* | 4_28924834 | T | A | upstream | synonymous | ZOS4-12 - C2H2 zinc finger protein, expressed |
| *LOC_Os04g48520* | *Os04g0574600* | 4_28924918 | A | T | upstream |  |  |
| *LOC_Os04g48520* | *Os04g0574600* | 4_28925019 | A | G | UTR5 |  |  |
| *LOC_Os04g48520* | *Os04g0574600* | 4_28925390 | A | C | intronic |  |  |
| *LOC_Os04g48520* | *Os04g0574600* | 4_28925869 | T | C | intronic |  |  |
| *LOC_Os04g48520* | *Os04g0574600* | 4_28926033 | C | T | intronic |  |  |
| *LOC_Os04g48520* | *Os04g0574600* | 4_28926734 | C | T | exonic |  |  |
| *LOC_Os04g48520* | *Os04g0574600* | 4_28927073 | T | C | exonic | nonsynonymous |  |
| *LOC_Os04g48520* | *Os04g0574600* | 4_28927235 | A | G | intronic | synonymous |  |
| *LOC_Os04g48520* | *Os04g0574600* | 4_28927649 | T | C | exonic |  |  |
| *LOC_Os04g48520* | *Os04g0574600* | 4_28927682 | G | T | exonic | synonymous |  |
| *LOC_Os04g48520* | *Os04g0574600* | 4_28928232 | A | T | intronic |  |  |
| *LOC_Os04g48520* | *Os04g0574600* | 4_28928513 | T | A | exonic |  |  |
| *LOC_Os04g48520* | *Os04g0574600* | 4_28928655 | C | T | intronic |  |  |
| *LOC_Os04g48520* | *Os04g0574600* | 4_28928841 | T | C | exonic |  |  |
| *LOC_Os04g48540* | *Os04g0574800* | 4_28932141 | C | T | exonic |  | dihydrodipicolinate synthase, chloroplast precursor, putative, expressed |
| *LOC_Os04g48540* | *Os04g0574800* | 4_28932970 | T | C | exonic |  |  |
| *LOC_Os04g48540* | *Os04g0574800* | 4_28933219 | G | A | intronic |  |  |
| *LOC_Os04g48540* | *Os04g0574800* | 4_28933241 | A | C | intronic |  |  |
| *LOC_Os04g48540* | *Os04g0574800* | 4_28933552 | C | G | intronic |  |  |
| *LOC_Os04g48540* | *Os04g0574800* | 4_28933927 | G | A | intronic |  |  |
| *LOC_Os04g48540* | *Os04g0574800* | 4_28934077 | G | A | intronic |  |  |
| *LOC_Os04g48540* | *Os04g0574800* | 4_28934104 | C | T | intronic |  |  |
| *LOC_Os04g48540* | *Os04g0574800* | 4_28934860 | A | T | upstream |  |  |
| *LOC_Os04g48540* | *Os04g0574800* | 4_28934892 | G | A | upstream |  |  |
| *LOC_Os04g48530* | *Os04g0574800* | 4_28935139 | T | G | upstream |  | C4-dicarboxylate transporter/malic acid transport protein domain containing protein, expressed |
| *LOC_Os04g48530* | *Os04g0574700* | 4_28929801 | T | A | UTR5 |  |  |
| *LOC_Os04g48530* | *Os04g0574700* | 4_28929903 | T | C | UTR5 |  |  |
| *LOC_Os04g48530* | *Os04g0574700* | 4_28930967 | G | C | exonic |  |  |
| *LOC_Os04g48530* | *Os04g0574700* | 4_28931766 | G | A | exonic |  |  |
| *LOC_Os04g48750* | *Os04g0576800* | 4_29071138 | T | C | upstream | synonymous | 3-oxo-5-alpha-steroid 4-dehydrogenase, putative, expressed |
| *LOC_Os04g48750* | *Os04g0576800* | 4_29071427 | T | C | upstream |  |  |
| *LOC_Os04g48750* | *Os04g0576800* | 4_29071493 | T | C | upstream |  |  |
| *LOC_Os04g48750* | *Os04g0576800* | 4_29071506 | G | A | upstream |  |  |
| *LOC_Os04g48750* | *Os04g0576800* | 4_29071607 | A | G | upstream |  |  |
| *LOC_Os04g48750* | *Os04g0576800* | 4_29071937 | C | G | UTR5 |  |  |
| *LOC_Os04g48750* | *Os04g0576800* | 4_29071942 | T | C | UTR5 |  |  |
| *LOC_Os04g48750* | *Os04g0576800* | 4_29072629 | C | A | UTR5 |  |  |
| *LOC_Os04g48750* | *Os04g0576800* | 4_29076365 | G | A | downstream |  |  |
| *LOC_Os04g48750* | *Os04g0576800* | 4_29076366 | C | A | downstream |  |  |
| *LOC_Os04g48750* | *Os04g0576800* | 4_29076552 | A | G | downstream |  |  |
| *LOC_Os04g48760* | *Os04g0576900* | 4_29084013 | G | T | upstream |  | leucine-rich repeat family protein, putative, expressed |
| *LOC_Os04g48760* | *Os04g0576900* | 4_29084346 | G | A | exonic |  |  |
| *LOC_Os04g48760* | *Os04g0576900* | 4_29085458 | T | C | exonic | nonsynonymous |  |
| *LOC_Os04g48760* | *Os04g0576900* | 4_29086157 | G | T | UTR3 | synonymous |  |
| *LOC_Os04g48760* | *Os04g0576900* | 4_29086348 | T | C | UTR3 |  |  |
| *LOC_Os04g48760* | *Os04g0576900* | 4_29086589 | G | A | downstream |  |  |
| *LOC_Os04g48760* | *Os04g0576900* | 4_29086829 | G | T | downstream |  |  |
| *LOC_Os04g48760* | *Os04g0576900* | 4_29087067 | C | T | downstream |  |  |
| *LOC_Os04g48770* | *Os04g0577000* | 4_29087889 | A | G | upstream |  | ubiquitin fusion degradation protein, putative, expressed |
| *LOC_Os04g48770* | *Os04g0577000* | 4_29087952 | G | A | upstream |  |  |
| *LOC_Os04g48770* | *Os04g0577000* | 4_29087960 | A | T | upstream |  |  |
| *LOC_Os04g48770* | *Os04g0577000* | 4_29088085 | A | G | upstream |  |  |
| *LOC_Os04g48770* | *Os04g0577000* | 4_29088178 | C | G | upstream |  |  |
| *LOC_Os04g48770* | *Os04g0577000* | 4_29088394 | C | T | upstream |  |  |
| *LOC_Os04g48770* | *Os04g0577000* | 4_29088404 | C | T | upstream |  |  |
| *LOC_Os04g48770* | *Os04g0577000* | 4_29088675 | A | G | upstream |  |  |
| *LOC_Os04g48770* | *Os04g0577000* | 4_29089268 | T | G | intronic |  |  |
| *LOC_Os04g48770* | *Os04g0577000* | 4_29089305 | A | T | intronic |  |  |
| *LOC_Os04g48770* | *Os04g0577000* | 4_29089511 | G | A | intronic |  |  |
| *LOC_Os04g48770* | *Os04g0577000* | 4_29089613 | T | G | intronic |  |  |
| *LOC_Os04g48770* | *Os04g0577000* | 4_29089783 | T | C | intronic |  |  |
| *LOC_Os04g48770* | *Os04g0577000* | 4_29090201 | C | T | intronic |  |  |
| *LOC_Os04g48770* | *Os04g0577000* | 4_29090551 | A | G | intronic |  |  |
| *LOC_Os04g48770* | *Os04g0577000* | 4_29091198 | G | A | exonic |  |  |
| *LOC_Os04g48770* | *Os04g0577000* | 4_29091406 | G | A | UTR3 | synonymous |  |
| *LOC_Os04g48770* | *Os04g0577000* | 4_29091856 | C | T | intronic |  |  |
| *LOC_Os04g48770* | *Os04g0577000* | 4_29092013 | G | T | exonic |  |  |
| *LOC_Os04g48770* | *Os04g0577000* | 4_29092017 | A | G | exonic | nonsynonymous |  |
| *LOC_Os04g48770* | *Os04g0577000* | 4_29092035 | A | G | exonic | nonsynonymous |  |
| *LOC_Os04g48770* | *Os04g0577000* | 4_29092272 | G | A | intronic | nonsynonymous |  |
| *LOC_Os04g48770* | *Os04g0577000* | 4_29092294 | C | T | intronic |  |  |
| *LOC_Os04g48770* | *Os04g0577000* | 4_29092338 | A | T | intronic |  |  |
| *LOC_Os04g48770* | *Os04g0577000* | 4_29092851 | G | T | UTR3 |  |  |
| *LOC_Os04g48770* | *Os04g0577000* | 4_29092872 | C | T | UTR3 |  |  |
| *LOC_Os04g48770* | *Os04g0577000* | 4_29093171 | T | C | downstream |  |  |
| *LOC_Os04g48770* | *Os04g0577000* | 4_29093349 | G | A | downstream |  |  |
| *LOC_Os04g48770* | *Os04g0577000* | 4_29093445 | A | G | downstream |  |  |
| *LOC_Os04g48770* | *Os04g0577000* | 4_29093583 | C | A | downstream |  |  |
| *LOC_Os04g48790* | *Os04g0577200* | 4_29105411 | A | G | upstream |  | rhoGAP domain containing protein, expressed |
| *LOC_Os04g48790* | *Os04g0577200* | 4_29105418 | G | A | upstream |  |  |
| *LOC_Os04g48790* | *Os04g0577200* | 4_29105451 | G | A | upstream |  |  |
| *LOC_Os04g48790* | *Os04g0577200* | 4_29105510 | T | C | upstream |  |  |
| *LOC_Os04g48790* | *Os04g0577200* | 4_29105655 | C | T | upstream |  |  |
| *LOC_Os04g48790* | *Os04g0577200* | 4_29105671 | G | C | upstream |  |  |
| *LOC_Os04g48790* | *Os04g0577200* | 4_29105937 | G | A | upstream |  |  |
| *LOC_Os04g48790* | *Os04g0577200* | 4_29106001 | A | C | upstream |  |  |
| *LOC_Os04g48790* | *Os04g0577200* | 4_29106892 | A | G | intronic |  |  |
| *LOC_Os04g48790* | *Os04g0577200* | 4_29107915 | G | C | intronic |  |  |
| *LOC_Os04g48790* | *Os04g0577200* | 4_29108023 | G | A | exonic |  |  |
| *LOC_Os04g48790* | *Os04g0577200* | 4_29108153 | G | T | intronic | nonsynonymous |  |
| *LOC_Os04g48790* | *Os04g0577200* | 4_29108176 | G | A | intronic |  |  |
| *LOC_Os04g48790* | *Os04g0577200* | 4_29108344 | G | A | intronic |  |  |
| *LOC_Os04g48790* | *Os04g0577200* | 4_29108429 | A | C | intronic |  |  |
| *LOC_Os04g48790* | *Os04g0577200* | 4_29108606 | T | C | exonic |  |  |
| *LOC_Os04g48800* | *Os04g0577300* | 4_29111186 | C | T | intronic | nonsynonymous | GDSL-like lipase/acylhydrolase, putative, expressed |
| *LOC_Os04g48800* | *Os04g0577300* | 4_29111535 | G | C | exonic |  |  |
| *LOC_Os04g48800* | *Os04g0577300* | 4_29111830 | T | A | exonic | nonsynonymous |  |
| *LOC_Os04g48800* | *Os04g0577300* | 4_29111972 | C | T | intronic | synonymous |  |
| *LOC_Os04g48800* | *Os04g0577300* | 4_29112085 | C | T | exonic |  |  |
| *LOC_Os04g48800* | *Os04g0577300* | 4_29112457 | T | A | exonic | synonymous |  |
| *LOC_Os04g48800* | *Os04g0577300* | 4_29112477 | A | G | exonic | nonsynonymous |  |
| *LOC_Os04g48820* | *Os04g0577500* | 4_29114734 | G | A | downstream | nonsynonymous | deoxyribonuclease tatD, putative, expressed |
| *LOC_Os04g48820* | *Os04g0577500* | 4_29114739 | C | A | downstream |  |  |
| *LOC_Os04g48820* | *Os04g0577500* | 4_29114778 | G | T | downstream |  |  |
| *LOC_Os04g48820* | *Os04g0577500* | 4_29114806 | G | C | downstream |  |  |
| *LOC_Os04g48820* | *Os04g0577500* | 4_29114861 | T | C | downstream |  |  |
| *LOC_Os04g48820* | *Os04g0577500* | 4_29114874 | T | A | downstream |  |  |
| *LOC_Os04g48820* | *Os04g0577500* | 4_29115058 | C | G | downstream |  |  |
| *LOC_Os04g48820* | *Os04g0577500* | 4_29115070 | A | T | downstream |  |  |
| *LOC_Os04g48820* | *Os04g0577500* | 4_29115289 | A | T | downstream |  |  |
| *LOC_Os04g48820* | *Os04g0577500* | 4_29115874 | C | A | UTR3 |  |  |
| *LOC_Os04g48820* | *Os04g0577500* | 4_29116276 | A | G | intronic |  |  |
| *LOC_Os04g48820* | *Os04g0577500* | 4_29116865 | A | C | exonic |  |  |
| *LOC_Os04g48820* | *Os04g0577500* | 4_29116941 | T | G | exonic | nonsynonymous |  |
| *LOC_Os04g48820* | *Os04g0577500* | 4_29117009 | T | C | intronic | synonymous |  |
| *LOC_Os04g48820* | *Os04g0577500* | 4_29117388 | A | G | intronic |  |  |
| *LOC_Os04g48820* | *Os04g0577500* | 4_29117443 | T | C | intronic |  |  |
| *LOC_Os04g48820* | *Os04g0577500* | 4_29117477 | C | T | intronic |  |  |
| *LOC_Os04g48820* | *Os04g0577500* | 4_29117754 | A | G | intronic |  |  |
| *LOC_Os04g48820* | *Os04g0577500* | 4_29117901 | A | G | intronic |  |  |
| *LOC_Os04g48820* | *Os04g0577500* | 4_29118246 | A | C | intronic |  |  |
| *LOC_Os04g48820* | *Os04g0577500* | 4_29118780 | T | C | intronic |  |  |
| *LOC_Os04g48820* | *Os04g0577500* | 4_29118781 | G | A | intronic |  |  |
| *LOC_Os04g48820* | *Os04g0577500* | 4_29118965 | T | C | intronic |  |  |
| *LOC_Os04g48820* | *Os04g0577500* | 4_29119041 | T | A | intronic |  |  |
| *LOC_Os04g48820* | *Os04g0577500* | 4_29119150 | A | C | exonic |  |  |
| *LOC_Os04g48820* | *Os04g0577500* | 4_29119649 | C | T | upstream |  |  |
| *LOC_Os04g48820* | *Os04g0577500* | 4_29119709 | C | T | upstream |  |  |
| *LOC_Os04g48820* | *Os04g0577500* | 4_29119801 | G | A | upstream |  |  |
| *LOC_Os04g48820* | *Os04g0577500* | 4_29120257 | G | C | upstream |  |  |
| *LOC_Os04g48850* | *Os04g0578000* | 4_29131047 | A | G | downstream |  | aminotransferase, classes I and II, domain containing protein, expressed |
| *LOC_Os04g48850* | *Os04g0578000* | 4_29131181 | C | T | downstream |  |  |
| *LOC_Os04g48850* | *Os04g0578000* | 4_29131233 | C | T | downstream |  |  |
| *LOC_Os04g48850* | *Os04g0578000* | 4_29131379 | G | A | downstream |  |  |
| *LOC_Os04g48850* | *Os04g0578000* | 4_29131692 | T | C | downstream |  |  |
| *LOC_Os04g48850* | *Os04g0578000* | 4_29131767 | A | G | downstream |  |  |
| *LOC_Os04g48850* | *Os04g0578000* | 4_29132378 | A | G | exonic |  |  |
| *LOC_Os04g48850* | *Os04g0578000* | 4_29134854 | G | A | upstream | synonymous |  |
| *LOC_Os04g48850* | *Os04g0578000* | 4_29135602 | C | T | upstream |  |  |
| *LOC_Os04g48850* | *Os04g0578000* | 4_29135614 | C | T | upstream |  |  |
| *LOC_Os04g48870* | *Os04g0578300* | 4_29152987 | C | G | UTR5 |  | nitrilase-associated protein, putative, expressed |
| *LOC_Os04g48870* | *Os04g0578300* | 4_29153209 | G | A | exonic |  |  |
| *LOC_Os04g48870* | *Os04g0578300* | 4_29153764 | C | A | UTR3 | nonsynonymous |  |
| *LOC_Os04g48870* | *Os04g0578300* | 4_29153828 | C | T | UTR3 |  |  |
| *LOC_Os04g48870* | *Os04g0578300* | 4_29154076 | C | T | downstream |  |  |
| *LOC_Os04g48870* | *Os04g0578300* | 4_29154156 | C | T | downstream |  |  |
| *LOC_Os04g48870* | *Os04g0578300* | 4_29154271 | A | G | downstream |  |  |
| *LOC_Os04g48870* | *Os04g0578300* | 4_29154380 | G | C | downstream |  |  |
| *LOC_Os04g48870* | *Os04g0578300* | 4_29154394 | A | G | downstream |  |  |
| *LOC_Os04g48870* | *Os04g0578300* | 4_29154455 | G | A | downstream |  |  |
| *LOC_Os04g48870* | *Os04g0578300* | 4_29154465 | C | T | downstream |  |  |
| *LOC_Os04g48870* | *Os04g0578300* | 4_29154506 | C | T | downstream |  |  |
| *LOC_Os04g48870* | *Os04g0578300* | 4_29154573 | G | A | downstream |  |  |
| *LOC_Os04g48870* | *Os04g0578300* | 4_29154673 | C | T | downstream |  |  |
| *LOC_Os04g48870* | *Os04g0578300* | 4_29154675 | G | A | downstream |  |  |
| *LOC_Os04g48870* | *Os04g0578300* | 4_29154720 | G | A | downstream |  |  |
| *LOC_Os04g48870* | *Os04g0578300* | 4_29154816 | T | A | downstream |  |  |
| *LOC_Os04g48870* | *Os04g0578300* | 4_29154821 | A | G | downstream |  |  |
| *LOC_Os04g48870* | *Os04g0578300* | 4_29154927 | G | A | downstream |  |  |
| *LOC_Os04g48870* | *Os04g0578300* | 4_29154940 | A | G | downstream |  |  |
| *LOC_Os04g48880* | *Os04g0578400* | 4_29158943 | A | G | UTR3 |  | fatty acid hydroxylase, putative, expressed |
| *LOC_Os04g48880* | *Os04g0578400* | 4_29159076 | C | A | UTR3 |  |  |
| *LOC_Os04g48880* | *Os04g0578400* | 4_29159178 | G | A | UTR3 |  |  |
| *LOC_Os04g48880* | *Os04g0578400* | 4_29159404 | T | G | downstream |  |  |
| *LOC_Os04g48880* | *Os04g0578400* | 4_29159459 | C | A | downstream |  |  |
| *LOC_Os04g48880* | *Os04g0578400* | 4_29159473 | C | T | downstream |  |  |
| *LOC_Os04g48880* | *Os04g0578400* | 4_29159477 | A | G | downstream |  |  |
| *LOC_Os04g48880* | *Os04g0578400* | 4_29159897 | G | A | downstream |  |  |
| *LOC_Os04g48880* | *Os04g0578400* | 4_29159918 | C | A | downstream |  |  |
| *LOC_Os04g48880* | *Os04g0578400* | 4_29160182 | C | T | downstream |  |  |
| *LOC_Os04g48880* | *Os04g0578400* | 4_29160184 | G | A | downstream |  |  |

**Supplementary Table 7**

Table S7. Gene haplotype distribution of 162 accessions.

| No. | Accession | PL | | | | | | |  | TGP FGP GWP | | |
| --- | --- | --- | --- | --- | --- | --- | --- | --- | --- | --- | --- | --- |
|  |  | *LOC_Os01g43700* | |  | *LOC_Os09g25784* | | | |  | *LOC_Os04g47890* | | |
|  |  | HapA | HapB |  | HapA | HapB | HapC | HapD |  | HapA | HapB | HapC |
|  |  | 4.03 | -1.38 |  | -2.17 | 3.3 | 4.83 | 2.92 |  | 27 | 9 | -7 |
|  |  |  |  |  |  |  |  |  |  | 25 | 5 | -6 |
|  |  |  |  |  |  |  |  |  |  | 0.72 | 0.02 | -0.12 |
| 1 | Longjing 22 |  | ▲ |  | ▲ |  |  |  |  |  |  | ▲ |
| 2 | Longjing 28 |  | ▲ |  | ▲ |  |  |  |  |  |  | ▲ |
| 3 | Longjing 27 |  | ▲ |  | ▲ |  |  |  |  |  |  | ▲ |
| 4 | 24248 |  | ▲ |  | ▲ |  |  |  |  |  |  | ▲ |
| 5 | Tijin |  | ▲ |  | ▲ |  |  |  |  |  |  | ▲ |
| 6 | Zhongguo 91 |  | ▲ |  | ▲ |  |  |  |  |  |  | ▲ |
| 7 | Kangbingyueguang |  | ▲ |  | ▲ |  |  |  |  |  |  | ▲ |
| 8 | Sihao 4385 |  | ▲ |  | ▲ |  |  |  |  |  |  | ▲ |
| 9 | Nannongjing 1R |  | ▲ |  |  | ✔ |  |  |  |  |  | ▲ |
| 10 | Hongmangshajing |  | ▲ |  | ▲ |  |  |  |  |  |  | ▲ |
| 11 | Wanhuangdao |  | ▲ |  | ▲ |  |  |  |  |  |  | ▲ |
| 12 | Xudao 3hao |  | ▲ |  | ▲ |  |  |  |  |  |  | ▲ |
| 13 | Youzhiyueguang |  | ▲ |  |  |  | ✔ |  |  |  |  | ▲ |
| 14 | Yuedao 68 | ✔ |  |  | ▲ |  |  |  |  |  | ✔ |  |
| 15 | Longdao 8hao |  | ▲ |  | ▲ |  |  |  |  |  |  | ▲ |
| 16 | Longdao 6hao |  | ▲ |  | ▲ |  |  |  |  |  |  | ▲ |
| 17 | Qiutianxiaoding |  | ▲ |  | ▲ |  |  |  |  |  |  | ▲ |
| 18 | Zhenghan 2hao |  | ▲ |  | ▲ |  |  |  |  |  |  | ▲ |
| 19 | Xiangjing 9407 |  | ▲ |  |  |  | ✔ |  |  |  |  | ▲ |
| 20 | Nongxiang 21 | ✔ |  |  |  | ✔ |  |  |  |  | ✔ |  |
| 21 | Fengyouwan 8hao | ✔ |  |  | ▲ |  |  |  |  | ✔ |  |  |
| 22 | Xiangwanxian 17 |  | ▲ |  |  |  | ✔ |  |  |  |  | ▲ |
| 23 | Yuedao 37 | ✔ |  |  | ▲ |  |  |  |  |  | ✔ |  |
| 24 | Sujing 353 |  | ▲ |  | ▲ |  |  |  |  |  |  | ▲ |
| 25 | Zhen9424 |  | ▲ |  |  | ✔ |  |  |  |  |  | ▲ |
| 26 | Baikenuo | ✔ |  |  |  | ✔ |  |  |  | ✔ |  |  |
| 27 | Diantun502xuanzao | ✔ |  |  |  |  | ✔ |  |  |  | ✔ |  |
| 28 | Yuedao 41 | ✔ |  |  | ▲ |  |  |  |  | ✔ |  |  |
| 29 | Longnuo 3hao |  | ▲ |  | ▲ |  |  |  |  |  |  | ▲ |
| 30 | Mudanjiang 28 |  | ▲ |  | ▲ |  |  |  |  |  |  | ▲ |
| 31 | Yujing 6hao |  | ▲ |  | ▲ |  |  |  |  |  |  | ▲ |
| 32 | Shengdao808 |  | ▲ |  |  |  | ✔ |  |  |  |  | ▲ |
| 33 | Yuedao 32 | ✔ |  |  |  |  |  | ✔ |  | ✔ |  |  |
| 34 | Yuedao 107 |  | ▲ |  |  |  | ✔ |  |  |  |  | ▲ |
| 35 | Yuedao 61 | ✔ |  |  |  |  | ✔ |  |  | ✔ |  |  |
| 36 | Yuedao 50 | ✔ |  |  |  |  | ✔ |  |  |  | ✔ |  |
| 37 | Yuedao 109 | ✔ |  |  |  |  |  | ✔ |  |  | ✔ |  |
| 38 | Yuedao 62 | ✔ |  |  |  |  | ✔ |  |  | ✔ |  |  |
| 39 | Yuedao 66 | ✔ |  |  | ▲ |  |  |  |  |  | ✔ |  |
| 40 | Hongnong 5hao |  | ▲ |  | ▲ |  |  |  |  |  |  | ▲ |
| 41 | Suyunuo |  | ▲ |  | ▲ |  |  |  |  |  |  | ▲ |
| 42 | Shenlenuo |  | ▲ |  |  |  | ✔ |  |  |  |  | ▲ |
| 43 | Hongjiaozhan | ✔ |  |  | ▲ |  |  |  |  | ✔ |  |  |
| 44 | Wanjingnuo |  | ▲ |  |  |  |  | ✔ |  |  |  | ▲ |
| 45 | Nongxiang26 | ✔ |  |  |  |  | ✔ |  |  | ✔ |  |  |
| 46 | Yuedao 9 | ✔ |  |  | ▲ |  |  |  |  |  | ✔ |  |
| 47 | M1004 |  | ▲ |  |  |  |  | ✔ |  |  |  | ▲ |
| 48 | Nongxiang 25 | ✔ |  |  | ▲ |  |  |  |  | ✔ |  |  |
| 49 | Longjing 20 |  | ▲ |  |  |  | ✔ |  |  |  |  | ▲ |
| 50 | Xiangchuanwuxinbaimi |  | ▲ |  | ▲ |  |  |  |  |  |  | ▲ |
| 51 | Jindao 1007 |  | ▲ |  | ▲ |  |  |  |  |  |  | ▲ |
| 52 | Zaijinjing |  | ▲ |  |  | ✔ |  |  |  |  |  | ▲ |
| 53 | Malaihong |  | ▲ |  | ▲ |  |  |  |  |  | ✔ |  |
| 54 | Nannongjing3786 |  | ▲ |  | ▲ |  |  |  |  |  |  | ▲ |
| 55 | Wuyunjing 8hao |  | ▲ |  |  |  |  | ✔ |  |  |  | ▲ |
| 56 | Yuzhenxiang |  | ▲ |  |  |  | ✔ |  |  |  | ✔ |  |
| 57 | Yuedao24(LCV18) |  | ▲ |  |  |  |  | ✔ |  |  | ✔ |  |
| 58 | Yuedao 3 | ✔ |  |  |  |  |  | ✔ |  |  | ✔ |  |
| 59 | Yuedao 43 |  | ▲ |  |  |  | ✔ |  |  |  |  | ▲ |
| 60 | Yuedao 48 | ✔ |  |  |  |  | ✔ |  |  |  | ✔ |  |
| 61 | Yuedao 49 | ✔ |  |  |  |  | ✔ |  |  |  | ✔ |  |
| 62 | Yuedao 13 |  | ▲ |  | ▲ |  |  |  |  |  | ✔ |  |
| 63 | Wumangyedao |  | ▲ |  |  | ✔ |  |  |  |  |  | ▲ |
| 64 | Haobuqia | ✔ |  |  |  |  | ✔ |  |  |  | ✔ |  |
| 65 | Yuedao 22 | ✔ |  |  | ▲ |  |  |  |  | ✔ |  |  |
| 66 | Dongzhengwuyunjing 21 |  | ▲ |  | ▲ |  |  |  |  |  |  | ▲ |
| 67 | Yandao 8hao |  | ▲ |  | ▲ |  |  |  |  |  |  | ▲ |
| 68 | Huaidao 11hao |  | ▲ |  | ▲ |  |  |  |  |  |  | ▲ |
| 69 | Nannongjing 004 |  | ▲ |  | ▲ |  |  |  |  |  |  | ▲ |
| 70 | Zhongzuo 93 |  | ▲ |  | ▲ |  |  |  |  |  |  | ▲ |
| 71 | Xudao 5hao |  | ▲ |  | ▲ |  |  |  |  |  |  | ▲ |
| 72 | Huaidao 8hao |  | ▲ |  | ▲ |  |  |  |  |  |  | ▲ |
| 73 | Dongzheng 1640 |  | ▲ |  | ▲ |  |  |  |  |  |  | ▲ |
| 74 | Yanjing 8hao |  | ▲ |  | ▲ |  |  |  |  |  |  | ▲ |
| 75 | Huifeng 2 |  | ▲ |  | ▲ |  |  |  |  |  |  | ▲ |
| 76 | Yandao 9hao |  | ▲ |  | ▲ |  |  |  |  |  |  | ▲ |
| 77 | Lianjing 4hao |  | ▲ |  | ▲ |  |  |  |  |  |  | ▲ |
| 78 | Huifeng 1 |  | ▲ |  | ▲ |  |  |  |  |  |  | ▲ |
| 79 | Sihao 4280 |  | ▲ |  | ▲ |  |  |  |  |  |  | ▲ |
| 80 | Sihao 4330 |  | ▲ |  | ▲ |  |  |  |  |  |  | ▲ |
| 81 | Sihao 4259 |  | ▲ |  | ▲ |  |  |  |  |  |  | ▲ |
| 82 | Zhengdao 18 |  | ▲ |  | ▲ |  |  |  |  |  |  | ▲ |
| 83 | Jingnuo 330 |  | ▲ |  | ▲ |  |  |  |  |  |  | ▲ |
| 84 | Zhongjing 212 |  | ▲ |  | ▲ |  |  |  |  |  |  | ▲ |
| 85 | Wuyunjing 21hao |  | ▲ |  | ▲ |  |  |  |  |  |  | ▲ |
| 86 | Huajing 6hao |  | ▲ |  | ▲ |  |  |  |  |  |  | ▲ |
| 87 | Yangfujing 8hao |  | ▲ |  | ▲ |  |  |  |  |  |  | ▲ |
| 88 | Zhendao 99 |  | ▲ |  | ▲ |  |  |  |  |  |  | ▲ |
| 89 | Ningjing 2hao |  | ▲ |  | ▲ |  |  |  |  |  |  | ▲ |
| 90 | Huajing 5hao |  | ▲ |  | ▲ |  |  |  |  |  |  | ▲ |
| 91 | Zhongjing 9677 |  | ▲ |  | ▲ |  |  |  |  |  |  | ▲ |
| 92 | Yangfujing 7hao |  | ▲ |  |  |  | ✔ |  |  |  |  | ▲ |
| 93 | Yuedao 12 | ✔ |  |  | ▲ |  |  |  |  |  | ✔ |  |
| 94 | Jianongnuo 2hao |  | ▲ |  | ▲ |  |  |  |  |  |  | ▲ |
| 95 | Xudao 4hao |  | ▲ |  | ▲ |  |  |  |  |  |  | ▲ |
| 96 | Sihao 4040 |  | ▲ |  | ▲ |  |  |  |  |  |  | ▲ |
| 97 | Yanjing 9hao |  | ▲ |  | ▲ |  |  |  |  |  |  | ▲ |
| 98 | Sihao 4081 |  | ▲ |  | ▲ |  |  |  |  |  |  | ▲ |
| 99 | Sihao 4041 |  | ▲ |  | ▲ |  |  |  |  |  |  | ▲ |
| 100 | Sihao 4031 |  | ▲ |  | ▲ |  |  |  |  |  |  | ▲ |
| 101 | Wandao 68 |  | ▲ |  | ▲ |  |  |  |  |  |  | ▲ |
| 102 | Wuxiang99-8 |  | ▲ |  | ▲ |  |  |  |  |  |  | ▲ |
| 103 | Zhongjing 131 |  | ▲ |  | ▲ |  |  |  |  |  |  | ▲ |
| 104 | Ningjinghui 260 |  | ▲ |  | ▲ |  |  |  |  |  |  | ▲ |
| 105 | Ningjinghui 237 |  | ▲ |  | ▲ |  |  |  |  |  |  | ▲ |
| 106 | Wunuoyihao |  | ▲ |  | ▲ |  |  |  |  |  |  | ▲ |
| 107 | Sihao 4029 |  | ▲ |  | ▲ |  |  |  |  |  |  | ▲ |
| 108 | Wanqu 429bp |  | ▲ |  | ▲ |  |  |  |  |  |  | ▲ |
| 109 | Yangfujing 4901 |  | ▲ |  | ▲ |  |  |  |  |  |  | ▲ |
| 110 | Yandao 6hao |  | ▲ |  | ▲ |  |  |  |  |  |  | ▲ |
| 111 | Cbao |  | ▲ |  |  | ✔ |  |  |  |  |  | ▲ |
| 112 | Zhengdao 10hao | ✔ |  |  |  | ✔ |  |  |  |  | ✔ |  |
| 113 | Baoxintaihuqing |  | ▲ |  | ▲ |  |  |  |  |  |  | ▲ |
| 114 | Huaidao 9hao |  | ▲ |  | ▲ |  |  |  |  |  |  | ▲ |
| 115 | Xiaobaidao |  | ▲ |  | ▲ |  |  |  |  |  |  | ▲ |
| 116 | Yaxuenuo |  | ▲ |  | ▲ |  |  |  |  |  |  | ▲ |
| 117 | Yangguang 200 |  | ▲ |  | ▲ |  |  |  |  |  |  | ▲ |
| 118 | Zaoshirihuangdao |  | ▲ |  | ▲ |  |  |  |  |  |  | ▲ |
| 119 | Luohanhuang |  | ▲ |  | ▲ |  |  |  |  |  |  | ▲ |
| 120 | Xudao2hao |  | ▲ |  | ▲ |  |  |  |  |  |  | ▲ |
| 121 | Xudao9201B |  | ▲ |  | ▲ |  |  |  |  |  |  | ▲ |
| 122 | Ebusinuodao |  | ▲ |  | ▲ |  |  |  |  |  |  | ▲ |
| 123 | Yueguang |  | ▲ |  |  |  | ✔ |  |  |  |  | ▲ |
| 124 | Yimuhu | ✔ |  |  | ▲ |  |  |  |  | ✔ |  |  |
| 125 | Qingkong |  | ▲ |  | ▲ |  |  |  |  |  |  | ▲ |
| 126 | RT61 |  | ▲ |  | ▲ |  |  |  |  |  |  | ▲ |
| 127 | IL38 |  | ▲ |  |  |  | ✔ |  |  |  |  | ▲ |
| 128 | Liuyezhan |  | ▲ |  |  |  |  | ✔ |  |  | ✔ |  |
| 129 | Zaoxian 14 | ✔ |  |  |  | ✔ |  |  |  |  | ✔ |  |
| 130 | Xu91075 | ✔ |  |  | ▲ |  |  |  |  |  | ✔ |  |
| 131 | Xudao 25-7 |  | ▲ |  |  |  |  | ✔ |  |  |  | ▲ |
| 132 | Qing 7 | ✔ |  |  | ▲ |  |  |  |  |  | ✔ |  |
| 133 | Sihao 4141 |  | ▲ |  |  |  |  | ✔ |  |  |  | ▲ |
| 134 | Suwujing | ✔ |  |  | ▲ |  |  |  |  |  | ✔ |  |
| 135 | 9522B |  | ▲ |  | ▲ |  |  |  |  |  |  | ▲ |
| 136 | 863B |  | ▲ |  | ▲ |  |  |  |  |  |  | ▲ |
| 137 | A7444 |  | ▲ |  | ▲ |  |  |  |  |  |  | ▲ |
| 138 | Xiepihuang |  | ▲ |  |  | ✔ |  |  |  |  |  | ▲ |
| 139 | Shengtangqing | ✔ |  |  |  |  |  | ✔ |  | ✔ |  |  |
| 140 | Chuan 6xian | ✔ |  |  |  |  |  | ✔ |  | ✔ |  |  |
| 141 | Chuan 5xian | ✔ |  |  |  | ✔ |  |  |  | ✔ |  |  |
| 142 | Shufeng 101 |  | ▲ |  |  |  |  | ✔ |  |  |  | ▲ |
| 143 | Chengnongshuijing | ✔ |  |  |  |  |  | ✔ |  |  |  | ▲ |
| 144 | Xiangxiandao 10hao | ✔ |  |  |  |  |  | ✔ |  | ✔ |  |  |
| 145 | Ⅱ-32B | ✔ |  |  |  |  | ✔ |  |  |  | ✔ |  |
| 146 | Chenwan 3hao |  | ▲ |  |  |  |  | ✔ |  |  | ✔ |  |
| 147 | Xiangaizao 10hao |  | ▲ |  |  |  | ✔ |  |  |  | ✔ |  |
| 148 | Yuetai B |  | ▲ |  |  | ✔ |  |  |  | ✔ |  |  |
| 149 | Qimiaoxiang 2hao | ✔ |  |  |  |  |  | ✔ |  |  | ✔ |  |
| 150 | Shengyou 2hao |  | ▲ |  |  |  |  | ✔ |  | ✔ |  |  |
| 151 | Guichao 2hao | ✔ |  |  |  |  | ✔ |  |  |  | ✔ |  |
| 152 | LongtepuB | ✔ |  |  |  |  |  | ✔ |  | ✔ |  |  |
| 153 | Hainanxian R | ✔ |  |  |  |  | ✔ |  |  |  | ✔ |  |
| 154 | Zajiaohaigu |  | ▲ |  | ▲ |  |  |  |  |  | ✔ |  |
| 155 | Nuohangu |  | ▲ |  | ▲ |  |  |  |  |  |  | ▲ |
| 156 | Lincangwazuhangu |  | ▲ |  |  |  |  | ✔ |  |  |  | ▲ |
| 157 | Yuedao 55 | ✔ |  |  |  |  |  | ✔ |  |  | ✔ |  |
| 158 | Yuedao 108 |  | ▲ |  |  |  |  | ✔ |  |  |  | ▲ |
| 159 | IR112 | ✔ |  |  |  |  |  | ✔ |  |  | ✔ |  |
| 160 | IR64 | ✔ |  |  | ▲ |  |  |  |  |  | ✔ |  |
| 161 | Gendjah Gempol |  | ▲ |  | ▲ |  |  |  |  |  |  | ▲ |
| 162 | Shengdao 14 |  | ▲ |  |  |  |  |  |  |  |  | ▲ |

✔ indicates the favorable haplotypes; ▲ indicates the non-favorable haplotypes

**Supplementary Table 8**

Table S8.The haplotypes of *LOC _ Os01g43700*, *LOC _ Os09g25784*, and *LOC _ Os04g47890* in 3000 rice genome databases.

| **Candidate genes** | **Haplotypes** | **SNPs** | **Sample List** | **Phenotypes** |
| --- | --- | --- | --- | --- |
| *LOC _Os01g4370*0 | Hap1 | TTTGTCTA | 1213 | (681 of 1213) PL: 24.8cm |
| *LOC _Os01g43700* | Hap2 | TTTGTAGG | 622 | (323 of 622) PL: 24.6cm |
| *LOC _Os01g43700* | Hap3 | TTTGTCGA | 583 | (422 of 583) PL: 24.9cm |
| *LOC_Os09g25784* | Hap1 | CGTCCCCTTAGAGGTATAT | 881 | (477 of 881) PL: 24.9cm |
| *LOC_Os09g25784* | Hap2 | CGTCCCCTCAAGAAGTCGG | 455 | (277 of 455) PL: 24.9cm |
| *LOC_Os09g25784* | Hap3 | GGACCTTCTCAAGATACAT | 336 | (212 of 336) PL: 25.4cm |
| *LOC_Os04g47890* | Hap1 | GGAC | 1069 | (710 of 1069) GW: 3.214g |
| *LOC_Os04g47890* | Hap2 | GTGC | 856 | (668 of 856) GW: 2.917g |
| *LOC_Os04g47890* | Hap3 | GTAC | 746 | (435 of 746) GW: 2.942g |

## 1.2 Supplementary Figures

**Supplementary Figure 1**

**Figure S1.** Manhattan plots of GWAS results for PL, TGP, FGP, SSR, and GWP with the GLM

**
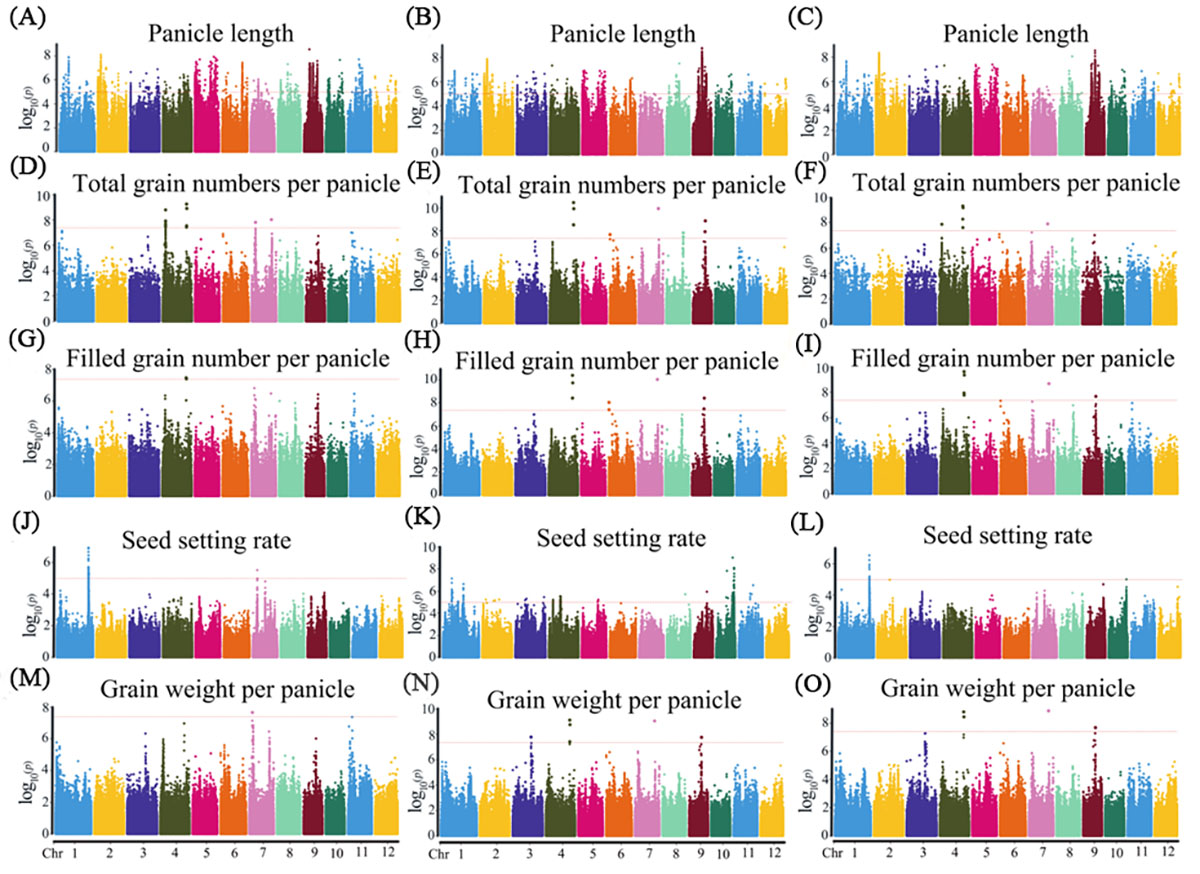
**

**Figure S1**

Manhattan plots of GWAS results for PL, TGP, FGP, SSR, and GWP with the GLM: (A) Manhattan plot for PL in 2019; (B) Manhattan plot for PL in 2020; (C) Manhattan plot for PL in 2021; (D) Manhattan plot for TGP in 2019; (E) Manhattan plot for TGP in 2020; (F) Manhattan plot for TGP in 2021; (G) Manhattan plot for FGP in 2019; (H) Manhattan plot for FGP in 2020; I) Manhattan plot for FGP in 2021; (J) Manhattan plot for SSR in 2019; (K) Manhattan plot for SSR in 2020; (L) Manhattan plot for SSR in 2021; (M) Manhattan plot for GWP in 2019, (N) Manhattan plot for GWP in 2020, (O) Manhattan plot for GWP in 2021.
